# Devices, Functions, and Applications of Artificial Neuromorphic Visual Systems

**DOI:** 10.1002/advs.202516379

**Published:** 2025-11-05

**Authors:** Jiaxin Liu, Bo Li, Chi Liu, Dongming Sun, Huiming Cheng

**Affiliations:** ^1^ School of Material Science and Engineering University of Science and Technology of China 72 Wenhua Road Shenyang 110016 P. R. China; ^2^ Shenyang National Laboratory for Materials Science Institute of Metal Research Chinese Academy of Sciences 72 Wenhua Road Shenyang 110016 P. R. China; ^3^ Institute of Technology for Carbon Neutrality Shenzhen Institute of Advanced Technology Chinese Academy of Sciences 1068 Xueyuan Avenue Shenzhen 518055 P. R. China

**Keywords:** functions and applications, neuromorphic visual systems, optoelectronic synapses, synaptic materials and structures, synaptic performance metrics

## Abstract

Artificial neuromorphic vision systems emulate the biological visual pathway by integrating sensing, storage, and information processing within a unified architecture. Featuring high speed, low power consumption, and superior temporal resolution, they demonstrate significant potential in fields such as autonomous driving, facial recognition, and intelligent perception. As the core building block, the optoelectronic synapse plays a decisive role in determining system performance, which is closely related to its material composition, structural design, and functional characteristics. This review systematically summarizes recent progress in optoelectronic synaptic materials, device architectures, and performance evaluation methodologies. Furthermore, it explores the working mechanisms and network architectures of optoelectronic synapse‐based neuromorphic vision systems, highlighting their capability in image perception, information storage, and target recognition. Current challenges, including environmental stability, large‐scale array fabrication, chip‐level integration, and adaptability of visual functions to real‐world scenarios, are discussed in depth. Finally, the review provides an outlook on future development trends toward stable, scalable, and highly integrated optoelectronic neural vision systems, underscoring their key importance in next‐generation intelligent sensing and information‐processing technologies.

## Graphical abstract

This review highlights recent advances in optoelectronic synapses for artificial neuromorphic vision, emphasizing their material systems, structural designs, and performance metrics. It further discusses visual neural networks enabled by these synapses, covering perception, memory, and recognition functionalities, and analyzes challenges in stability, integration, and system adaptability. Finally, it outlines future directions toward large‐scale, intelligent optoelectronic vision systems.

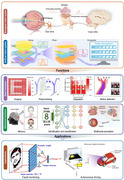

## 1 Introduction

With the rapid advancement of artificial intelligence and machine learning technology, there is an increasing demand for efficient and low‐power computing platforms. By integrating sensing, computation, and memory functions, neuromorphic computing systems can significantly reduce latency and energy consumption during data transmission, thereby enabling more efficient and energy‐saving information processing methods.^[^ ^1^, ^2^ ^]^ Inspired by biological visual systems, neuromorphic visual systems have been extensively studied over the past few decades. Researchers have explored various materials and device architectures to construct artificial neuromorphic visual systems with diverse functions. By designing and fine‐tuning a spectrum of electronic devices, we can faithfully emulate the rich sensory and computational capacities of the human eye and visual system.^[^ ^3^ ^]^

**Figure 1:**
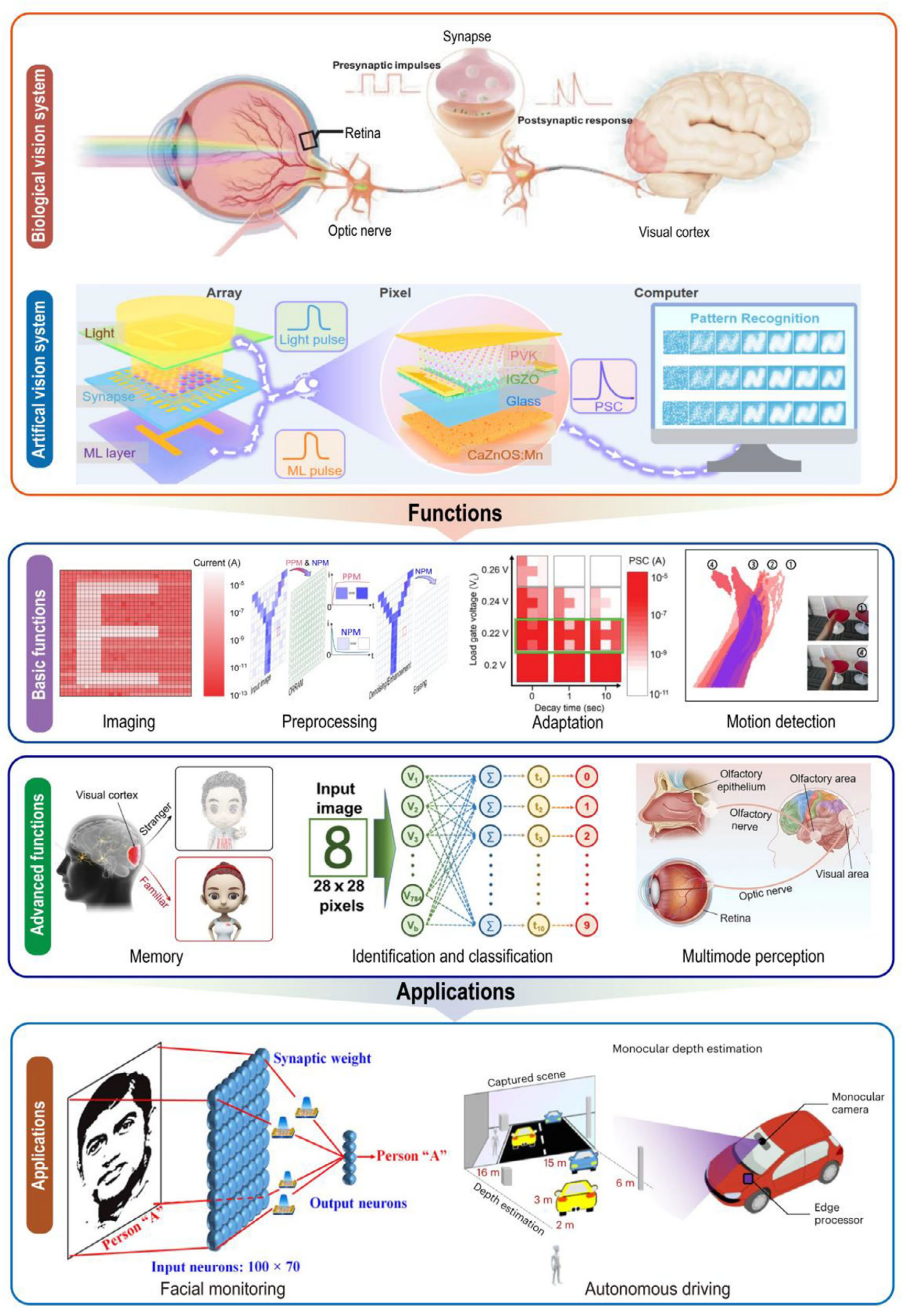
Structures, functions, and applications of a neuromorphic vision system based on optoelectronic synapses.

The working mechanism of human vision is illustrated in **Figure** **1**,^[^ ^4^ ^]^ In the human visual system, rod and cone cells operate in concert to enable color perception and dynamic adaptation across varying light conditions, with the resulting signals undergoing higher‐level processing in the visual cortex. Inspired by this mechanism, neuromorphic visual systems similarly emulate this parallel process of sensing, storing, and processing information.^[^ ^5^, ^6^ ^]^ Such systems offer advantages including high temporal resolution, wide dynamic range, low data redundancy, and low power consumption.^[^ ^7^ ^]^ These systems hold tremendous application potential in fields such as facial recognition, autonomous driving, and machine vision.^[^ ^8^, ^9^, ^10^, ^11^, ^12^ ^]^ The human brain consists of a complex neural network comprising ≈10^11^ neurons and 10^15^ synapses. As a key component of the biological neural system, it exhibits high efficiency, low power consumption, and massive parallelism. Synapses in the brain serve to connect neurons and transmit signals.

Artificial visual systems are designed by mimicking the biological visual system and typically consist of photodetectors, memory units, and processing units. The integration and cooperative operation of these components are essential for achieving efficient information processing.^[^ ^13^, ^14^ ^]^ Artificial neuromorphic vision systems first employ optoelectronic synaptic devices as sensors to capture optical signals, which are then processed in situ or relayed to dedicated signal‐processing arrays. Through weight‐training routines, the system accomplishes tasks such as recognition and classification^[^ ^15^ ^]^ (Figure 1). Optoelectronic synaptic devices are capable of emulating certain characteristics of biological synapses, such as dynamic response behavior, synaptic plasticity, and network connectivity,^[^ ^16^ ^]^ making them fundamental building blocks of neuromorphic computing systems.

In recent years, optoelectronic synaptic devices have made significant progress in functions such as perception, memory, and learning. The perceptive function refers to the ability of the device to detect external image information in real time, resembling the initial visual perception process of the human visual system. It is typically characterized by an enhancement of excitatory postsynaptic current (EPSC) under optical pulse stimulation. The memory function refers to the ability to store visual information and can be categorized into short‐term memory (STM) and long‐term memory (LTM). The transition from STM to LTM can be achieved by prolonging the stimulus duration or increasing the stimulus intensity.^[^ ^17^ ^]^ Learning capability is realized by emulating the plastic behaviors of biological synapses, enabling the system to learn from and be trained on visual information.^[^ ^5^, ^18^, ^19^ ^]^ Artificial visual systems based on optoelectronic synapses not only possess the capability to detect optical information, but also enable real‐time processing of perceptual signals and in situ storage of the results, demonstrating great potential for constructing integrated neuromorphic visual systems with perception‐memory‐computation functionality. Beyond the typical functionalities, neuromorphic visual systems demonstrate intensity‐dependent nonlinear photocurrent relaxation, allowing them to emphasize salient information and suppress background interference.^[^ ^20^, ^21^ ^]^ In addition, such systems can memorize the dynamic trajectory of moving light spots based on time‐dependent photocurrent responses, enabling the construction of spatiotemporal correlations of moving objects on the sensor array.^[^ ^22^ ^]^ The integration of these multidimensional functionalities endows artificial visual systems based on optoelectronic synapses exhibit significant application potential and research value in the field of intelligent perception.

Despite the theoretical advantages of neuromorphic visual systems, numerous challenges remain in practical applications. Current research efforts are primarily directed toward challenges such as synaptic structure design, material selection, device stability, and the efficient conversion of optical images into electrical digital signals.^[^ ^23^ ^]^ Performance metrics such as on/off ratio, endurance, power consumption, and sensitivity are key parameters for evaluating synaptic devices. Optimizing these metrics is crucial for enhancing the overall performance of the system.^[^ ^24^, ^25^, ^26^ ^]^ Furthermore, deciphering the mechanisms and functions of biological visual systems and applying them to artificial visual systems is essential for enabling high‐efficiency image processing. This generally encompasses the emulation of basic visual functions, including imaging,^[^ ^27^ ^]^ image pre‐processing,^[^ ^27^, ^28^ ^]^ visual adaptation,^[^ ^29^, ^30^ ^]^ motion detection,^[^ ^31^, ^32^ ^]^ as well as emulating advanced functions of the visual neural system to achieve image memory,^[^ ^33^ ^]^ image recognition,^[^ ^34^, ^35^ ^]^ and multimodal perception.^[^ ^32^, ^36^, ^37^ ^]^

Through a systematic review, this paper outlines the structure of visual systems, biological and artificial synapses‐including optoelectronic synapses, a type of artificial synapse‐their functions, and related applications. The main content is illustrated in Figure 1. Initially, this review examines the fundamental components of artificial synaptic devices. It focuses on the material systems, device structures, and operational mechanisms, and also provides a summary of key metrics used to evaluate synaptic performance. Building on this foundation, the review further explores the integration of synaptic devices with neural networks. An in‐depth analysis of construction strategies and operational principles of neuromorphic visual systems is also presented. The review then summarizes recent advances in neuromorphic visual systems across representative application scenarios. This discussion provides a comprehensive perspective on their functional diversity and developmental potential. Finally, this review outlines the current research status of neuromorphic visual sensors. Critical challenges are analyzed, and potential directions for future development are identified.

## 2 Optoelectronic Synaptic Device

As a core component of artificial visual systems, the performance of optoelectronic synaptic devices depends heavily on the selection and design of materials and device architectures. The materials and structures directly influence the generation, transport, and recombination of photogenerated carriers, collectively determining the dynamic response and plasticity of the device.^[^ ^38^, ^39^ ^]^ Optoelectronic synaptic memristors and transistors represent the predominant device architectures in this field. Typically configured as two‐terminal structures, optoelectronic memristors integrate photonic and memristive effects, enabling the emulation of biological synaptic behaviors with potential for learning and memory.^[^ ^40^, ^41^ ^]^ Optoelectronic synaptic transistors are typically three‐terminal devices that support dual modulation through both optical stimuli and gate voltage. These devices exhibit high efficiency, low power consumption, minimal crosstalk, and parallel processing capabilities, thereby enabling ultrafast neuromorphic computing.^[^ ^42^, ^43^ ^]^ The evolution of optoelectronic synapses from two‐terminal to three‐terminal architectures has provided greater flexibility and expanded possibilities for synaptic weight modulation.^[^ ^19^ ^]^ The following sections present a detailed discussion of the materials, structures, and performance metrics of artificial synapses. In the following sections, we provide a detailed discussion on the materials, structures, and performance metrics of artificial synapses.

### 2.1 Biological Synapses and Artificial Synapses

**Figure 2:**
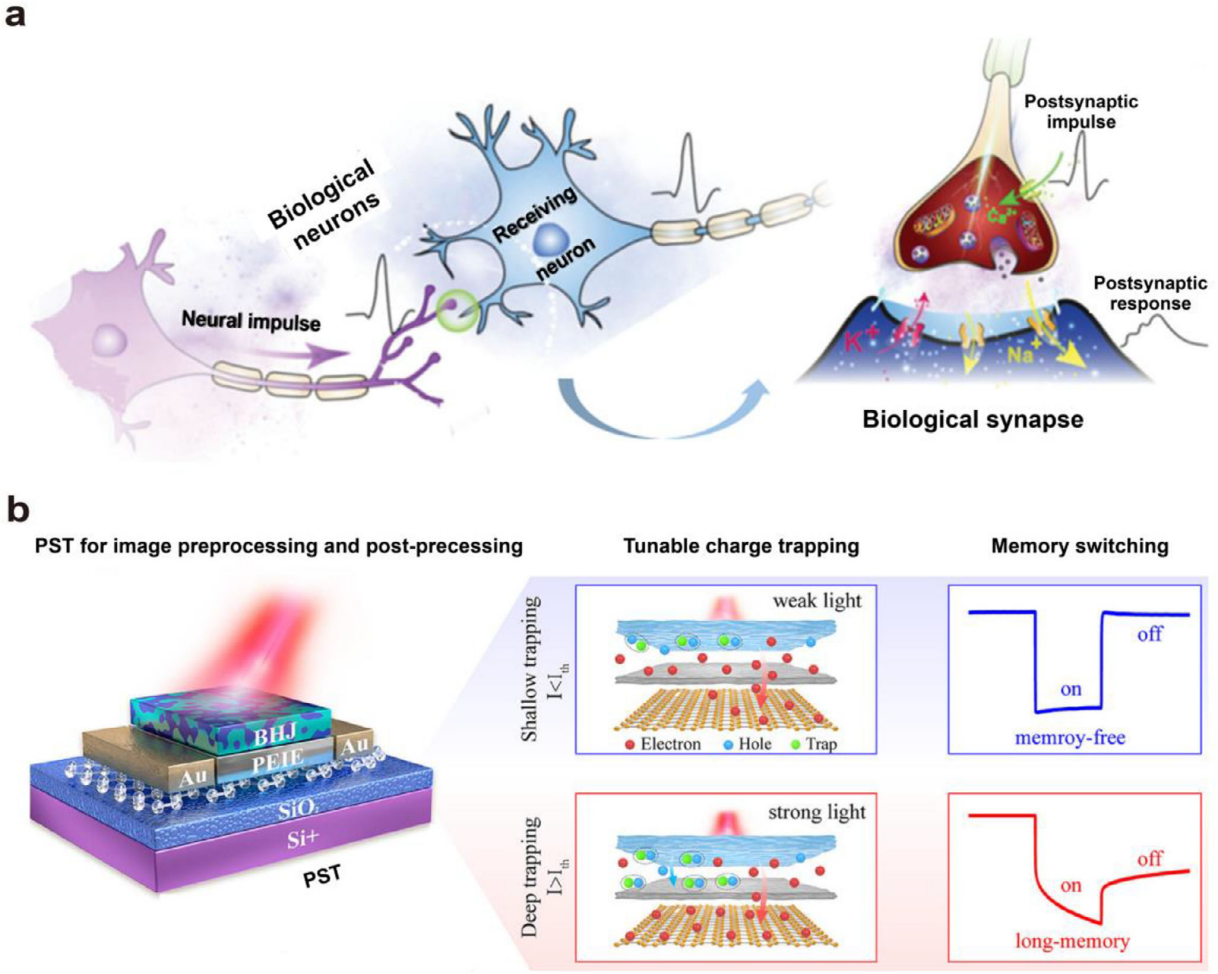
a) Schematic diagram of the human visual perception system.^[^ ^48^ ^]^ b) Schematic diagram of the structure and operation of an artificial synapse.^[^ ^47^ ^]^

The basic unit of the biological nervous system is the synapse, which serves as the junction for information transmission between neurons.^[^ ^4^ ^]^ It is composed of three major components: the presynaptic membrane, the synaptic cleft, and the postsynaptic membrane—as illustrated in **Figure** **2**a. Upon receiving stimulation, the synapse generates an action potential. When this action potential reaches the presynaptic membrane, it triggers the opening of voltage‐gated Ca^2^⁺ channels. Driven by the concentration gradient, Ca^2^⁺ ions flow into the presynaptic terminal. The influx of Ca^2^⁺ promotes the fusion and rupture of synaptic vesicles with the presynaptic membrane, leading to the release of neurotransmitters into the synaptic cleft. These neurotransmitters then diffuse across the cleft and bind to specific receptors on the postsynaptic membrane.^[^ ^44^ ^]^

This binding induces a conformational change in the receptor. As a result, ion channels open, allowing specific ions to flow into or out of the postsynaptic membrane along their concentration gradients. For example, excitatory neurotransmitters such as acetylcholine bind to receptors on the postsynaptic membrane, increasing its permeability to Na⁺ and K⁺. The inward flow of Na⁺ causes depolarization of the postsynaptic membrane, resulting in an excitatory postsynaptic potential (EPSP).^[^ ^45^ ^]^

The postsynaptic neuron typically receives both excitatory and inhibitory postsynaptic potentials (IPSPs) from multiple synapses. Neurons determine whether to generate an action potential by performing spatial summation (summing EPSPs and IPSPs occurring at different locations on the neuron) and temporal summation (summing postsynaptic potentials repeatedly generated at the same synapse over time). If the total excitatory postsynaptic potential reaches the threshold, the postsynaptic neuron will generate an action potential, thereby propagating the signal.^[^ ^46^ ^]^

Artificial synapses have been designed based on the working principles of biological synapses. As shown in Figure 2b, a typical three‐terminal optoelectronic synaptic device exhibits a structure that closely resembles that of a biological synapse. The source and drain correspond to the presynaptic and postsynaptic membranes of a biological synapse, respectively, while the channel represents the synaptic cleft. The optical pulse serves as the presynaptic action potential, and the photogenerated carriers function analogously to neurotransmitters.^[^ ^47^ ^]^ In the absence of optical pulses, the electrical current remains largely unchanged. When subjected to relatively weak optical pulse stimulation, a sudden change in current can be observed. Once the optical stimulus is removed, the current returns to its original state, as shown on the right side of Figure 2b, corresponding to an excitatory postsynaptic potential in a biological synapse. Under stronger optical pulse stimulation, a sudden change in current is observed. After the removal of the optical pulse, the current does not instantly recover to its initial state but instead preserves a fraction of the postsynaptic potential corresponding to the memory effect of biological synapses.

The behavior of a biological postsynaptic neuron, which integrates the spatial and temporal summation of multiple excitatory and inhibitory postsynaptic potentials and determines whether the total EPSPs reach a threshold to generate an action potential, directly corresponds to the weighted summation process of hidden‐layer neurons in a classical feedforward neural network.

### 2.2 Materials and Structures of Artificial Optoelectronic Synapses

The channel materials used for constructing optoelectronic synaptic devices mainly include organic semiconductors, perovskites, metal oxides, and two‐dimensional sulfide materials. Given the distinct advantages of different materials in terms of performance, process compatibility, and device stability, the following sections will analyze the current research status of each material category and their key roles in optoelectronic synaptic devices.

#### 2.2.1 Materials for Artificial Optoelectronic Synapses

##### Organic Semiconductor Materials

**Figure 3:**
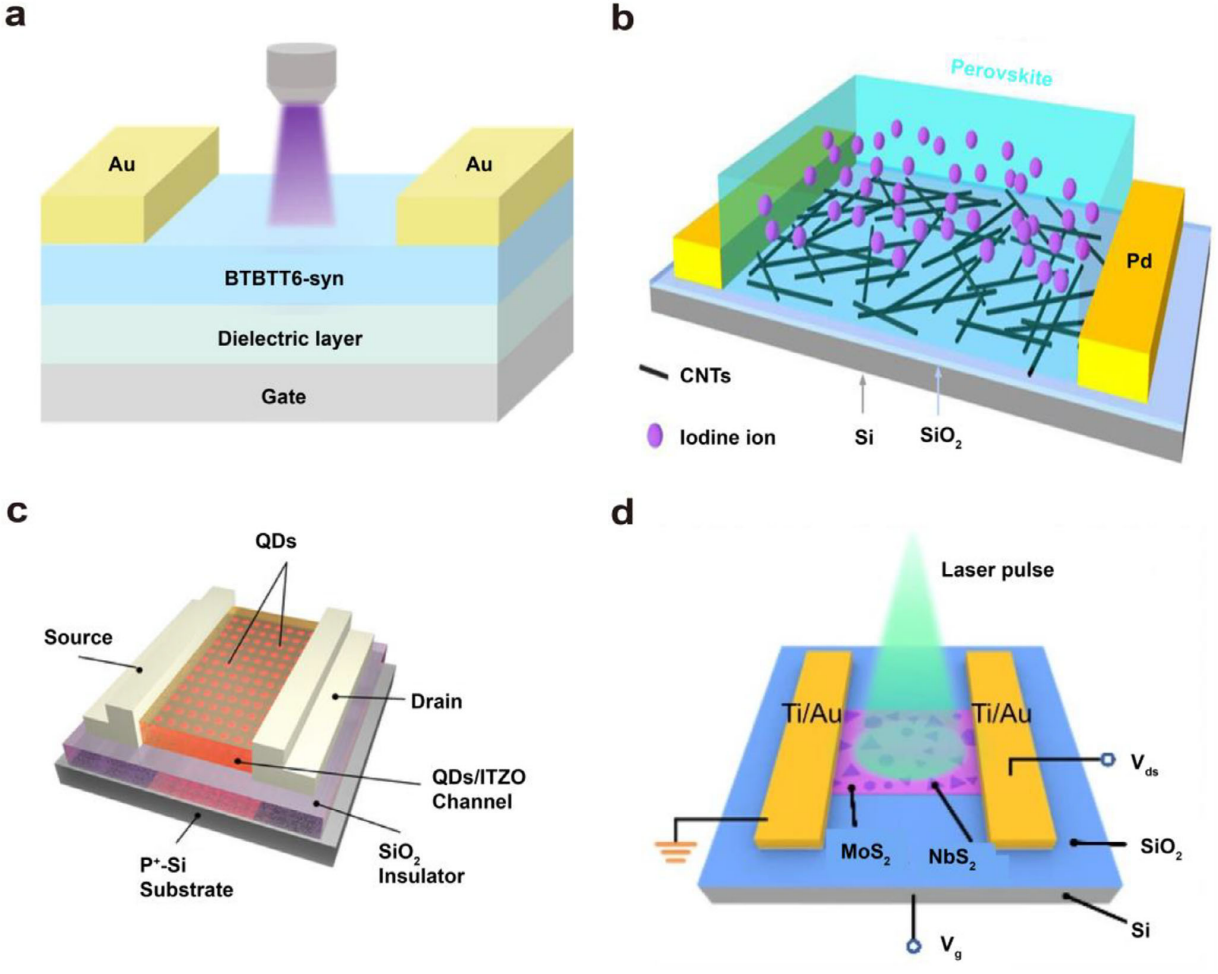
Optoelectronic synapse devices constructed from different types of materials. a) Neuromorphic visual device based on BTBTT6‐syn organic semiconductor material.^[^ ^58^ ^]^ b) Two‐dimensional perovskite/carbon nanotube optoelectronic synapse device based on (PEA)_2_PbI_4_/CNTs.^[^ ^59^ ^]^ c) Optoelectronic synapse device constructed from oxide InSnZnO.^[^ ^55^ ^]^ d) Optoelectronic synapse device constructed from two‐dimensional NbS_2_/MoS_2_ mixed thin film.^[^ ^22^ ^]^

Organic semiconductor materials have attracted considerable attention due to their low energy consumption, tunable optoelectronic properties, low elastic modulus, and high biocompatibility. These materials can serve as conductive channels, light‐absorbing layers, and light‐emitting layers in optoelectronic synaptic devices, enabling the construction of various optoelectronic synaptic architectures. **Figure** **3**a shows an optoelectronic synaptic device fabricated using a typical organic semiconductor material, in which BTBTT6‐syn (2‐hexylthieno[4,5‐b][1]benzo­thieno[3,2‐b][1]benzothiophene) is employed as the channel material. Under ultraviolet illumination, the BTBTT6‐syn film absorbs photons and generates electron‐hole pairs. A large number of hydroxyl groups (e.g., silanol groups) present at the SiO_2_ interface act as electron trap sites, facilitating the separation of the photo‐generated electron‐hole pairs. As a result, the source–drain current increases, enabling the detection of ultraviolet light.

Organic semiconductor synaptic transistors encompass diverse device configurations. Wu et al.^[^ ^49^ ^]^ employed BTBT‐C10 (2‐decyl[1]benzo­thieno[3,2‐b][1]benzo­thiophene) as the channel material and poly(sulfobetaine methacrylate) (PSBMA) as the dielectric layer, utilizing a multi‐ionic system as a substitute for conventional solid electrolytes. The cationic and anionic species are covalently tethered by the flexible alkyl backbone of PSBMA, enabling synaptic functionalities via ion diffusion and charge trapping mechanisms. The constructed device exhibits excellent photoresponse, high capacitance, low operating voltage, and low energy consumption. Wang et al. reported an organic electrochemical transistor with a vertical channel architecture.^[^ ^50^ ^]^ The transistor utilizes either an ionic gel composed of 1‐ethyl‐3‐methylimidazolium bis(trifluoromethylsulfonyl)imide ([EMIM⁺][TFSI^−^]):PVDF‐HFP or an aqueous electrolyte as the gate‐controlling medium. The channel consists of a crystalline‐amorphous polymer blend of PTBT‐p (poly(3‐hexylthiophene)‐co‐(3‐ethylene glycol side chain‐substituted thiophene)), which can be selectively doped by ions. This architecture enables reconfigurable operation between volatile receptor behavior and non‐volatile synaptic functionality. Luo et al. developed a photosensitive heterojunction by selectively doping ionic gel pillars with polypyrrole nanoparticles (PPy‐NPs).^[^ ^51^ ^]^ The resulting pillar array can be directly embedded into the surface of a transparent hemispherical ionic gel, forming a retina‐inspired columnar forest that exhibits both photoelectric conversion capability and neuroelectric plasticity. The designed gel pillar photoreceptors exhibit ultrabroadband optical absorption, highly sensitive photodetection, and persistent photoconductivity. Moreover, they are capable of self‐powered operation, endowing the connected optical synaptic plasticity for encoding multi‐wavelength optical signals.

##### Perovskite Materials

Beyond organic semiconductors, perovskite materials have attracted attention due to their superior light absorption, ionic mobility, and tunable optoelectronic properties, making them promising candidates for neuromorphic optoelectronic devices. For example, optoelectronic devices based on halide perovskite materials have been employed to emulate the light and dark adaptation functions of the human eye. Figure 3b depicts a two‐dimensional perovskite/carbon nanotube‐based optoelectronic synaptic device constructed from (PEA)_2_PbI_4_/CNTs. When the device is exposed to light, the number of photogenerated charge carriers increases, and halide ions in the perovskite (such as iodide ions) become more susceptible to migration under an electric field due to photoexcitation. When a negative back‐gate voltage is applied, iodide ions migrate away from the carbon nanotube surface, leading to the accumulation of positive charges at the surface. This results in an increase in the potential and current of the carbon nanotube.

Wang et al. fabricated an intrinsically stretchable neuromorphic vision‐adaptive transistor (ISNVaT) using a strain‐insensitive viscoelastic CsPbBr_3_ perovskite film.^[^ ^4^ ^]^ The film forms a quasi‐continuous microspherical morphology within an elastomer matrix via a surface energy‐induced strategy, endowing it with inherent stretchability and photosensitivity. Li et al. developed a novel reconfigurable optoelectronic transistor using BaSnO_3_, a material with a perovskite structure, as the channel.^[^ ^52^ ^]^ The device emulates various functions of the biological nervous system, including perception, memory, and information processing. Zheng et al. fabricated ferroelectric tunnel junctions (FTJs) based on perovskite oxides by integrating ultrathin, freestanding ferroelectric perovskite oxide films onto silicon wafers via a wet‐transfer method.^[^ ^53^ ^]^ The resulting devices achieved an on/off ratio as high as 1.2 × 10⁶, a write/erase speed of 1 ns, and endurance exceeding 10⁶ switching cycles. Vasilopoulou et al. summarized approaches for constructing multimodal sensing and computing devices by leveraging the photosensitivity and electronic properties of halide perovskites.^[^ ^54^ ^]^ These materials exhibit both ionic and electronic charge characteristics, enabling their application in adaptive computing systems based on intrinsic device dynamics.

##### Oxide Semiconductor Materials

While perovskites excel in photoresponse and ionic migration, oxide semiconductors offer higher thermal and chemical stability, as well as mature fabrication processes, providing robust alternatives for ultraviolet‐responsive neuromorphic devices. In recent years, oxide semiconductor materials have been widely employed in the fabrication of synaptic devices such as optoelectronic memristors and optoelectronic synaptic transistors, owing to their excellent optoelectronic properties and favorable relaxation characteristics.

Oxide semiconductor materials such as indium tin zinc oxide (ITZO), indium gallium zinc oxide (IGZO), and niobium oxide (NbOx) exhibit excellent thermochemical stability and favorable optoelectronic properties. They also benefit from relatively mature fabrication processes. Although widely applied in optoelectronic neuromorphic devices, these materials primarily exhibit efficient response to ultraviolet light due to their wide bandgaps. Figure 3c presents an optoelectronic transistor with a hybrid channel composed of InP quantum dots (QDs) and ITZO.^[^ ^55^ ^]^ The transistor combines the excellent electrical transport properties of the oxide semiconductor with the superior photoresponse of the InP quantum dots. Upon illumination of the InP quantum dots, electron‐hole pairs are generated. Due to the energy band alignment between the QDs and the ITZO layer, electrons tend to transfer from the QDs to the ITZO, while holes remain in the QDs. By adjusting the gate voltage (*V*_GS_), the electron density in the channel can be modulated, thereby controlling the drain current (*I*_DS_) between the source and drain electrodes.

##### Two‐Dimensional Chalcogenide Semiconductor Materials

Despite the stability of oxide semiconductors, achieving atomic‐scale light‐matter control and flexible integration remains challenging. Two‐dimensional chalcogenide semiconductors address these limitations, enabling novel neuromorphic devices with strong light‐matter interactions and high integration potential. Two‐dimensional chalcogenide semiconductor materials, such as MoS_2_ and NbS_2_, have emerged as promising platforms for the development of novel neuromorphic hardware due to their strong light‐matter interactions, atomically thin thickness, and dangling‐bond‐free surfaces. For example, a NbS_2_/MoS_2_ van der Waals heterostructure has been employed to develop neuromorphic optoelectronic devices and visual sensing arrays with integrated sensing, memory, and computing functionalities. Figure 3d shows the core of a neuromorphic optical sensor based on a two‐dimensional NbS_2_/MoS_2_ hybrid film.^[^ ^22^ ^]^ Each photosensor is configured as a phototransistor based on a field‐effect transistor architecture, fabricated on a SiO_2_ substrate with SiO_2_ serving as the back‐gate dielectric. The photoresponse mechanism of this device is as follows: In the initial stage (Stage I), when no gate voltage is applied, no charge transfer occurs between MoS_2_ and NbS_2_. When a large negative gate voltage is applied, band bending occurs at the NbS_2_/MoS_2_ interface, which facilitates the spatial separation of photoexcited electron‐hole pairs in MoS_2_, thereby leading to an increase in current.

Dang et al. fabricated a prototype visual sensor based on the two‐dimensional material rhenium disulfide (ReS_2_) and the ferroelectric copolymer poly(vinylidene fluoride‐trifluoroethylene) [P(VDF‐TrFE)], integrating recognition, memory, and preprocessing functions within a single device.^[^ ^56^ ^]^ By constructing an array of optoelectronic devices, the system achieved wavelength‐selective object extraction under noisy environments, closely resembling the color discrimination capability of the human retina, and significantly improved image recognition accuracy from 72% to 96%. Cao et al. developed a highly reliable artificial synapse based on a two‐dimensional MoS_2_/GaPS_4_ heterostructure, which remains operational even under exposure to temperatures as high as 400 °C.^[^ ^57^ ^]^ The authors demonstrated high recognition accuracy by implementing a neural network based on the MoS_2_/GaPS_4_ device for MNIST handwritten digit classification in a simulator.

Tan et al.^[^ ^41^ ^]^ adopted a Ta_2_NiSe_5_/SnS_5_ heterojunction as the photosensitive medium. The device ingeniously exploits sulfur vacancies on the SnS_2_ surface to adsorb ambient O_2_ and H_2_O molecules; these adsorbates introduce localized trap states that dramatically lengthen the lifetime of photo‐generated carriers—especially electrons. Because near‐infrared photons carry low energy, carrier generation and trapping are intrinsically inefficient, making long‐duration storage exceedingly difficult. The Physisorption‐Assistant Persistent Photoconductivity (PAPPC) effect surmounts this bottleneck, enabling the device to switch from short‐ to long‐term plasticity even under near‐infrared illumination.

#### 2.2.2 Structures of Artificial Optoelectronic Synapses

The structure of optoelectronic synaptic devices has a significant impact on their performance. During their development, various structural configurations have emerged, including metal‐insulator‐metal stacked structure, heterostructures, floating‐gate structures, vertical crossbar architectures, and strain‐induced structures.

##### Metal–Insulator–Metal Stacked Structure

**Figure 4:**
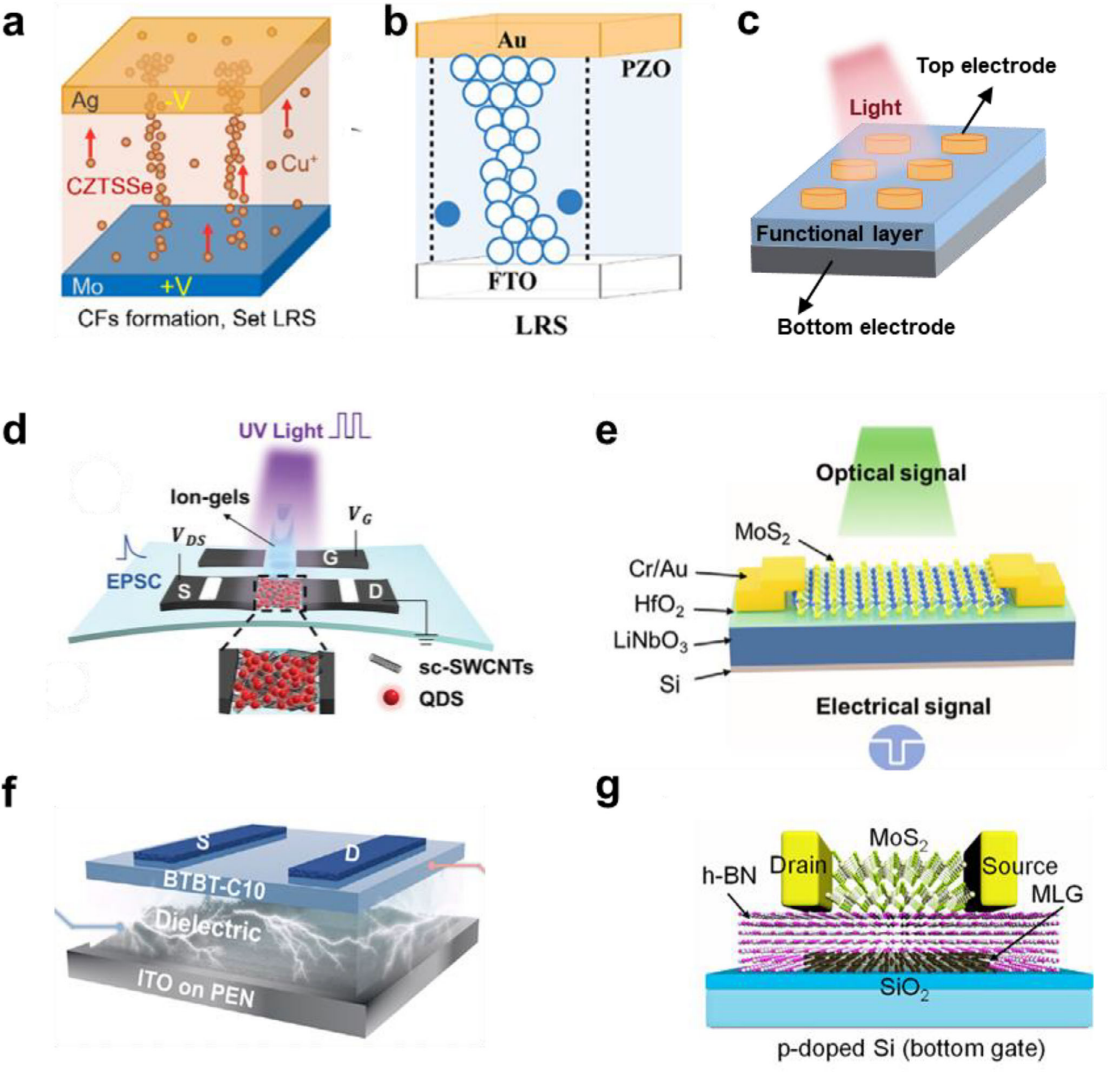
a) Schematic of an Ag/CZTSSe/Mo optoelectronic memristor based on the ECM mechanism.^[^ ^60^ ^]^ b) Schematic of an Au/PZO/FTO optoelectronic memristor based on the VCM mechanism.^[^ ^61^ ^]^ c) Common structures of optoelectronic memristors; Structure of synapse devices. d) Hybrid heterojunction structure composed of single‐walled carbon nanotubes and CdSe/ZnS quantum dots.^[^ ^77^ ^]^ e) Two‐dimensional MoS_2_‐LiNbO_3_/HfO_2_ planar heterojunction structure.^[^ ^78^ ^]^ f) BTBT‐C10/PSBMA organic electrochemical planar heterojunction structure.^[^ ^49^ ^]^ g) MoS_2_/h‐BN/MLG floating‐gate structure.^[^ ^73^ ^]^

Optoelectronic memristors commonly utilize a metal–insulator–metal (MIM) stacked configuration. Their memristive switching behavior primarily arises from the conductive filament mechanism, which is generally divided into two types: electrochemical metallization memory (ECM) and valence change memory (VCM). **Figure** **4**a shows the Ag/CZTSSe/Mo optoelectronic memristor based on the ECM mechanism, whose core is the formation and rupture of Cu⁺ metallic conductive filaments.^[^ ^60^ ^]^ When a negative voltage is applied, Cu⁺ ions migrate and accumulate in the CZTSSe to form a stable metallic filament, switching the device from the high‐resistance state to the low‐resistance state. Under a positive voltage, the filament ruptures at its weakest point, returning the device to the high‐resistance state. Near‐infrared illumination generates electron‐hole pairs that destabilize the filament and facilitate its rupture. Figure 4b illustrates the Au/PZO/FTO optoelectronic memristor based on the VCM mechanism (PZO: PbZrO_3_, FTO: fluorine‐doped tin oxide), whose core is the formation and rupture of oxygen‐vacancy conductive filaments.^[^ ^61^ ^]^ Applying a positive voltage drives randomly distributed oxygen vacancies in the PZO film toward the FTO bottom electrode, where they gradually accumulate into a conductive filament that establishes the low‐resistance state. A reverse voltage ruptures the filament, restoring the high‐resistance state. UV illumination produces a negative photoconductive effect: photogenerated electrons generated in the PZO film are captured by oxygen‐vacancy traps, weakening the conductive filament and decreasing conductance, thereby generating suppression. Gao et al.^[^ ^62^ ^]^ employed a Pt/TeO_x_/Pt stacked memristor in which UV illumination produces a positive photoconductive effect: holes generated by 365 nm UV irradiation are captured by oxygen vacancies, promoting filament formation and thereby producing enhancement. Optoelectronic memristors often adopt the structure shown in Figure 4c. The cylindrical top‐electrode configuration facilitates vertical stacking and is suitable for future high‐density three‐dimensional neuromorphic chip designs.^[^ ^62^ ^]^

##### Heterostructure

Heterojunction structures are among the most representative architectures of optoelectronic synaptic devices. The fundamental basis of synaptic behavior lies in the generation, transport, and recombination of charge carriers. By utilizing the built‐in electric field formed at the heterointerface due to band bending, efficient separation of charge carriers can be achieved while suppressing their recombination, thereby enabling the relaxation characteristics of synaptic devices.

Mixed heterojunctions refer to structures in which two different semiconductor materials are uniformly blended to form a microscale interfacial network. This type of structure is commonly employed in hybrid materials composed of carbon nanotubes (CNTs) and quantum dots.^[^ ^63^ ^]^ On the one hand, the high carrier mobility of carbon nanotubes can be leveraged; on the other hand, the incorporation of quantum dots imparts optoelectronic functionality to the system. The channel material in Figure 4d is a mixed heterojunction composed of semiconducting single‐walled carbon nanotubes (SWCNTs) and CdSe/ZnS quantum dots.^[^ ^64^ ^]^ When light is incident on the CdSe/ZnS quantum dots, electron‐hole pairs are generated. Under an applied bias, holes are extracted into the SWCNTs, whereas electrons remain trapped in the quantum dots. This trapping generates an internal electric field that facilitates further hole injection into the SWCNTs. As a result, the system exhibits a photoresponse and emulates synaptic behavior. Chen et al.^[^ ^65^ ^]^ constructed a hybrid heterojunction by embedding photosensitive VO_2_ nanoparticles into a graphene matrix. Upon photon absorption, VO_2_ generates electron–hole pairs; under an applied bias, the electrons are driven into the graphene channel, giving rise to a pronounced photoresponse. Planar heterojunctions are a type of two‐dimensional structure formed by the lateral arrangement of two different semiconductor materials, creating an interface within the same plane. The synaptic functionalities exhibited by planar heterojunctions composed of different materials are based on varying mechanisms, among which the most common are carrier trapping, electric double‐layer effect, and ferroelectric effect.

The carrier trapping mechanism is one of the most widely used synaptic mechanisms. It involves the capture of photo‐excited or electrically‐excited charge carriers by the dielectric layer or at the interface between the dielectric and the channel. This process hinders the recombination of carriers after the stimulation ends, thereby enabling pronounced synaptic behavior.

Figure 4e shows a synaptic transistor with a two‐dimensional MoS_2_ channel and a LiNbO_3_/HfO_2_ substrate. Upon optical excitation, a portion of the photogenerated carriers is trapped at the MoS_2_/HfO_2_ interface.^[^ ^66^ ^]^ After the removal of optical stimulation, the trapped carriers do not recombine immediately, allowing the device to maintain an elevated current level for a certain period. This enables the storage of optical signals, which is a characteristic feature of the carrier trapping mechanism.

Trung et al. developed a fiber‐shaped inorganic‐organic heterostructure composed of three main components: ZnO nanorods (NRs) serving as the photodetection layer, a depletion layer formed due to the p‐n junction (intermediate layer), and PEDOT:PSS functioning as the output layer.^[^ ^67^ ^]^ When the FPAS is exposed to ultraviolet light with a wavelength of 365 nm, the ZnO nanorods absorb the incident light and generate electron‐hole pairs, leading to electron trapping, hole transfer, and photo‐gating effects. These processes induce changes in the depletion layer between the ZnO NRs and PEDOT: PSS, thereby modulating the conductivity of the PEDOT: PSS layer and giving rise to synaptic behavior.

Chen et al. proposed a carrier‐capture mechanism‐based organic field‐effect transistor (OFET), with a PVK and IDTBT blend as the channel material and PVP as the dielectric layer.^[^ ^68^ ^]^ Under ultraviolet light stimulation, photogenerated electrons and holes are created at the IDTBT/PVP interface. The PVP layer captures a large number of holes (or electrons) under the influence of the electric field, leading to the accumulation of photogenerated electrons (or holes). This results in the formation of a built‐in electric field (*E*_in_) at the IDTBT/PVP and PVP/QDs interfaces, opposite in direction to the external electric field (*E*_out_), thereby achieving synaptic function.

The ferroelectric effect mechanism is also a common operating principle in planar heterojunction synaptic devices, typically involving the use of ferroelectric materials as part of the substrate.^[^ ^56^ ^]^ In Figure 4e, ferroelectric material LiNbO_3_ is used as part of the substrate. As a result, in addition to the carrier trapping mechanism, the device also exhibits a ferroelectric effect mechanism. The underlying principle is that a positive gate voltage can align the polarization of the ferroelectric material downward, leading to the accumulation of electrons in the semiconductor channel and resulting in a low‐resistance state. Conversely, a negative gate voltage aligns the polarization upward, reducing the electron concentration in the channel and leading to a high‐resistance state. When the gate voltage is removed, the slow depolarization of the ferroelectric material caused by the ferroelectric effect gives rise to a hysteresis behavior, which results in synaptic functionality.

The electric double‐layer mechanism essentially functions as a “capacitor” formed at the interface between the dielectric material and the semiconductor channel.^[^ ^69^, ^70^ ^]^ When an ionic electrolyte is used as the dielectric, ions in the electrolyte migrate toward the electrolyte‐semiconductor interface under the electric field induced by the gate voltage, inducing a large number of charge carriers in the channel to form an electric double layer. After the gate voltage is removed, the slow resetting of the ions in the electrolyte leads to a gradual disappearance of the electric double‐layer (EDL), resulting in synaptic behavior. Figure 4f ^[^ ^49^ ^]^ illustrates a typical artificial optoelectronic synapse based on the EDL effect. The device uses 2‐decyl[1]benzo[d]thieno[3,2‐b][1]benzothiophene (BTBT‐C10) as the channel material and poly(sulfobetaine methacrylate) (PSBMA) as the dielectric layer. Under the applied gate voltage, negative ions in the dielectric layer and holes in the channel migrate toward the interface, forming an electric double layer. This results in the separation of holes and electrons in the channel and an increase in the source‐drain current. When the gate voltage is removed, the slow movement of ions‐due to the organic nature of the dielectric‐leads to a gradual disappearance of the electric double layer, thereby inducing synaptic behavior. Gong et al. fabricated a graphene‐based synaptic transistor using graphene as the channel material and an ion gel (Ion‐Gel) as the dielectric.^[^ ^71^ ^]^ When a voltage is applied to the Ion‐Gel gate, an EDL forms at the interface between the graphene channel and the ion gel. This capacitive effect increases the electron concentration in the graphene, thereby modulating its conductivity. Due to the slow ion diffusion in the bottom of the ion gel, the source–drain current (*I*_DS_) decreases gradually after the gate voltage is removed, enabling synaptic functionality.

##### Floating‐Gate Structure

While heterojunctions leverage charge separation and interface effects to emulate synaptic behavior, floating‐gate structures offer an alternative approach by storing photogenerated charges in a dedicated gate layer, enabling nonvolatile synaptic memory. The floating‐gate structure consists, from top to bottom, of the channel, tunneling layer, floating gate, and insulating layer. The floating gate is used to achieve nonvolatile memory behavior. The tunneling layer must be sufficiently thin and insulating to allow photogenerated carriers to tunnel into the floating gate, which captures the charges. The insulating layer serves to isolate the floating gate from the control gate.^[^ ^72^ ^]^ Figure 4g shows a typical floating‐gate transistor,^[^ ^73^ ^]^ employing a 2D layered floating‐gate structure, where MoS_2_ serves as the semiconductor channel, hexagonal boron nitride (h‐BN) acts as the tunneling layer, and multilayer graphene (MLG) functions as the floating gate. By controlling the voltage pulses applied to the gate, induced charge tunnels from the channel into the floating gate. The charge state of the floating gate can be modulated by adjusting the gate voltage, enabling real‐time tuning of synaptic weights and dynamic adjustment of the response to input signals. Hu et al.^[^ ^74^ ^]^ also employed a two‐dimensional layered floating‐gate structure based on MoS_2_/h‐BN/graphene, where MoS_2_ serves as the semiconductor channel, h‐BN as the thin tunneling dielectric layer, and graphene as the floating‐gate terminal. By varying the control gate voltage, the charge state of the floating gate can be modulated, enabling real‐time tuning of synaptic weights. Ning et al.^[^ ^75^ ^]^ employed MoS_2_ as the channel material, HfO_2_ as the tunneling layer, and the ferroelectric material hafnium zirconium oxide (HZO) as the ferroelectric layer. A split‐gate structure was designed such that different regions of the ferroelectric layer undergo partial polarization independently, enabling the integration of both training and inference functions within a single device.

##### Vertical Crossbar Structure

**Figure 5:**
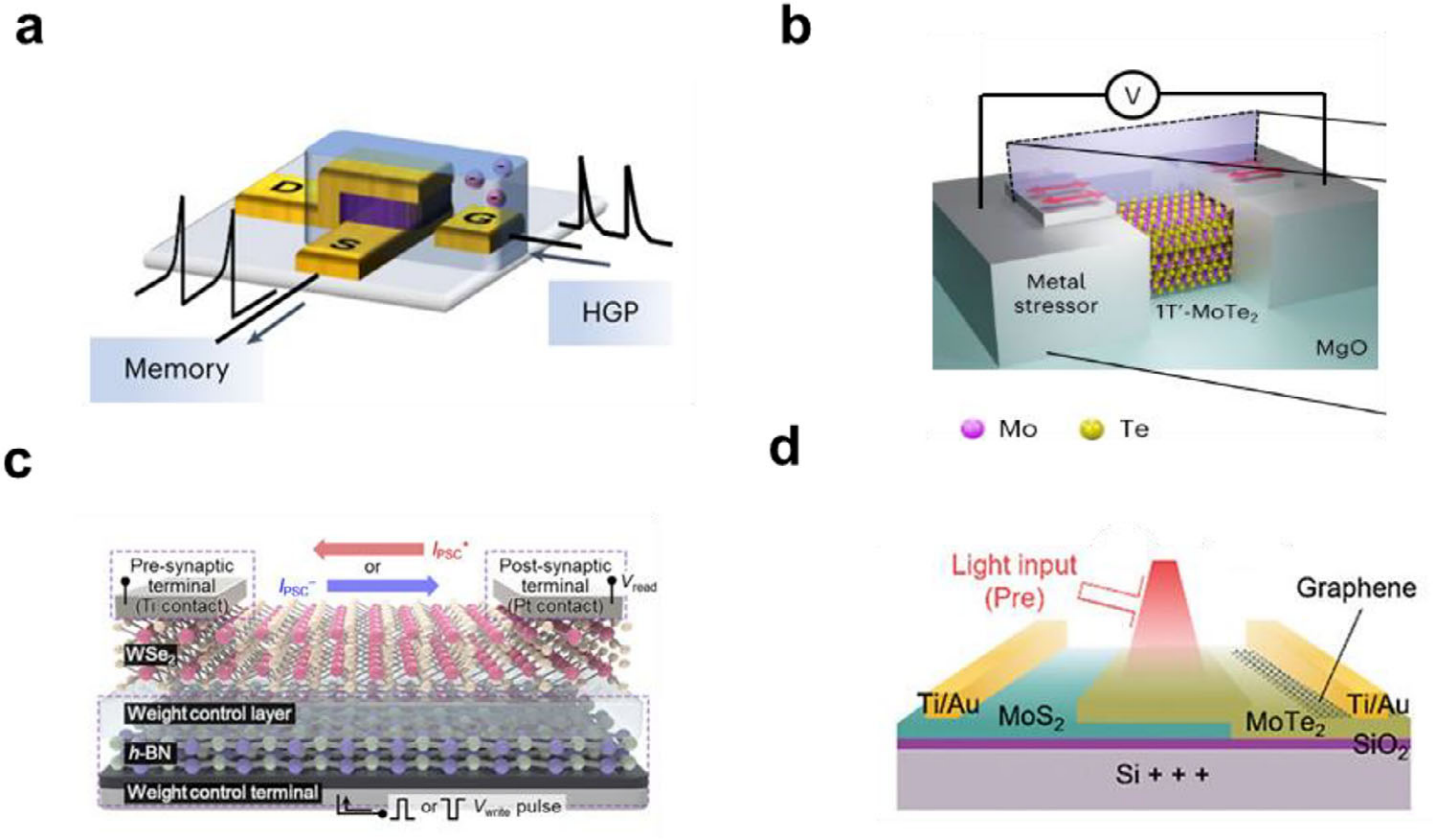
a) Vertical cross‐structured composition of organic mixed ion‐electron conductor PTBT‐p and ionic gel [EMIM⁺][TFSI^−^], PVDF‐HFP.^[^ ^42^ ^]^ b) Multi‐layer MoTe_2_ reversible phase‐change structure.^[^ ^63^ ^]^ c) Optoelectronic synaptic device with an asymmetric Ti/Au/WSe_2_/WCL/h‐BN/Pt/Au metal‐contact structure enabling bidirectional synaptic current.^[^ ^79^ ^]^ d) Novel gate‐tunable dual‐mode optoelectronic synaptic device based on a MoTe_2_/MoS_2_ van der Waals heterostructure.^[^ ^80^ ^]^

In contrast to planar floating‐gate devices, vertical crossbar architectures arrange the channel perpendicular to the substrate, allowing faster ionic response and higher sensitivity in synaptic operations. The vertical crossbar structure constructs the transistor channel in a direction perpendicular to the substrate, as shown in **Figure** **5**a, enabling rapid ionic response and high sensitivity. Wang et al.^[^ ^50^ ^]^ developed a vertical crossbar‐structured organic electrochemical transistor. This vertical architecture enables a more uniform electric field distribution within the channel, facilitating stable ion doping and transport. This configuration enables rapid modulation and recovery of conductivity, which is essential for fast response times in sensors and synaptic devices.

##### Strain‐Induced Structure

Beyond architectural innovations in channel orientation, strain‐induced structures exploit mechanical or electric‐field‐driven phase transitions in the channel material to achieve tunable memristive switching behavior. Hou et al. utilized the mechanism of phase transition between semiconductor and semimetal in MoTe_2_ induced by electric field or strain.^[^ ^76^ ^]^ They stacked MoTe_2_ thin films with stress metal films as the channel material (Figure 5b). The stress metal film applies strain to the MoTe_2_ film, inducing a phase transition from the semimetallic to the semiconducting phase. Under an applied voltage bias, the electric field can further trigger a reversible phase transition between the semiconductor and semimetal phases in MoTe_2_, enabling the switching function of a memristor.

Ahn et al.^[^ ^79^ ^]^ designed an asymmetric metal contact charge trapping layer WSe_2_‐channel optoelectronic synaptic device based on the Ti/Au/WSe_2_/WCL/h‐BN/Pt/Au stack (WCL: weight control layer) that exhibits bidirectional postsynaptic current (Figure 5c). Under illumination, WSe_2_ absorbs photons and generates electron–hole pairs. Owing to the work‐function difference between Ti and Pt (Ti: 4.1 eV, Pt: 5.2 eV), a built‐in electric field is formed, driving photogenerated holes toward the Ti side and electrons toward the Pt side, producing a reverse photocurrent (negative direction). The injection current driven by the bias voltage is in the positive direction; thus, two counter‐propagating current components exist in the baseline environment. Applying voltage pulses to the gate can modulate the charge state in the WCL: when the gate voltage *V*_G_ is positive, electrons in the WSe_2_ channel are captured by the WCL, enhancing the forward current; when *V*_G_ is negative, electrons are released from the WCL, and the negative current dominates.

Ouyang et al.^[^ ^80^ ^]^ proposed a novel gate‐tunable dual‐mode optoelectronic synaptic device based on a MoTe_2_/MoS_2_ van der Waals heterostructure that can simultaneously operate as a self‐powered photodetector and an optoelectronic synapse (Figure 5d). By tuning the gate voltage, the band structure can be modulated to achieve reversible and precise switching between the two modes. A negative gate voltage (−20 V) drives MoS_2_ (n‐type) into a lightly depleted state while MoTe_2_ (p‐type) remains conductive. The MoTe_2_/MoS_2_ heterojunction forms a well‐defined PN junction with the strongest built‐in electric field. Under illumination, electron‐hole pairs are efficiently separated by this built‐in field, generating a pronounced photovoltaic effect that manifests as the photodetector mode. A more negative gate voltage (−40 V) drives MoS_2_ into deep depletion, almost shutting off its channel. At this point, defect states at the MoS_2_/SiO_2_ interface dominate. Under illumination, photogenerated electrons are trapped in these interfacial traps, altering the channel conductance; these electrons are slowly released after illumination ceases, exhibiting synaptic plasticity.

The structural designs of optoelectronic synaptic devices are diverse, including MIM stacked structure heterojunctions, floating‐gate devices, vertical crossbars, and strain‐induced architectures, each relying on distinct mechanisms to realize synaptic functionalities. Given this diversity, a set of common parameters is necessary for the systematic evaluation and comparison of device performance. In the following section, we introduce the key performance evaluation parameters of optoelectronic synapses.

### 2.3 Performance Evaluation Metrics of Artificial Optoelectronic Synapses

As key components in artificial visual perception systems, the performance of optoelectronic synaptic devices largely depends on the synergistic optimization of the material systems and device architectures employed. As sensing‐memory‐computing integration is a crucial application scenario and goal for photoelectric synapses, and numerous review and research papers have elucidated its significance and importance, quantifying it in a scientific manner is particularly essential for organizing the extensive current research. Based on this, metrics such as responsivity and maximum photosensitivity for photodetection, endurance and retention time for memory, as well as on/off ratio and subthreshold swing for computing, should be emphasized. Simultaneously, synaptic plasticity, as an evaluation criterion, is also of paramount importance. This section presents an overview of commonly used performance evaluation metrics for optoelectronic synaptic devices and discusses their physical significance.

#### 2.3.1 On/Off Ratio

The on/off ratio is an important parameter that measures the change in current between the on and off states of an electronic device.^[^ ^81^, ^82^ ^]^ The on/off ratio is defined as the ratio of the current in the on state (*I*_on_) to the current in the off state (*I*_off_) of the device (Equation 1).

(1) $$\mathrm{On}/\mathrm{Off}\;\mathrm{ratio}=\frac{I_{\mathrm{on}}}{I_{\mathrm{off}}}$$

where *I_on_* is the current in the on state, and *I_off_* is the current in the off state.

For optoelectronic synapses and other electronic devices, the on/off ratio is a key performance metric that reflects the device's ability to control current under different operating states. A higher on/off ratio indicates that the device can deliver a larger current in the on state while effectively suppressing current in the off state.^[^ ^83^ ^]^ Currently, the on/off ratio of optoelectronic synaptic transistors can exceed 10⁸.^[^ ^84^ ^]^ In logic circuits and signal processing, a high on/off ratio helps to clearly distinguish between high and low‐level signals, thereby improving signal stability and reliability. Impurities and defects in materials affect the movement of charge carriers by increasing scattering and recombination, which reduces the current in the on state and thus lowers the on/off ratio. Materials with high conductivity and carrier mobility typically exhibit higher on/off ratios.

#### 2.3.2 Subthreshold Swing

Subthreshold swing (*SS*) is a key parameter that measures the gate efficiency and power consumption of a transistor. It represents the change in gate voltage (*V*_G_) required to vary the source‐drain current (*I*_DS_) by one order of magnitude (Equation 2), typically expressed in millivolts per decade (mV dec^−1^).^[^ ^85^ ^]^

(2) $$SS=\frac{dV_{\mathrm{GS}}}{d(log_{10}I_{\mathrm{DS}})}$$

where *V*_GS_ is the voltage between gate and source, typically in mV; *I*_DS_ is the source‐drain current, typically in nA.

A smaller subthreshold swing is preferable, as a lower *SS* value indicates a faster current change in the subthreshold region, leading to a quicker transistor switching speed. This helps achieve lower power consumption because significant current variation can be realized at lower voltages. Materials with high carrier mobility and shorter channel lengths are conducive to reducing the subthreshold swing. However, due to thermal noise, the minimum achievable subthreshold swing is 60 mV dec^−1^ at room temperature.^[^ ^82^ ^]^ This value is determined by Boltzmann's constant, the electronic charge, and temperature, as described in Equation (3). Baek et al.^[^ ^86^ ^]^ introduced a voltage‐reallocation mechanism via the CIPS (CuInP_2_S_6_) threshold switch in a synaptic transistor by integrating a CIPS threshold switch in series with a MoS_2_ FET: when CIPS is in the high‐resistance state, *V*_DS_ mainly drops across CIPS, and the MoS_2_ channel experiences a small bias; once CIPS switches to the low‐resistance state due to Cu^+^ migration, the voltage is rapidly transferred to the MoS_2_ channel, turning it on swiftly. This abrupt resistance transition accelerates the FET turn‐on process, thereby surpassing the thermal‐electron limit and achieving a subthreshold swing below 60 mV dec^−1^.

(3) $$SS_{\min}=\frac{kT}{q}\ln\left(10\right)\approx 60\;\mathrm{mV}\;\mathrm{dec}-1$$

where *k* is the Boltzmann constant, usually taken as 1.38×10^−^^2^ ^3^ J K^−1^ (Joules per Kelvin); *T* is the absolute temperature in K; *q* is the electron charge, usually taken as 1.602×10^−19^ C (Coulombs).

#### 2.3.3 Power Consumption

In neuromorphic computing, the power consumption of a synapse typically refers to the energy consumed during a single synaptic event (i.e., a synaptic pulse). The power consumption of synaptic devices is a critical parameter for evaluating their efficiency and practicality in real‐world applications. Low power consumption is a fundamental requirement for neuromorphic hardware, especially in portable and embedded systems.

Traditional CMOS circuits consume approximately 900 pJ of energy to simulate a single synaptic event,^[^ ^87^ ^]^ whereas the human brain achieves large‐scale, efficient parallel information processing with an energy consumption of only 1–100 fJ per synaptic event.^[^ ^88^ ^]^ Currently, advanced artificial synapses have reduced the energy consumption per event to around 0.1 fJ, operating at voltages as low as 0.0001 V. The power consumption is typically calculated as shown in Equation (4), which considers only the electrical response energy.

(4) $$Q=\int_{t_{0}}^{t_{1}}V\cdot I\left(t\right)dt$$

where *Q* is the energy consumption per synaptic event, *t_0_* and *t_1_* represent the start and end times of the optical stimulation (*∆t* *=* *t_1_ − t_0_*), I is the device current, and *V* is the voltage applied to the device.

#### 2.3.4 Endurance

Endurance refers to the ability of a memory device to maintain its critical performance parameters after many write and erase operations; it is expressed as the number of cycles, i.e., how many write/erase operations can be performed before the device performance degrades to 90 % of its initial value.^[^ ^89^ ^]^ High endurance means that a device can operate reliably over the long term under frequent data updates and modifications. This is particularly important for application scenarios that require frequent data rewriting, such as machine learning training

Endurance testing is typically performed by applying continuous write and erase voltage pulses. During the test, the current‐voltage characteristics of the device are periodically measured to monitor performance changes. Current endurance test results for non‐volatile memory devices show that some high‐performance non‐volatile memories can withstand over 10^13^ cycles, whereas conventional flash memory typically endures only around 10⁴ to 10⁵ cycles.^[^ ^90^ ^]^

#### 2.3.5 Retention Time

Retention time refers to the duration during which a memory device can preserve data without power supply. It is usually expressed in units of seconds. A long retention time indicates that the device can maintain data stability over an extended period, which is crucial for applications requiring long‐term data storage, such as data backup and historical records. It directly affects data reliability and the practicality of the memory device.

Retention time, originally a key metric for storage devices, is also crucial in synaptic devices, as it directly reflects the ability to emulate biological long‐term memory (LTM). It is defined as the time, in seconds (s), during which the synaptic weight remains within ±10 % of its programmed initial value.

Retention time testing is typically conducted by placing the memory device under specific environmental conditions (such as high temperature and high humidity) and periodically reading the data to check for any changes. For accelerated testing, the aging process can be sped up by increasing the temperature, enabling the evaluation of long‐term retention within a shorter period. Some high‐performance non‐volatile memories can retain data for over 10 years, whereas typical flash memory may only maintain data for a few years.^[^ ^77^ ^]^

#### 2.3.6 Responsivity

Responsivity (*R*) is an important metric that measures the sensitivity of optoelectronic synaptic devices to optical signals. It is defined as the ratio of the output electrical current of the optoelectronic device to the input optical power (Equation 5), representing the amount of photocurrent generated per unit of incident optical power, with units of A W^−1^.

(5) $$R=\frac{I_{\mathrm{ph}}}{P_{in}}$$

where *R* is the responsivity, *I*_ph_ is the net photocurrent, and *P*_in_ is the optical power.

By measuring responsivity, one can understand the magnitude of the photocurrent generated by the device under different light intensities, thereby assessing its sensitivity to optical signals. Currently, responsivity values as high as 10⁷ A W^−1^ have been achieved. For applications requiring operation under low‐light conditions, such as night vision imaging systems and weak signal detection in optical communications, high‐responsivity optoelectronic synaptic devices can more effectively detect faint optical signals, thereby enhancing the system's detection capability.^[^ ^5^ ^]^

#### 2.3.7 Specific Detectivity

Specific detectivity (D*) quantifies a photodetector's ability to discern weak optical signals at a given wavelength; it represents the signal‐to‐noise ratio that the device can achieve under specified conditions.^[^ ^91^ ^]^ Defined by Equation (6), it describes the detector's capability to sense the weakest optical signal per unit area and per unit bandwidth. A higher D* indicates a stronger theoretical response to extremely weak light signals.^[^ ^92^ ^]^

(6) $$D^{*}=\frac{\sqrt{A\cdot \Delta f}}{NEP}$$

where *A* is the effective photosensitive area of the detector, in cm^2^; Δ*f* is the operating bandwidth of the measurement system, in Hz; and NEP is the noise‐equivalent power—the minimum incident optical power required to yield a unity signal‐to‐noise ratio—usually expressed in W ${\sqrt{\mathrm{HZ}}}^{-1}$.

For practical photodetector evaluation, Equation (7) is often simplified to the SNR obtained when 1 W of radiant power illuminates a detector with 1 cm^2^ photosensitive area and the output is measured with a 1 Hz bandwidth circuit.^[^ ^93^ ^]^ It is commonly used to evaluate the performance of detectors under low‐light conditions, and its unit is Jones (cm·Hz^1/2^·W^−1^). Generally, devices with excellent weak‐light detection capabilities can achieve specific detectivities above 10^13^ Jones, with the highest values reaching up to 10^16^ Jones.^[^ ^94^ ^]^

(7) $$D^{*}=\frac{R\cdot \sqrt{A}}{S_{n}^{1/2}}$$

where *R* is the responsivity, *A* is the area of the detector, and *S*_n_ is the noise power spectral density, representing the noise power of the detector per unit bandwidth, with units of watts per hertz (W/Hz). In practical scenarios, the noise current i_n_ is often used for calculation.

(8) $$D^{*}=R\cdot \sqrt{\frac{A}{2eI_{dark}}}$$

where *R* is the responsivity, i.e., the photocurrent generated per watt of incident optical power, in A W^−1^; *A* is the detector area, usually in cm^2^; e is the elementary charge, a constant of approximately 1.6 × 10^−19^ C; and *I*_dark_ is the dark current—the current in the absence of light in A.

#### 2.3.8 Maximum Photosensitivity

Similar to the on/off current ratio, the maximum photosensitivity (*P*_max_) refers to the maximum ratio of the photocurrent to the dark current of a photodetector at a specific wavelength, as defined in Equation (9). *P*_max_ reflects the detector's response capability under the strongest illumination conditions. A higher value indicates greater sensitivity of the photodetector to optical signals.^[^ ^95^ ^]^ In recent years, the maximum photosensitivity of optoelectronic synaptic devices has reached ≈1.53 × 10⁸.^[^ ^96^ ^]^

(9) $$P_{\max}=\frac{I_{\mathrm{ph}}}{I_{\mathrm{dark}}}$$

where *P*_max_ is the maximum photosensitivity, *I*_ph_ is the current under illumination, and *I*_dark_ is the background current under dark conditions.

#### 2.3.9 Response Time

The response time of an optoelectronic synapse refers to the time interval between the detection of a stimulus and the accurate output of a spike, and is commonly expressed as the time required for the output voltage or current to rise from 10% to 90% of its maximum value. It is commonly used to evaluate the reaction speed of the synaptic device upon receiving an input signal. A shorter response time indicates that the device can quickly adapt to environmental changes, emulating the rapid response characteristics of biological neural systems.

The response time is governed by the intrinsic properties of the material: the higher the carrier mobility, the faster the carriers move and the shorter the response. It is fundamentally determined by the efficiency of photon‐to‐charge conversion and the subsequent charge dynamics inside the semiconductor—namely, how rapidly photogenerated electron–hole pairs are created, separated, and transported to the electrodes—making it one of the core parameters of an optoelectronic synapse.

#### 2.3.10 Synaptic Plasticity

Functions such as perception, memory, and learning are manifestations of photosensitivity and synaptic plasticity, and they form the foundation for constructing efficient neuromorphic visual systems. Perception enables optoelectronic synapses to receive and process external information; memory allows this information to be stored and retained; and learning enables the system to self‐adjust and optimize based on the acquired information. To achieve better performance in perception, memory, and learning, evaluation metrics for synaptic plasticity have also been developed accordingly.

##### Postsynaptic Current

**Figure 6:**
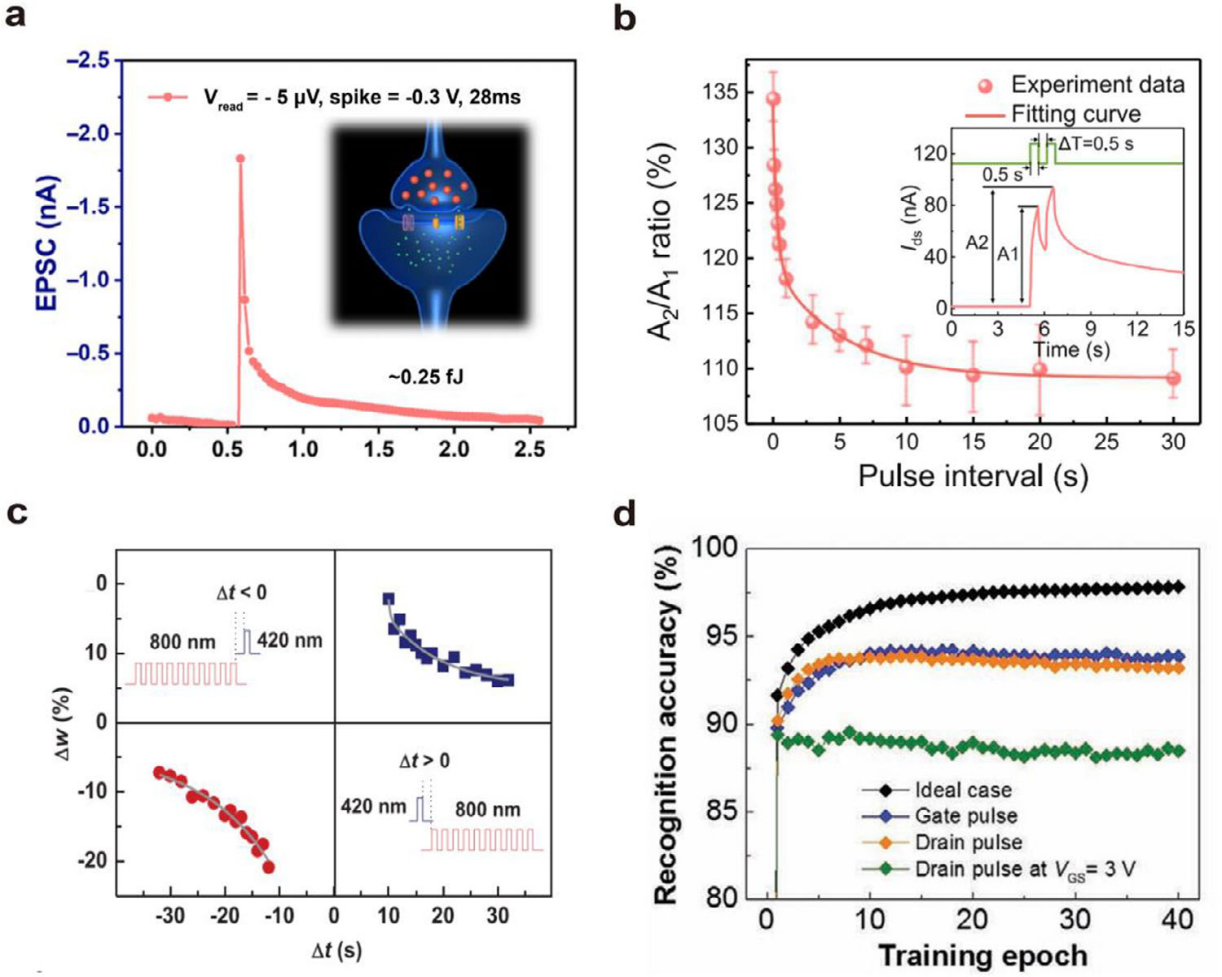
Basic characteristics of optoelectronic synapses. a) Postsynaptic current, structure, and power consumption of optoelectronic synapses.^[^ ^49^ ^]^ b) Paired‐pulse facilitation and its change with stimulation pulse interval.^[^ ^22^ ^]^ c) Spike‐timing‐dependent plasticity of synapses and the effect of stimulation frequency on synaptic weight.^[^ ^56^ ^]^ d) Accuracy of signal recognition by synaptic arrays.^[^ ^100^ ^]^

Perception refers to the ability to detect external images in real time through image sensors, similar to the human visual system. In artificial optoelectronic synapses, this is manifested as an increase in the postsynaptic current (*I*_PSC_) in response to light pulse stimulation, as shown in **Figure** **6**a.^[^ ^49^ ^]^ By maintaining a constant drain‐source voltage (*V*_DS_) across the artificial visual neuromorphic transistor and applying either light pulse stimulation or gate voltage, the postsynaptic current (*I*_PSC_) initially rises rapidly and then gradually decreases.

Generally, the *I*_PSC_ of synaptic devices is related to the trapping and release of charge carriers and can be explained using the stretched exponential model. The decay of *I*_PSC_ over time can be fitted by the Kohlrausch stretched exponential function,^[^ ^97^, ^98^ ^]^ as expressed in Equation (10).

(10) $$I_{psc}=I_{0}\exp\left[-{\left(\frac{t}{\tau }\right)}^{\beta }\right]+I_{\infty }$$

Here, *I*_0_ denotes the prefactor, *τ* is the characteristic relaxation time, *β* is the stretching exponent (ranging from 0 to 1), and *I_∞_* represents the steady‐state current level. A larger *τ* indicates a slower decay of *I*_PSC_, meaning the material can maintain a higher current or signal intensity for a longer duration. The *β* value close to 1 suggests that the *I*_PSC_ decay approximates a simple exponential decay, which is relatively straightforward and rapid. Conversely, a smaller *β* indicates a more complex decay process with a slower decay rate and a pronounced long tail. In neural networks requiring long‐term memory, materials are expected to exhibit larger *τ* and smaller *β* values after fitting; whereas in systems demanding rapid information processing, materials should have smaller *τ* and *β* values close to 1.

##### Paired‐Pulse Facilitation

Paired‐pulse facilitation (PPF) is a quantitative index used to describe the paired‐pulse facilitation phenomenon in neural synapses, representing the ratio of the postsynaptic currents (PSC) induced by two consecutive pulses (Equation 11). The short‐term memory performance of a device is generally evaluated by the PPF index. A PPF value greater than 1 indicates the presence of paired‐pulse facilitation, meaning that the response triggered by the second pulse is stronger than that induced by the first pulse.

(11) $$\mathrm{PPF}=\frac{A_{2}}{A_{1}}$$

Here, *A*_1_ represents the amplitude of the postsynaptic current or potential induced by the first pulse, and *A*_2_ represents that induced by the second pulse.

The dependence of the PPF index on the inter‐pulse interval follows a bi‐exponential function, as expressed in Equation (12).

(12) $$\mathrm{PPF}=C_{1}\exp\left(-\frac{\Delta t}{\tau_{1}}\right)+C_{2}\exp\left(-\frac{\Delta t}{\tau_{2}}\right)$$

Here, Δ*t* denotes the time interval between two consecutive stimuli; *C*_1_ and *C*_2_ are constants representing the initial PPF values at different time scales; *τ*_1_ and *τ*_2_ are two distinct time constants corresponding to the fast and slow decay processes, respectively.

A larger PPF index contributes to enhanced sensitivity and response speed of neural networks, while a smaller PPF index may result in sluggish responses to rapidly changing input signals, which is detrimental to fast signal transmission and processing. However, an excessively large PPF index may lead to excessive accumulation and amplification of signals, thereby increasing the risk of signal overload.

Figure 6b illustrates the phenomenon of paired‐pulse facilitation (PPF),^[^ ^22^ ^]^ which refers to the enhanced postsynaptic response elicited by two successive synaptic stimuli. The shorter the interval between the two stimuli, the more pronounced the PPF effect^[^ ^56^ ^]^ and the higher the PPF index (A_2_/A_1_). In biological neural systems, PPF plays a critical role in recognizing and decoding temporally resolved information, such as visual and auditory signals.

Memory functionality refers to the ability to store visual information, which can be categorized into short‐term memory (STM) and long‐term memory (LTM). With prolonged stimulation duration and increased stimulus intensity, the transition from STM to LTM can be achieved. By modulating the duration and voltage of a single synaptic stimulus (corresponding to the optical stimulus intensity),^[^ ^52^ ^]^ the synaptic weight can gradually increase from weak to strong, enabling the transition from STP to LTP.

##### Spike‐Timing‐Dependent Plasticity

Spike‐timing‐dependent plasticity (STDP) is a form of synaptic plasticity observed in biological neural systems. STDP depends on the relative timing of spikes (action potentials) between pre‐ and postsynaptic neurons. It is one of the key mechanisms underlying learning and memory processes, allowing the neural system to adjust synaptic weights based on the temporal relationship of input signals.

(13) $$\Delta W=A\exp\left(-\frac{\left|t_{\mathrm{pre}}-t_{\mathrm{post}}\right|}{\tau }\right)$$

The basic principle of STDP is illustrated in Equation (13) and Figure 6c.^[^ ^99^ ^]^ STDP refers to the change in synaptic weight (Δ*W*) resulting from the variation in the timing and interval (±∆*t*, ∆*t* = *t*_pre_ − *t*_post_) between pre‐ and postsynaptic neural activities. When the presynaptic spike arrives before the postsynaptic spike, the synaptic weight increases, resulting in long‐term potentiation (LTP) (Figure 6c, top right). Conversely, when the postsynaptic spike precedes the presynaptic spike, the synaptic weight decreases, leading to long‐term depression (LTD) (Figure 6c, bottom left).

The learning function refers to the ability to simulate biological synaptic plasticity to enable learning and training, particularly through spike‐timing‐dependent plasticity (STDP). Successful emulation of STDP has become a critical benchmark for evaluating hardware systems of spiking neural networks (SNNs). As shown in Figure 6d,^[^ ^52^ ^]^ when reading under the same source‐drain voltage (*V*_DS_), the visual neuromorphic transistor generates a higher postsynaptic current after training than before training.

##### Recognition Accuracy

Recognition accuracy is a key metric for evaluating the performance of classification models. It is often closely related to the number of training epochs and represents the proportion of correctly classified samples to the total number of samples under a given training period.

Generally, recognition accuracy gradually improves with the increase in training epochs. This is because the neural network continuously adjusts its weights through multiple training iterations to better fit the training data. Achieving high accuracy within fewer training epochs is a key goal for optoelectronic synapse arrays.

Figure 6d lists several recent studies on recognition accuracy and their corresponding training epochs. From the figure, it can be seen that currently, some of the more advanced devices can achieve over 95% accuracy within 10 epochs.^[^ ^15^ ^]^

To more intuitively present the physical significance of various optoelectronic synapse performance parameters and their mainstream implementation levels in current research, we have classified and organized the relevant metrics, as detailed in **Table** **1**.

**Table 1:** Performance evaluation indicators for optoelectronic synapses.

Classification	Performance metrics	Function	Materials: Current level	Comparison	Refs.
Perception function evaluation metrics	On/Off ratio	Indicates the efficiency with which the device converts optical signals into electrical signals	ITO(Sn‐doped In_2_O_3_)/HZO(Hf_0.5_Zr_0.5_O_2_): ≈10^8^	These two parameters all gauge the device's computational potential, each with a specific emphasis: on/off ratio focuses on the difference between conduction and cutoff currents; subthreshold swing concerns switching efficiency—how easily the device turns on and off.	[101]
			BTO(BaTiO_3_)/LSMO(La_0.67_Sr_0.33_MnO_3_): ≈10^7^		[102]
			ReS_2_/CIPS(CuInP_2_S_6_): ≈10^6^		[37]
	Subthreshold swing (*SS*)	Quantifies the detector's ability to sense weak optical signals	MoS_2_/ CIPS(CuInP_2_S_6_): ≈7.5 mV dec^−1^		[86]
			WSe_2_/MoS_2_: ≈ 60 mV dec^−1^		[82]
			PTBT‐p/[EMIM^+^][TFSI^−^], PVDF‐HFP: ≈ 65 mV dec^−1^		[84]
Storage function evaluation metrics	Endurance	The number of write/erase cycles the device can sustain while remaining stable	MoS_2_/HZO(Hf_0.5_Zr_0.5_O_2_)/HfO_2_: > 10^13^ cycles	These two parameters both assess the device's storage function, each with a different emphasis: endurance addresses operational lifetime—how many cycles can be performed; retention time concerns storage duration—how long information can be preserved.	[90]
			NbO_x_/Nb: > 10^13^ cycles		[103]
			MoTe_2_‐ MoS_2_/h‐BN/Graphene: > 10^6^ cycles		[104]
	Retention time	Quantifies how long the device retains data after power is removed	GST(Ge_2_Sb_2_Te_5_): >10 year		[105]
			HZO(Hf_0.5_Zr_0.5_O_2_): >10 year		[77]
			CuSbS_2_: >8×10⁷ s		[106]
Computing function evaluation metrics	Responsivity (*R*)	Measures the on/off current ratio	CsPbBr_3_‐CNTs: ≥10^7^ A W^−1^	These three parameters all evaluate the device's perception function, each with a distinct emphasis: responsivity focuses on conversion efficiency—how much electrical output is produced per optical input; specific detectivity concerns the detection limit and sensitivity, i.e., the weakest optical signal that can be sensed; maximum photosensitivity addresses the upper limit of light‐sensing performance, namely the peak responsivity under given conditions.	[5]
			GaN PNA: ≈2×10^6^ A W^−1^		[91]
			FASnI_3_/PEA_2_FASn_2_I_7_: ≈6.8 × 10^5^ A W^−1^		[107]
	Specific detectivity (D*)	The voltage required to change the current by one order of magnitude	MoS_2_/Graphene/Al_2_O_3_, SiO_2_: 1.08×10^16^ ≈ 5.85×10^16^ Jones		[94]
			InGaN/GaN: ≈1.56×10^14^ Jones		[108]
			C8‐BTBT:PC71BM/MoO_3_/P(VDF‐TrFE): ≈1.3 × 10^13^ Jones		[109]
	Maximum photosensitivity (*P*_max_)	Measures the difference between photocurrent and dark‐environment current	Ph‐BTBT‐10/PS/F_4_TCNQ: 1.53 × 10⁸		[96]
			BTBTT6‐syn: ≥10^5^		[95]
			PEA_2_SnI_4_/PDPP‐DTT:PC_61_BM: ≈4.9×10^5^		[110]
Cross‐dimensional metrics	Power consumption	The energy consumed in a single synaptic event (synaptic spike).	DNTT/Py‐SAM, F4BCF ≈ 2.96×10^−4^ fJ/event	These three parameters are related to at least two of the functions, such as perception, storage, and computation. Power consumption addresses the energy used during the sense‐store‐compute process; Recognition accuracy is the system's benchmark; Synaptic plasticity is the foundation for the sense‐store‐compute process in optoelectronic synaptic devices;	[111]
			sc‐SWCNTs/CdSe/ZnS QDs: ≈0.015 fJ/event		[77]
			O‐FGT(Fe_3_GeTe_2_): ≈2 fJ/event		[112]
	Recognition accuracy	Quantifies the recognition capability of a system built from the devices	MoS_2_/HfO_2_/Au: ≥95%/10 epoch		[15]
			PDPP4T/NTCDI‐F15: ≈93%/30 epoch		[113]
			ReS_2_/h‐BN/Gra: 98.8%/200 epoch		[114]
	Synaptic plasticity	Characterizes synaptic efficacy using postsynaptic current (EPSC), paired‐pulse facilitation (PPF), and spike‐timing‐dependent plasticity (STDP)	/		[52, 56]

## 3 Functions of the Artificial Neuromorphic Vision System

Neuromorphic vision systems based on optoelectronic or visual synaptic arrays have gradually acquired complex functions akin to those of the human eye and biological vision through continuous development and advancement. Building upon the capabilities of imaging, image memory, recognition, and classification, they have evolved to include important functions such as motion detection, image pre‐processing, visual adaptation, and multimodal perception.

### 3.1 The Role of Artificial Optoelectronic Synapses in Neuromorphic Visual Systems

In biological visual systems, a large number of synapses coordinate the perception, transmission, processing, and recognition of visual information by modulating the connections between arrays of neurons. Inspired by this, artificial vision systems simulate the structure and function of biological vision systems and are mainly categorized into two types: hardware‐based artificial neural vision and software‐based artificial neural vision.

Hardware‐based artificial neural vision systems emulate visual functions at the device level by constructing physical synaptic arrays to mimic neural connections and signal processing. In contrast, software‐based artificial neural vision systems build neural networks by abstractly modeling synaptic structures and functions, using algorithms to simulate information transmission and synaptic weight modulation.

In artificial neural networks (ANNs), synaptic functions are typically represented by connection weights. Neuron nodes, together with these weighted connections, form multilayer structures that transform human‐perceived data into features that computers can process. To accommodate different types of data and tasks, researchers have developed a variety of neural network models and architectures. Common examples include convolutional neural networks (CNNs), feedforward neural networks (FNNs), recurrent neural networks (RNNs), and graph neural networks (GNNs). CNNs are used for image feature extraction, FNNs for task classification, recognition, and optimization, RNNs for extracting features from textual data, and GNNs for structured feature extraction‐such as modeling complex relationships and dependencies between nodes in molecular structures, transportation networks, social relationships, and more. This paper primarily focuses on CNNs and FNNs, which are used in artificial visual neural networks for processing image data.

#### 3.1.1 Convolutional Neural Network

Driven by the rapid progress of deep learning, CNNs have emerged as indispensable tools for image recognition and classification. By mimicking the hierarchical structure of the biological visual cortex, CNNs enable efficient extraction and identification of image features. However, conventional CNNs operate under the Von Neumann architecture, which suffers from data transfer bottlenecks and high energy consumption. To overcome these limitations, researchers have begun exploring the integration of CNNs with optoelectronic synapse arrays. A key characteristic of convolutional neural networks is the use of convolutional kernels, which are employed to extract image features.

**Figure 7:**
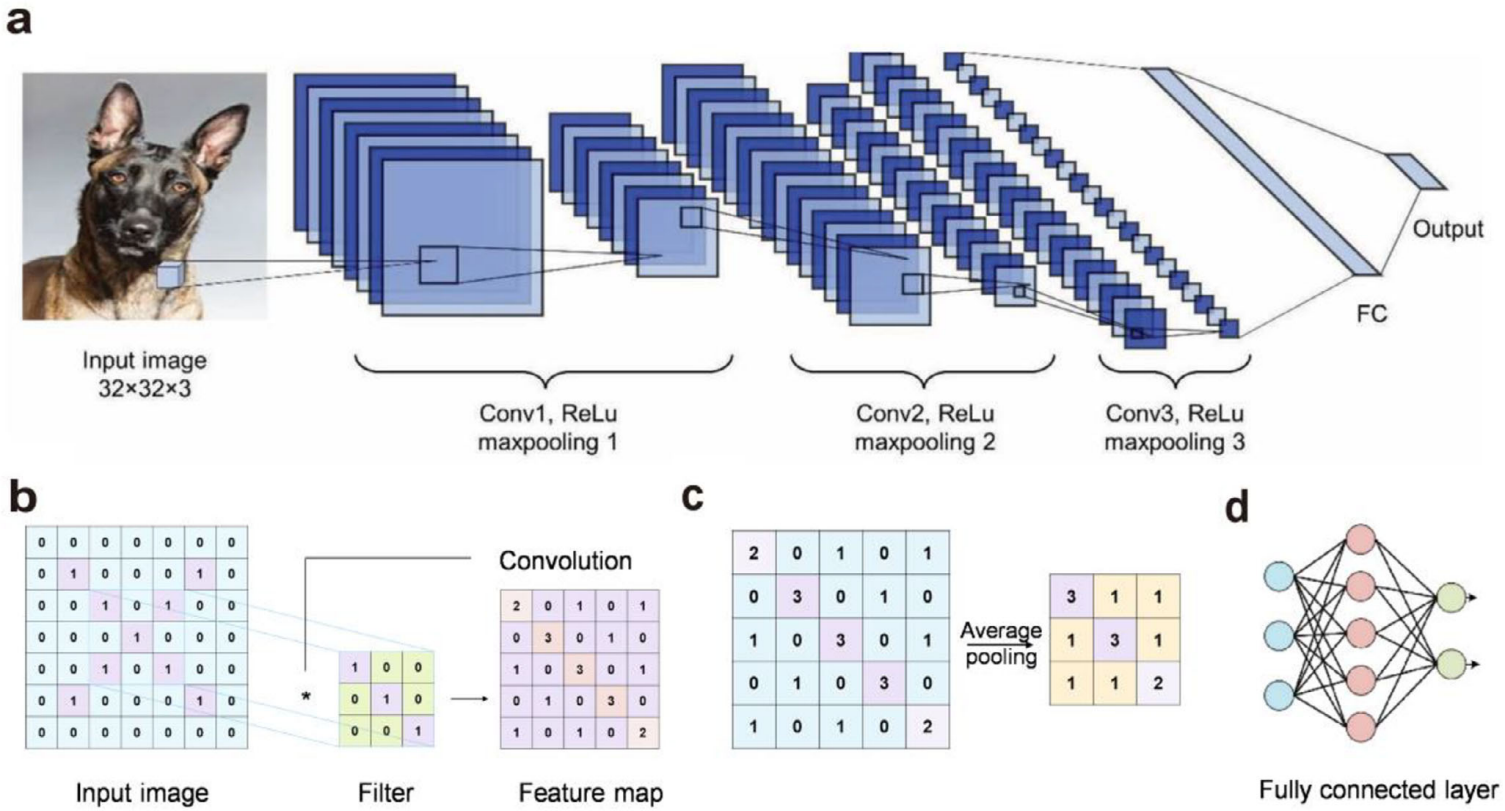
Schematic of CNN architecture for image recognition. a) Schematic diagram of image recognition using a convolutional neural network framework.^[^ ^116^ ^]^ b) Convolutional kernel extraction process for image features.^[^ ^117^ ^]^ c) Spatial downsampling via pooling.^[^ ^117^ ^]^ d) Classification decision by a fully connected layer.^[^ ^117^ ^]^

Convolutional neural networks are designed to mimic the way the brain first recognizes local image features and then progressively integrates them to form a holistic perception. As illustrated in **Figure** **7**a, a typical CNN consists of convolutional layers, pooling layers, and fully connected layers.^[^ ^115^ ^]^ The convolutional layers employ kernels to extract features, the pooling layers reduce the dimensionality of the image data to improve recognition efficiency, and the fully connected layers are responsible for the final classification and decision‐making.

For the convolutional layer, taking the 7 × 7 digit image shown in the middle left of Figure 7b as an example, a 3 × 3 convolutional kernel is set to extract features from the fifth column. For the diagonal feature extending from the top‐left to the bottom‐right, the convolutional kernel is also configured accordingly. The kernel is then multiplied by the corresponding region in the image, and the results are summed. The output values are placed into another matrix. The convolutional kernel is then slid sequentially across the image, moving one pixel at a time with overlapping regions, until the entire output matrix is filled. Higher values on the right side of the matrix indicate a stronger correspondence to the original image features. For the bottom‐left diagonal feature, a convolutional kernel designed to detect that specific diagonal pattern is selected, and the same operation is performed.

For the pooling layer, due to the large size of input images and the resulting computational cost, pooling is applied to reduce the dimensionality of the data. As illustrated in Figure 7c, the operation retains either the maximum or average value among four neighboring pixels, and this calculation is performed sequentially across the image. In this case, the pooling window moves without overlap, shifting two pixels at a time both horizontally and vertically. It can be observed that, even after pooling, the essential features of the original image are still preserved.

The purpose of the fully connected layer is to enable the computer to recognize the processed image. As shown in the upper part of Figure 7d, two 3 × 3 matrices are flattened into one‐dimensional arrays and concatenated into a single vector. While this vector is not interpretable to the human eye, it is recognizable by the computer. This is because, prior to recognition, the computer is trained using a dataset of sample images. During training, the computer learns and stores a feature vector for each specific image. When recognizing a new image, the computer generates a feature vector from the input and compares it with the stored vectors from training. Object recognition is then achieved based on the similarity between these vectors.

#### 3.1.2 Feedforward Neural Network

The final part of a convolutional neural network is often combined with a fully connected neural network (FCNN) to perform image recognition and classification. Both the FCNN and CNN are considered subsets of FNNs. The fully connected neural network (FCNN) is similar to a general feedforward neural network (FNN). The primary difference lies in the connectivity pattern. In an FCNN, each neuron in one layer connects to every neuron in the next layer. In contrast, an FNN may employ fully connected, locally connected, or sparsely connected schemes. Below, we present a detailed introduction to the design of feedforward neural networks.

**Figure 8:**
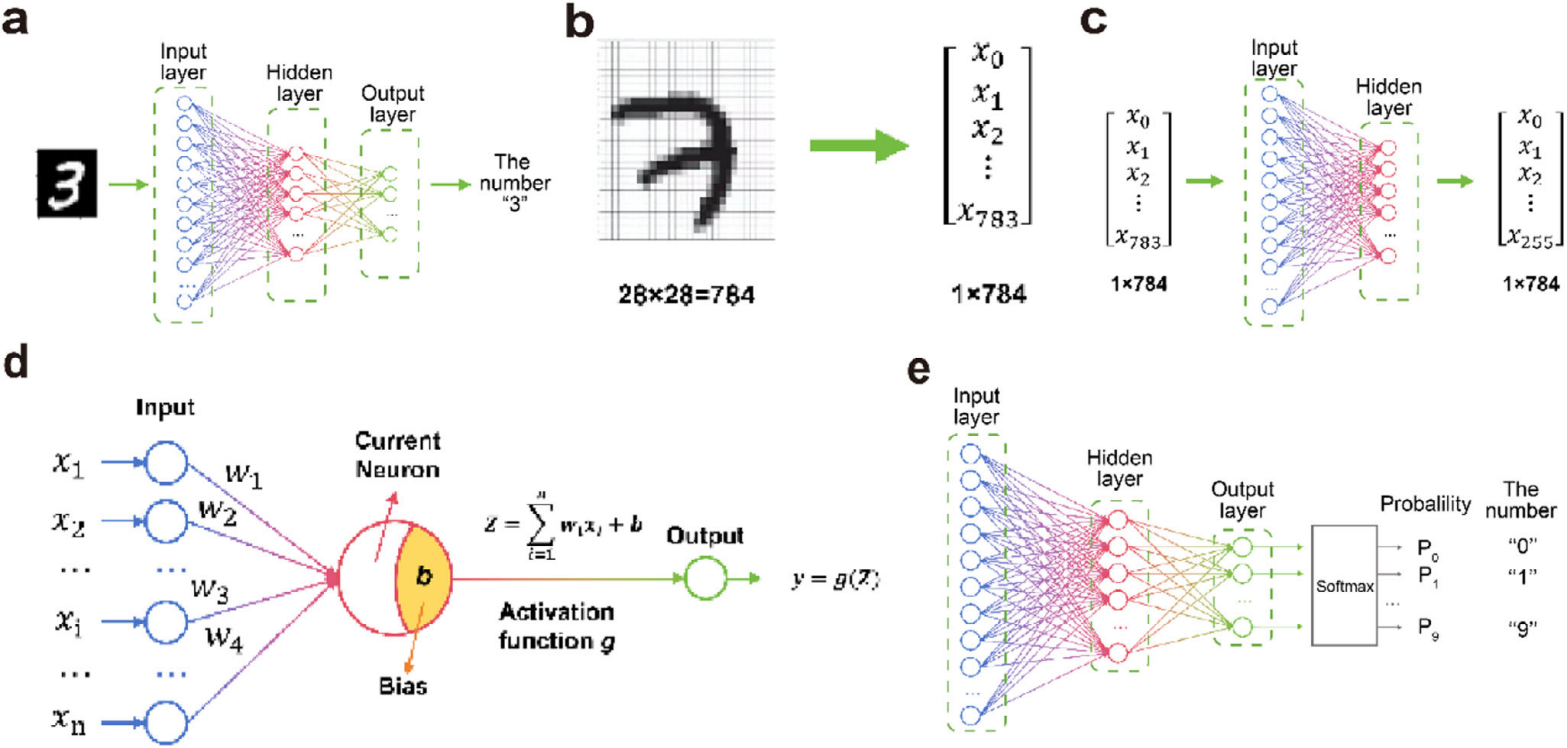
Feedforward neural network architecture and workflow. a) Schematic diagram of an artificial neural network performing classification tasks.^[^ ^118^ ^]^ b) The process of flattening an image into a one‐dimensional vector.^[^ ^118^ ^]^ c) Signal transmission from input layer neurons to hidden layer neurons.^[^ ^119^ ^]^ d) A typical M‐P neuron model used for hidden layer computations.^[^ ^120^ ^]^ e) The process of signal propagation from the hidden layer to the output layer neurons and the classification decision function.^[^ ^121^ ^]^

As shown in **Figure** **8**a, a 3 × 3 neural network takes a digit image as input and, through computation within the network, classifies the digit as “3”, thereby achieving digit image recognition. For grayscale digit images with a resolution of 28 × 28 pixels, there are 784 data points in total. These data must first be flattened into a 1 × 784 vector (Figure 8b), which is then fed into the neural network for processing.

A neural network typically consists of three fundamental layers: an input layer, one or more hidden layers, and an output layer. The input layer receives a 784‐dimensional image vector, with each element corresponding to a single pixel. Therefore, the input layer contains 784 neurons. Each connection between neurons is associated with a weight, and each neuron also has an associated bias term.

The hidden layer is responsible for feature extraction and transformation, processing the input feature vector into a higher‐level representation. As illustrated in Figure 8c, due to the simplicity of the image, the number of neurons in the hidden layer can be set to 256. Consequently, a linear layer of size 784 × 256 is formed between the input and hidden layers, representing the weight matrix. This layer transforms the 784‐dimensional input vector into a 256‐dimensional output vector, which is then propagated forward to the output layer.

The data processing in the hidden layer follows the typical McCulloch‐Pitts (M‐P) neuron model, in which the core operations are weighted summation followed by activation through a nonlinear function. As shown in Figure 8d, it is important to note that each neuron in the preceding layer sends information to every neuron in the subsequent layer, forming a fully connected structure. Each neuron in the hidden layer receives input signals from n neurons (in this case, 784 neurons) in the input layer, denoted as X_1_ through X_n_. These inputs are transmitted through corresponding weights W_1_ to W_n_, which connect the input neurons to the hidden neuron. Inside the hidden neuron, each input X_i_ is multiplied by its associated weight X_i_, and the results are summed. A bias term b is then added to this weighted sum, yielding the intermediate output Z, as defined in Equation (14). The value Z is then processed by an activation function, such as the Sigmoid function, to produce the final output y. An activation function is a mathematical function applied to a neuron that maps the input data to an output value. Commonly used activation functions include the Sigmoid function, the hyperbolic tangent (tanh) function, and the Rectified Linear Unit (ReLU) function.

(14) $$Z=\sum_{i=1}^{n}\left(W_{i}X_{i}+b\right)$$

Since the goal of the output layer is to classify digit images into one of the 10 possible categories (0 through 9), the output layer is designed with 10 neurons, each corresponding to one digit. The 256‐dimensional feature vector produced by the hidden layer is further processed through a linear transformation in the output layer, resulting in a 10‐dimensional output vector. This vector represents the prediction scores for each of the 10 digits. To obtain the predicted probabilities, the output vector is passed through a softmax layer. The softmax function converts the 10‐dimensional score vector into 10 probability values, P_0_ through P_9_, where each probability indicates the likelihood that the input image corresponds to a specific digit. The data processing in the output layer is similar to that in the hidden layer: it first involves a weighted summation followed by activation through a nonlinear function, and is then further processed by the softmax function (Figure 8e).

In a neural network, each connection is associated with a weight that determines the strength of signal transmission within the network. Additionally, each neuron has a bias term that defines its activation threshold. Training the network involves adjusting these weights and biases. During the training process, input images are fed into the network, and the resulting outputs are compared with the ground truth labels. The discrepancies are then used to update the weights and biases through the backpropagation algorithm.

#### 3.1.3 Neural‐Network Loading and Operating Mechanisms in Optoelectronic Synaptic Arrays

Neuromorphic vision systems can perform image recognition, classification, and processing—namely, machine vision. Machine vision is more than simple light detection; it relies on synaptic functions for recognition and classification, which in turn requires the integration of synaptic arrays with neural networks.

**Figure 9:**
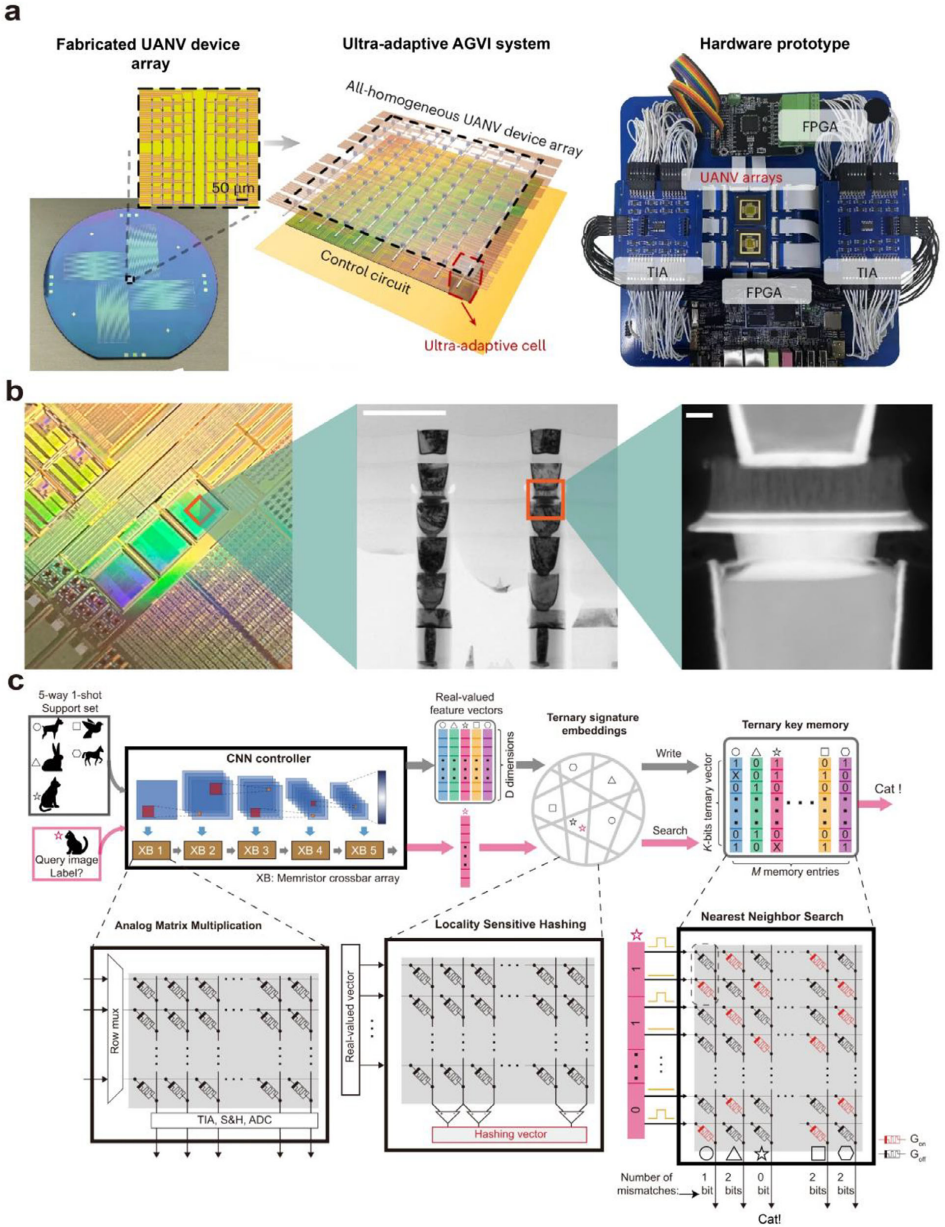
a) Optical micrograph of the optoelectronic synaptic array, the stacked architecture, and the prototype hardware system (including the optoelectronic synaptic array and the board‐level dynamic control circuit).^[^ ^122^ ^]^ b) Optical photograph of the memristor chip, cross‐sectional TEM image of the memristor array, and TEM image of a single memristor cell.^[^ ^123^ ^]^ c) Schematic of the memory‐augmented neural network (MANN) architecture based on crossbars for recognition and classification tasks.^[^ ^124^ ^]^

An optoelectronic synapse is essentially a photosensitive memristor or synaptic transistor whose conductance (weight) can be modulated by either electrical or optical pulses. These basic units are arranged into a crossbar array (**Figure** **9**a), with each row–column intersection forming one optoelectronic synapse.^[^ ^124^ ^]^ Figure 9b shows a TEM image of a single memristive cell.

#### 3.1.4 Training Process

The operation of a synaptic array relies on the neural network—the weight matrix—loaded into it. Rather than copying a network model into memory, loading here means physically changing the conductance of every synapse through training. Training can be electrical, optical, or both, and is usually guided by the STDP rule (Section 2.3.10) to adjust conductance and hence weight.

Optical training: the image or dynamic scene to be learned is projected onto the array by an optical system (e.g., lenses). Different sites receive different light intensities, changing the local conductance and thus the weights in parallel. Light, therefore, writes the weight map directly. Because pure‐optical training still lacks the precision and controllability of electrical methods, it is normally combined with electrical tuning.

Electrical training: specific voltage or current pulses are applied across the device terminals to reversibly modulate its conductance. Positive or negative pulses increase or decrease the conductance in an STDP‐like fashion. Amplitude, width, and frequency can be controlled precisely, giving high accuracy and repeatability.

Hybrid training (the most widely used): a practical optoelectronic system combines both steps:

1. Optical phase: the image is projected, imposing an initial, image‐related conductance pattern in parallel—this performs “perception” and coarse encoding.
2. Electrical phase: the array produces an output spike train that is compared with the desired output to generate an error signal. This error is converted into a sequence of fine electrical pulses delivered to the relevant synapses. Pulses that strengthen (or weaken) the conductance are applied according to whether the synapse contributes positively or negatively to correct recognition, iteratively refining and fixing the optically set weights until the network performs the task accurately.

#### 3.1.5 Read and Inference Process

After training, the stored pattern is read out by matrix multiplication. A read voltage *V*_read_ is applied to a row (or column); by Ohm's law, the current through each synapse is *I* = *V*_read_ × *G*, and since *G* has been set by training, *I* carries the learned light‐intensity information.

Inference proceeds similarly. Once the weight matrix *W* is loaded, each element *V*_i_ of the input feature vector is converted to an analog voltage and applied to the corresponding row. The current through each memristor is *I*_ij_ = *V*_i_ × *G*_ij_; by Kirchhoff's current law, the total current on column bus j is *I*_j_ = Σ(*V*_i_ × *G*_ij_). Each column's current *I*_j_ is fed to an ADC to produce a digital value or spike train for the next layer, completing the inference.

#### 3.1.6 Neural‐Network Loading

Neural‐network loading into a synaptic array can be done off‐chip (train on GPU/CPU with back‐propagation, then map the weights) or on‐chip (train in place with STDP‐like rules that exploit physical laws). Loading a large CNN or FNN directly is impractical; hierarchical and modular approaches are therefore used.

Software step: the big network is split into small, reusable functional blocks—e.g., one convolutional layer, one fully‐connected layer.

Hardware step: each block is assigned to a small opto‐electronic synaptic sub‐array, and the sub‐arrays are connected by on‐chip routing. The same physical array can also be time‐shared: a controller rapidly reloads different layer weights (conductance maps) so the array sequentially performs the role of every layer in a pipeline. Figure 9c illustrates three typical tasks:^[^ ^124^ ^]^

##### Feature Extraction

The trained convolutional (or encoder) weights are mapped to conductances. The input image is optically projected onto this array; the outgoing current pulses are not final labels but abstract features. These feature vectors are captured and stored or forwarded.

##### Hash Computation and Similarity Search

A special fully‐connected “hash layer” is trained and mapped to a small array whose activation is the sign function (+1/–1, i.e., bit 0/1). Feeding the feature vector through this array and thresholding the column currents yields a binary hash code; similar images produce similar codes.

##### Content Retrieval

A CNN feature extractor plus a hash layer is first trained on a GPU. Their weights are mapped to two separate arrays. Every image of a large dataset is optically projected, producing a hash code that is stored in conventional memory with an index. At query time, the user's image is projected, its hash code is generated, and a Hamming‐distance search instantly returns the most similar entries.

#### 3.1.7 Role of the Hardware System

The right‐hand side of Figure 9a shows the hardware system composed of the synaptic array and its peripheral circuits, which form the basis for training and reading out the trained results. The training and readout procedures integrated with this hardware are as follows:

1. A neural network is first trained on a GPU/CPU using a conventional algorithm (e.g., back‐propagation) to obtain target weights.
2. A digital controller (FPGA) converts the target weights into digital codes, which a DAC turns into analog voltage pulses capable of changing memristor conductance. After amplification by write drivers, these pulses are routed through a switch matrix/multiplexer to the selected row and column, tuning the synaptic weight—this is the training step.
3. To read, a small, non‐destructive read voltage is applied to the target cell, producing a minute current proportional to its conductance. This current is fed to a trans‐impedance amplifier (TIA) that linearly converts it to a voltage, which an ADC then digitizes, yielding the cell's present conductance state.

### 3.2 The Fundamental Functions of the Visual System

#### 3.2.1 Imaging Function

One of the most fundamental functions of the visual system is the acquisition and processing of external image information, commonly referred to as imaging. Imaging serves not only as the basis for biological organisms to perceive their surroundings and generate behavioral responses, but also as the prerequisite for artificial vision systems to achieve information perception, target recognition, and scene understanding. Although conventional imaging devices have become relatively mature, the pursuit of higher sensitivity, lower power consumption, and more versatile functional integration has brought emerging advantages and significant development potential to imaging technologies based on novel low‐dimensional materials.

**Figure 10:**
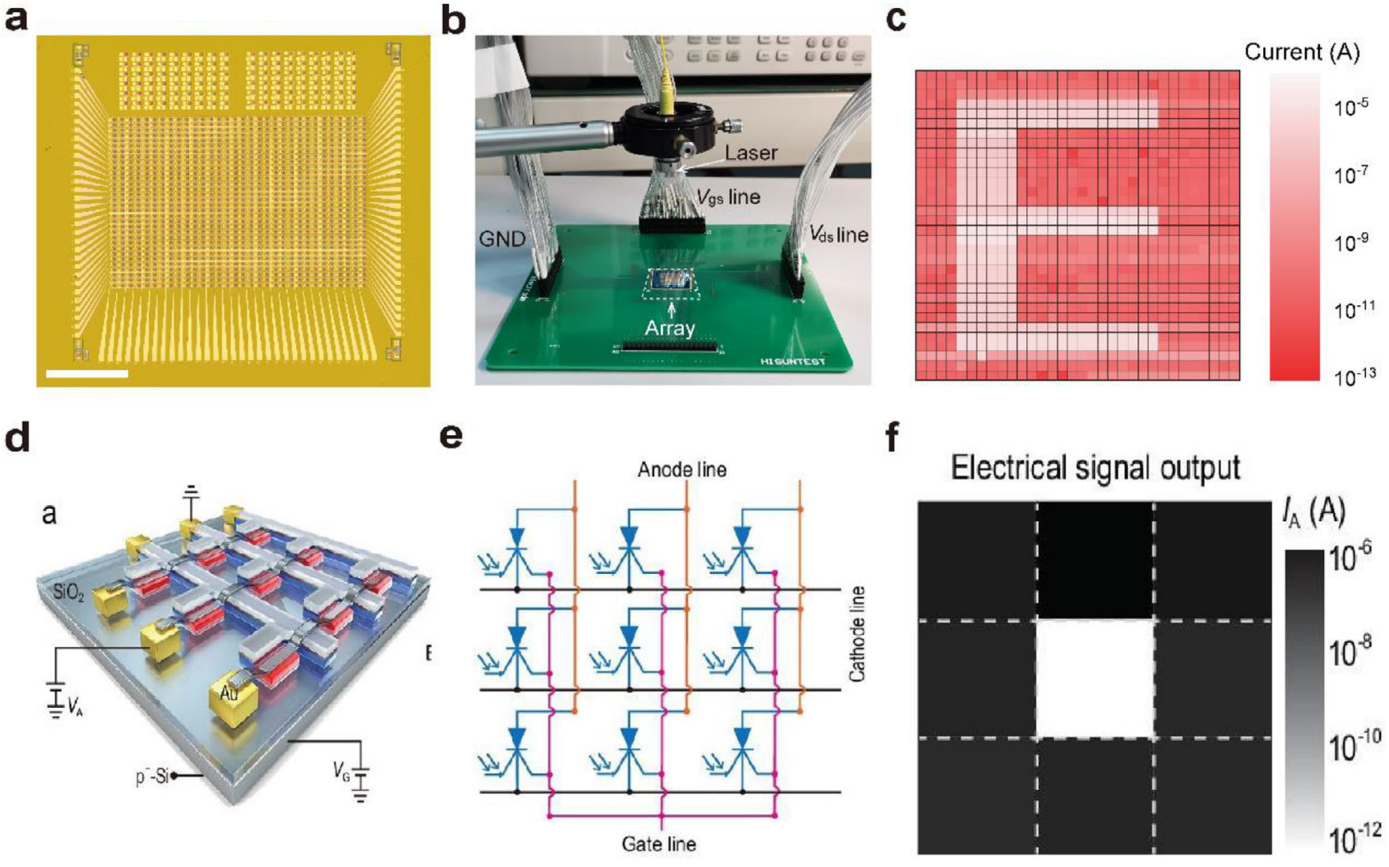
Imaging function of the vision system. a) Optical image of a 1024‐pixel image sensor array (scale bar: 3.25 mm).^[^ ^125^ ^]^ b) Schematic diagram of optoelectronic imaging array test.^[^ ^125^ ^]^ c) The clear “E” image detected by the array.^[^ ^125^ ^]^ d) Schematic of a 3 × 3 array without selectors.^[^ ^126^ ^]^ e) Equivalent circuit.^[^ ^126^ ^]^ f) Array imaging demonstration.^[^ ^126^ ^]^

Sun et al. integrated two‐dimensional MXene materials with conventional silicon‐based substrates to construct a Schottky junction photodetector, which exhibited an impressive detectivity of up to 7.73 × 10^14^ Jones and an on/off current ratio of 6.22 × 10⁶. Building upon this, the researchers further integrated the high‐performance photodetector with a carbon nanotube transistor to form a 1‐transistor‐1‐photodetector (1T1P) pixel architecture. A high‐resolution photodetection array comprising 1024 pixels was successfully demonstrated based on this configuration (**Figure** **10**a). Meanwhile, the team designed a compatible imaging detection system and demonstrated effective image acquisition using the MXene‐based photodetector array (Figure 10b,c). Although this work demonstrated the array imaging capability enabled by novel materials, the overall performance still requires further improvement when compared to conventional imaging arrays.

To reduce the structural complexity of imaging arrays, Sun et al. developed a novel phototransistor array based on low‐dimensional materials such as MoS_2_ and graphene (Figure 10d). The phototransistors exhibited a high responsivity of up to 10⁸ and a photoconductive on/off ratio exceeding 10⁶. In terms of device architecture, the array demonstrated a simplified circuit design by eliminating the need for externally integrated gating components (Figure 10e). Figure 10f presents the imaging demonstration of the photonic memory array constructed without gating components. The results indicate that the device retains excellent imaging resolution even in the absence of dedicated gate elements. This “gate‐less” circuit architecture holds significant potential for optoelectronic systems, providing a novel approach and technological pathway toward the realization of highly integrated, low‐power intelligent vision systems.

**Figure 11:**
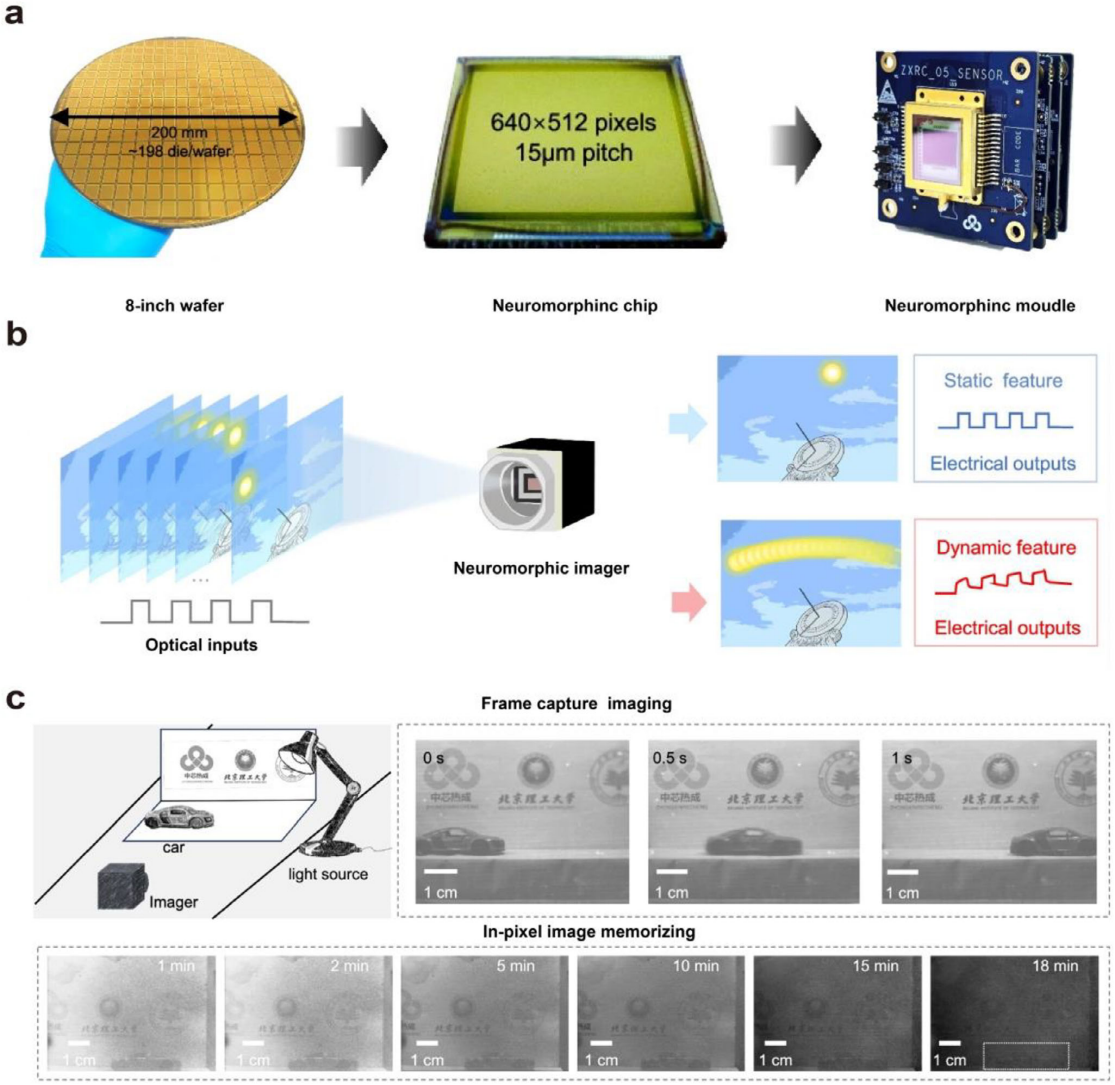
Organic neuromorphic imager based on a PTB7‐Th:PZ1 bulk‐heterojunction that enables dual‐modal imaging. a) Photographs of the wafer‐scale neuromorphic chip, the neuromorphic CMOS sensor, and the assembled imaging module.^[^ ^127^ ^]^ b) Schematic comparison between the standard imaging mode and the synaptic (memory) mode of the imager.^[^ ^127^ ^]^ c) Top: consecutive snapshots of a moving car acquired in standard mode at different time points; bottom: car images captured in synaptic mode after the illumination source is switched off, showing the persistence of the stored pattern at each corresponding moment.^[^ ^127^ ^]^

Talanti et al.^[^ ^127^ ^]^ presented a CMOS‐integrated organic neuromorphic imager that offers two distinct operational modes: standard imaging and synaptic imaging (**Figure** **11**a). The active layer is a PTB7‐Th:PZ1 bulk heterojunction whose memory retention can be tuned at will by adjusting the acceptor (PZ1) content. As illustrated in Figure 11b, a high PZ1 fraction (50 wt%) accelerates charge recombination, yielding negligible memory and enabling high‐speed standard imaging. Conversely, a low PZ1 content (1 wt%) introduces abundant interfacial traps that prolong electron retention, giving rise to long‐lasting memory suitable for synaptic operation. By integrating the two modes of synaptic transistors into a 640 × 512 pixel chip and selectively reading signals from different regions, standard and synaptic mode images can be obtained separately (Figure 11c).

#### 3.2.2 Preprocessing Function

Image preprocessing functions include enhancement of image quality and noise filtering. Performing image preprocessing at the sensor level can amplify salient image features while suppressing irrelevant information, thereby improving the performance of subsequent image processing tasks such as object recognition and motion trajectory extraction. The clarity of the image can be achieved by the brightness and color difference between the target subject and the background, respectively.^[^ ^128^ ^]^

The implementation of image preprocessing functionality can be approached from the perspective of material properties. Given that the target subject typically exhibits a significant luminance contrast with the background, it is essential to identify a material that exhibits both positive photoconductive memory (PPM) and negative photoconductive memory (NPM) effects. Specifically, under strong illumination, the application of light stimulation enhances the device's conductivity, resulting in an increased postsynaptic current. In contrast, under weak illumination, light stimulation induces an inhibitory effect, reducing the conductivity and suppressing the postsynaptic current.

**Figure 12:**
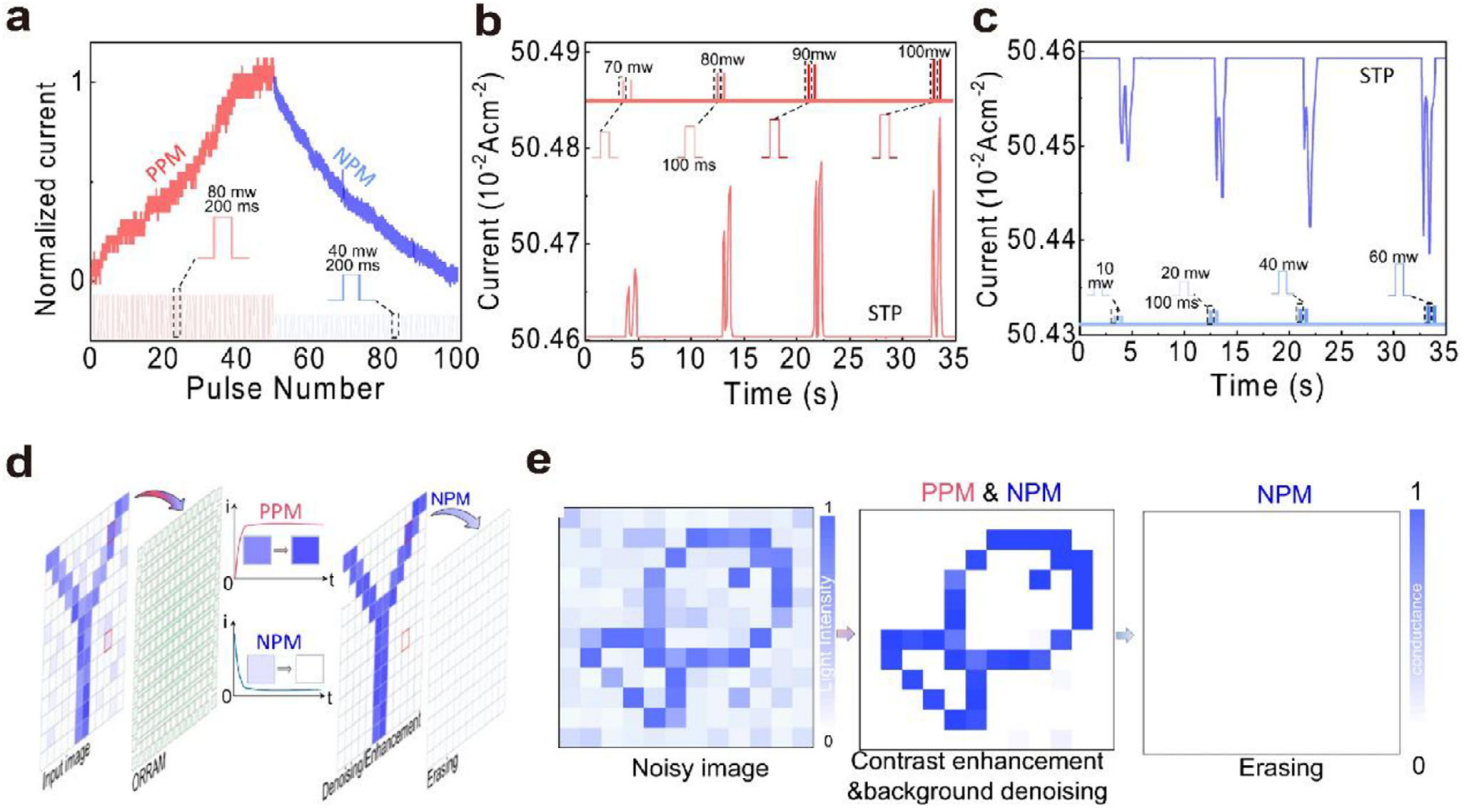
Contrast enhancement function of artificial neural visual array. a) Optically induced potentiation and depression processes under the continuous 80 mW, 200 ms light pulses and 40 mW, 200 ms light pulses, respectively.^[^ ^20^ ^]^ b,c) Positive photoconductive memory and negative photoconductive memory STP triggered by pair pulses with light intensities ranging from 10 to 60 mW and 70 to 100 mW, respectively.^[^ ^20^ ^]^ d) Demonstration of the image contrast enhancement and denoising through the simultaneous NMP and PPM effects, and image erasing through negative photoconductive memory effects.^[^ ^20^ ^]^ e) A “fish” image was sensed, denoised, and enhanced in situ a 12×12 MSFP‐based multimodal memory crossbar array. The memorised and preprocessing image in the array can be further erased by light illumination.^[^ ^20^ ^]^

Zhou et al. investigated a class of materials exhibiting such unique photoresponsive behavior, namely modified silk fibroin proteins (MSFPs).^[^ ^20^ ^]^ They extracted silk fibroin from natural silk as the base material and subsequently mixed it with specific chemical compounds, such as polyglycerol‐3 (Pg‐3) and 5,6‐dihydroxyindole (5‐6‐DHI). Through chemical reactions, these compounds formed strong hydrogen bonds with the silk fibroin, thereby altering its secondary structure and resulting in the formation of MSFP. The material's response to light pulses is illustrated in **Figure** **12**a. Under continuous light stimulation at 80 mW for 200 ms, the device exhibited a photo‐induced enhancement effect, whereas under 40 mW for 200 ms, it showed a photo‐induced suppression effect. More detailed testing revealed that within the higher intensity range of 70–100 mW, the device consistently demonstrated PPM behavior (Figure 12b), while under lower light intensities ranging from 10 to 60 mW, it exhibited NPM behavior (Figure 12c). The authors further observed that the light intensity threshold ranges for PPM and NPM could be modulated by altering the composition of the MSFP film as well as the operating temperature. Li et al.^[^ ^129^ ^]^ demonstrated full‐optical, bidirectional photoconductance in a single device: ultraviolet illumination evokes negative photoconductance (NPC) while red light triggers positive photoconductance (PPC), offering an intrinsic hardware platform for on‐sensor image pre‐processing. Exploiting this wavelength‐selective behavior, a denoising protocol can be envisaged: the entire scene is first scanned with red light to globally “write” the image (signal plus noise), and the noisy pixels are subsequently “erased” by local UV exposure, leaving only the high‐intensity signal regions intact. Figure 12d,e illustrates the effectiveness of the synaptic device array, fabricated from this material, in image preprocessing tasks, using the letter “Y” and a fish‐shaped pattern as input examples. When the image is applied to the synaptic array, pixels corresponding to the brighter regions of the image, such as the “Y” and the fish‐are enhanced due to the **PPM** effect. In contrast, background pixels with lower luminance are suppressed via the **NPM** effect. This differential response amplifies the signal contrast between the target and background regions, thereby enhancing image contrast and smoothing out background noise.

**Figure 13:**
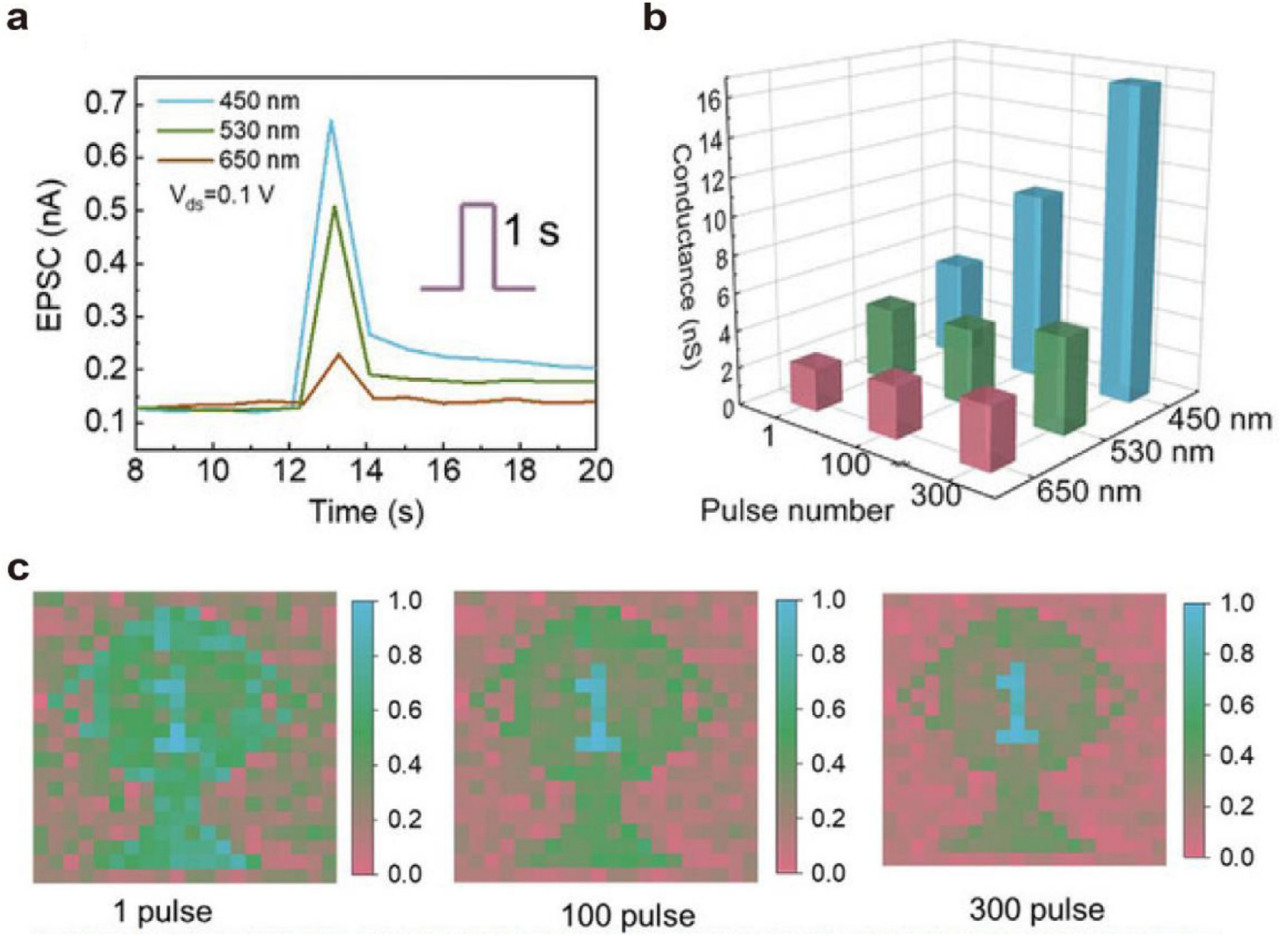
Color contrast enhancement function of an artificial neural visual array. a) The time‐dependent variation of EPSC is examined under a single optical pulse with various light wavelengths. The optical pulse possesses a power density of 5.3 µW cm^−2^ and a duration lasting 1 second.^[^ ^56^ ^]^ b) 3D histogram image of the conductance states as a function of pulse number under three wavelengths.^[^ ^56^ ^]^ c) The information extraction and noise reduction processes with optical spike number enhancement.^[^ ^56^ ^]^

In addition to luminance differences, the target object in an image typically exhibits noticeable color contrast with the background. If a material can be engineered to possess stronger absorption for specific wavelengths of light, it would enable targeted image denoising and other preprocessing strategies. Dang et al. discovered that the 2D material rhenium disulfide (ReS_2_) exhibits distinct wavelength‐dependent optical absorption characteristics.^[^ ^56^ ^]^ Specifically, ReS_2_ demonstrates strong absorption in the blue‐light region (e.g., 450 nm), while its absorption in the red‐light region (e.g., 650 nm) is comparatively weaker. They also fabricated synaptic devices based on P(VDF‐TrFE)/ReS_2_ and investigated their photoresponse under different light wavelengths. As shown in **Figure** **13**a, the excitatory postsynaptic current (EPSC) of the device gradually increases as the illumination wavelength decreases from 650 nm to 450 nm, indicating a higher sensitivity to blue light. Furthermore, through selective and repeated stimulation with blue light, the synaptic properties of the device can be utilized to further enhance its sensitivity. As illustrated in Figure 13b, the device conductivity under 450 nm blue‐light illumination increases significantly with the number of light pulses applied. Figure 13c illustrates the image preprocessing performance of a synaptic array based on the aforementioned material. In the input image, the background, trophy outline, and numbers on the trophy correspond to red, green, and blue colors, respectively. At the initial stage (after a single light pulse), the photoresponse is relatively weak, making the blue features of the numbers indistinct and difficult to differentiate from the background and the trophy outline. As the number of light pulses increases, the conductivity in the blue‐light‐sensitive regions rises significantly, and the blue digits gradually become more distinguishable. When the number of light pulses reaches 300, the blue digits on the trophy become most prominent, while the signals corresponding to the red background and green trophy outline are relatively suppressed. This enables the extraction of key blue‐colored information from a complex multicolor image while simultaneously reducing noise interference caused by other color channels.

#### 3.2.3 Adaptive Function

Under bright illumination conditions, human vision primarily relies on cone cells to perceive light—a process known as photopic adaptation.^[^ ^13^ ^]^ In low‐light environments, rod cells become the dominant photoreceptors, enabling scotopic adaptation. During photopic adaptation, in order to distinguish varying levels of brightness under intense light conditions, the visual system must elevate its visual threshold. Optoelectronic synaptic devices can achieve adaptive functionality either through gate voltage modulation or by employing specially designed materials and structures that enable the device to autonomously adjust its sensitivity in response to varying light intensities.

**Figure 14:**
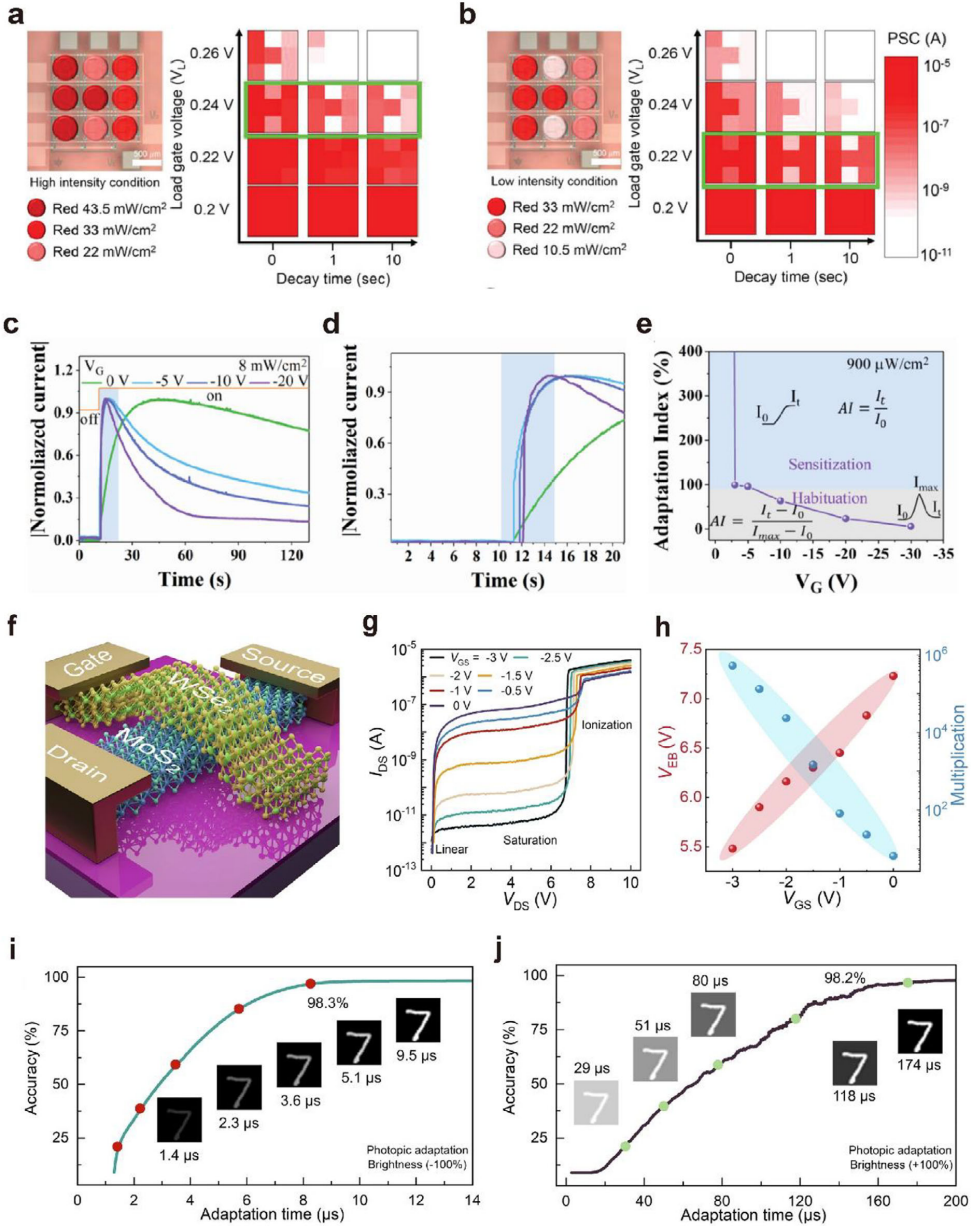
Visual adaptive functions of different types of artificial vision devices. a) Adaptive behavior of optoelectronic synapse arrays in a strong light environment under bias voltage modulation.^[^ ^13^ ^]^ b) Adaptive behavior of optoelectronic synapse arrays in low‐light environment under bias voltage modulation.^[^ ^13^ ^]^ c) The compared time‐dependent current under different gate voltages of 0, −5, −10, and −20 V at a continuous light illumination of 8 mW cm^−2.[^ ^130^ ^]^ d) The magnified sensitization process corresponding to the area marked by the blue shaded area in (c).^[^ ^130^ ^]^ e) Extracted adaptation index at various *V*_G_ values under the continuous illumination of 900 µW cm^−2^ after the same duration time of 120 s.^[^ ^130^ ^]^ f) Optoelectronic synapse devices based on MoS_2_/WSe_2_ 2D materials.^[^ ^131^ ^]^ g) Schematic of the avalanche effect in a synaptic device.^[^ ^131^ ^]^ h) Plot of electrical breakdown voltage (*V*_EB_) vs gate voltage (*V*_GS_)^[^ ^131^ ^]^ i) Image recognition ability of a synaptic device under dim light conditions.^[^ ^133^ ^]^ j) Image recognition ability of a synaptic device under bright light conditions.^[^ ^131^ ^]^

In optoelectronic synaptic devices, gate voltage modulation is one of the most typical approaches to achieving adaptive functionality. By adjusting the load bias voltage *V*_L_ through gate control, the visual threshold can be modulated to realize adaptation to both bright and dim lighting conditions. A photovoltage divider composed of a CdSe photodetector and an a‐IGZO synaptic transistor serves as an analog to the biological retina, while an ion synaptic transistor is used to mimic the biological optic nerve.^[^ ^13^ ^]^ When suddenly exposed to a bright light environment, as shown in **Figure** **14**a, with light intensities ranging from 10.5 to 33 mW/cm^2^, it is evident that the current transmitted to the ion transistor is generally elevated, causing the signal to saturate easily. Increasing the bias voltage *V*_L_ across the photovoltage divider enables a distinct representation of the letter “H”. Conversely, under low‐light conditions, as illustrated in Figure 14b, decreasing the bias voltage enhances the photovoltage divider's sensitivity to weak illumination. This increases the differential current signal transmitted to the ion synaptic transistor, resulting in a clearer representation of the letter “H”.

The specific influence of different gate voltages on the postsynaptic current is shown in Figures 14c,d,^[^ ^130^ ^]^ Under constant illumination intensity, an increase in gate voltage accelerates the return of the postsynaptic current to its equilibrium state. The response curve of the postsynaptic current under high light intensity gradually returns to that under normal lighting conditions, reflecting the biological adaptation behavior to strong illumination. Both the sensitivity and adaptation speed of the postsynaptic current increase with higher gate voltage. The adaptation index (AI), shown in Figure 14e, is used to quantify the light adaptation capability of the phototransistor, defined as the ratio between the initial current (in the dark) and the photocurrent after a period of illumination. When the gate voltage is below 3 V, the AI is significantly greater than 1, indicating a normal visual adaptation response under low‐light conditions. At higher gate voltages (*V*_G_ > 3 V), the AI drops below 1, suggesting that elevated gate voltage weakens the influence of increased carrier density on visual adaptation.

Adaptive functionality can also be realized by fabricating devices that autonomously adjust their sensitivity in response to varying light intensities. A biomimetic two‐dimensional transistor based on MoS_2_/WSe_2_ materials (Figure 14f) achieves this function by spontaneously switching between avalanche effect and photoconductive effect.^[^ ^131^ ^]^ Specifically, under strong illumination, the device operates predominantly under the photoconductive effect, resulting in reduced responsivity and sensitivity, analogous to the low‐sensitivity response of cone cells in bright light. Under weak illumination, the device primarily exhibits avalanche characteristics. As shown in Figure 14g, when the *V*_DS_ exceeds a specific *V*_EB_, the *I*_DS_ increases sharply, exhibiting avalanche behavior characterized by high responsivity and sensitivity. The avalanche effect refers to the phenomenon in semiconductor devices where, under a sufficiently strong electric field, charge carriers (electrons or holes) gain enough energy to ionize atoms through impact ionization, generating a large number of new carriers and thereby causing a sudden surge in current.

The ability of the MoS_2_/WSe_2_ heterojunction device to switch between avalanche effect and photoconductive effect depending on illumination intensity originates from the difference in work functions between MoS_2_ and WSe_2_, which creates a built‐in electric field at the interface. Under low‐light conditions, the concentration of photogenerated carriers is low, making the acceleration and ionization of carriers by the built‐in electric field more pronounced. This facilitates the occurrence of the avalanche effect, resulting in high responsivity and sensitivity of the device. Under strong illumination conditions, the concentration of photogenerated carriers increases, and the built‐in electric field is weakened due to modulation by the photovoltage, thereby suppressing the avalanche effect and causing the device to predominantly exhibit the photoconductive effect. Figure 14h shows the variation of the *V*_EB_ with *V*_GS_. As *V*_GS_ decreases, *V*_EB_ also decreases, indicating that by adjusting the gate voltage, the depletion region and vertical electric field can be effectively modulated to control the avalanche characteristics of the device.

Figure 14i,j illustrates the image recognition capabilities of the system under different illumination conditions. When the ambient lighting transitions from bright to dim, the system rapidly adjusts its visual adaptation mechanism to accommodate the low‐light environment. As shown in Figure 14i, the recognition accuracy rises to 98.3% within 9.5 microseconds, demonstrating the system's ability to quickly and accurately identify image features. Similarly, when the ambient lighting shifts from dim to bright, the system promptly adapts its visual processing to suit the high‐light environment. Figure 14j shows that the recognition accuracy rapidly increases to 98.2% within 174 µs, indicating the system's fast and precise image recognition under strong illumination.

#### 3.2.4 Motion Detection Function

The motion detection capability of neuromorphic visual sensors enables the system to adapt to dynamically changing environments. For instance, in autonomous vehicles, it allows real‐time responses to the movement of other vehicles, pedestrians, and animals. By responding only to the parts of the scene that have changed, data‐acquisition redundancy is slashed, cutting storage and processing demands while boosting system speed and security.^[^ ^132^ ^]^ Motion image detection can be achieved either through specialized circuit unit designs or via the intrinsic relaxation characteristics of the synapses themselves.

**Figure 15:**
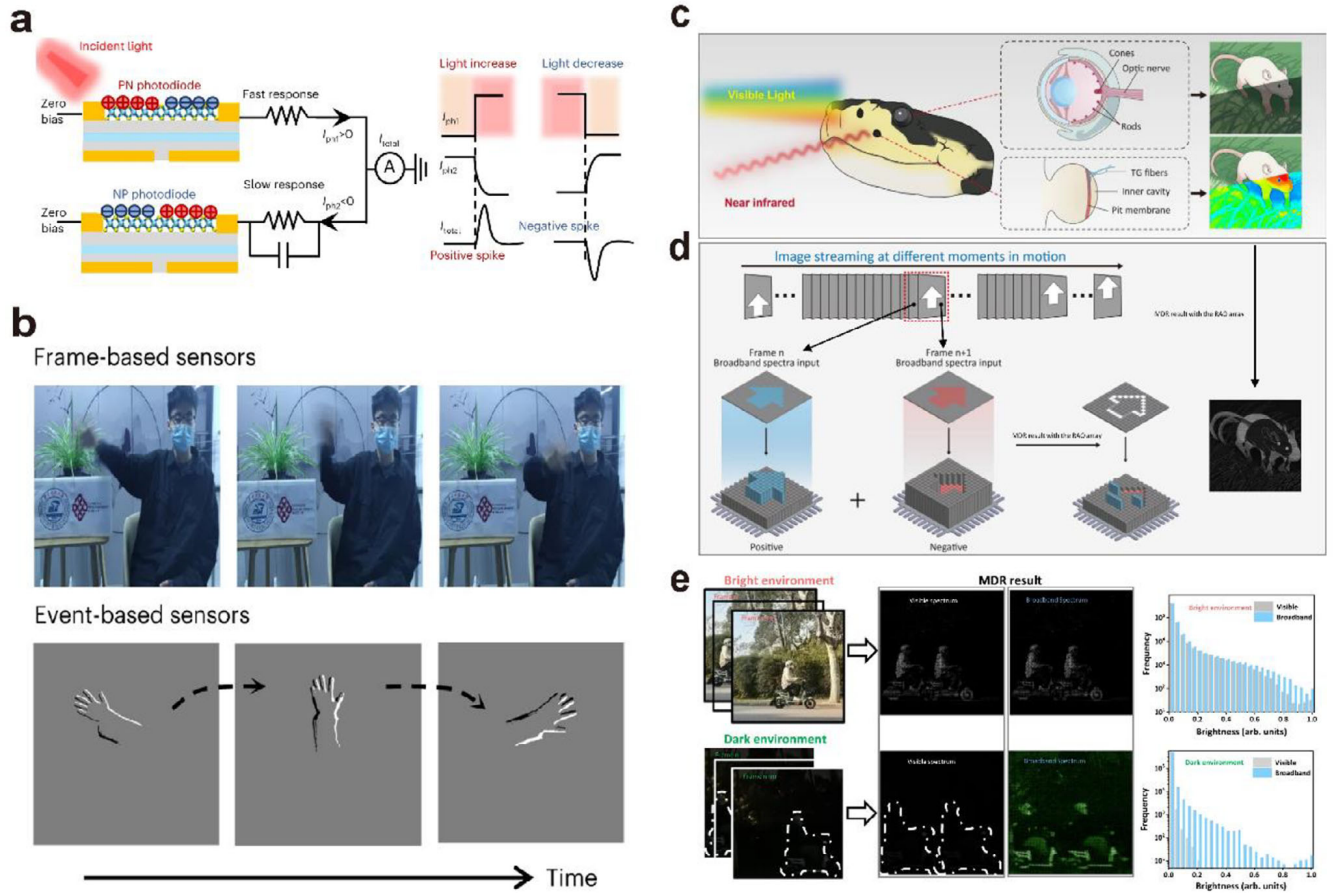
Motion detection functions of the neural vision system. a) Event‐driven visual sensor and dynamic recognition circuit design.^[^ ^133^ ^]^ b) Comparison between frame‐and event‐based vision sensors.^[^ ^133^ ^]^ c) Biological system of snakes for the detection of both visible and infrared radiation.^[^ ^52^ ^]^ d) All‐day MDR methodology inspired by the snakes.^[^ ^135^ ^]^ e) Motion detection in a bright and dark environment.^[^ ^135^ ^]^

One important approach to achieving motion detection involves designing specialized circuit units such that photocurrents generated under static conditions cancel each other out, while differences arise in dynamic currents. Zhou et al. designed the circuit structure illustrated in **Figure** **15**a.^[^ ^133^ ^]^ Under stable illumination in a static scene, the absolute values of photocurrents *I*_ph1_ and *I*_ph2_ generated by the two branches are exactly equal, resulting in a net output current (*I*_total_) of zero. When the illumination intensity changes, the two branches exhibit instantaneous and opposite photocurrent variations with differing response speeds. Specifically, upon an increase in light intensity, the photocurrent in the PN junction branch without capacitance increases, whereas that in the NP junction branch with capacitance decreases momentarily. The PN junction branch without capacitance responds faster to light stimuli than the capacitive branch. Accordingly, upon a sudden increase in light intensity, the unit generates a positive spike signal, as shown in the upper right of Figure 15a. Conversely, when the light intensity decreases, a negative spike signal is produced, as illustrated in the lower right of Figure 15a. Figure 15b demonstrates the event‐driven visual sensor performance, which asynchronously captures and responds only to local pixel‐level changes in light intensity. Gao et al.^[^ ^134^ ^]^ fabricated PdSe_2_/pentacene synaptic transistors that operate in the mid‐infrared (MIR) regime and assembled them into a 4 × 4 MIR synaptic array. When a MIR laser beam was scanned along an “L”‐shaped path, the illuminated pixels generated a photocurrent that, thanks to synaptic plasticity, decayed slowly after the light moved away, leaving a visible memory trace of the motion.

Figure 15c illustrates the dual sensory mechanism of snakes, which detect visible light through their eyes and infrared radiation via specialized organs. This dual‐sensory system enables snakes to generate a “thermal map” of moving targets under varying illumination conditions. Pang et al. designed two types of optoelectronic synaptic devices to form arrays: one exhibiting positive photoconductivity (NV‐PPC) and the other exhibiting negative photoconductivity (NV‐NPC).^[^ ^135^ ^]^ These two arrays operate alternately in time and function complementarily. The system enables effective extraction of moving targets while suppressing the background. At a given moment, the first array (NV‐PPC) captures a frame of the scene (referred to as frame n). Subsequently, at the next moment, the second array (NV‐NPC) captures the following frame (frame n+1). As shown in Figure 15d, for static objects whose positions remain unchanged between frames n and n+1, the photocurrents generated by the two arrays are equal in magnitude but opposite in sign. The total photocurrent thus nearly cancels out, resulting in dark or signal‐free regions in the final image. In contrast, for moving objects that change positions between consecutive frames, the two arrays detect the object at different locations. Consequently, the photocurrents do not fully cancel each other, and these regions appear bright or signal‐present in the final image, thereby highlighting the moving targets. Figure 15e presents the results of motion detection using visible and broadband spectra under both bright and dark environments. The results demonstrate that in bright conditions, motion detection can be effectively performed using visible light alone. In dark environments, the assistance of near‐infrared radiation enables the recognition of moving targets even when visible light is insufficient.

Synaptic devices inherently exhibit pronounced relaxation characteristics, which can be utilized to achieve dynamic perception by recording the trajectories of moving objects. The fundamental principle is that postsynaptic current decay states vary with different temporal stimulation sequences. Luo et al. developed a self‐driven biomimetic retina based on an ion gel heterostructure.^[^ ^51^ ^]^ Using a 455 nm point light source as a moving object, they collected clustering data from a 5 × 5 sensing array through an automated signal acquisition system. Real‐time trajectories of the moving light spot at different speeds were measured, and normalized voltage difference profiles were extracted from the trajectory data. This enabled velocity estimation and effective motion tracking of dynamic objects. To extract dynamic motion trajectories, Huang et al.^[^ ^22^ ^]^ designed a neuromorphic optical sensor array based on NbS_2_/MoS_2_. In this system, the photocurrent does not immediately vanish after the cessation of optical stimulation but instead decays gradually to a new equilibrium state. This time‐dependent decay characteristic enables the sensor to record the trajectory of a moving light spot. As the light spot moves along a predefined path, the photocurrent from each pixel is continuously collected, and these data are subsequently mapped into a trajectory image, thereby achieving effective motion trajectory extraction. Ni et al. integrated an electrochromic neuromorphic transistor (ENT) with an artificial antenna inspired by the longhorn beetle.^[^ ^136^ ^]^ When external forces are applied to the composite film, the conductive pathways are enhanced, allowing for the transmission of larger currents. Through continuous contact with target objects, the artificial antenna is capable of detecting regular pulse fluctuations and recording various vibration states.

Neuromorphic visual systems leveraging optoelectronic synaptic devices exhibit a range of sophisticated functionalities. High‐resolution imaging has been achieved through the integration of low‐dimensional materials with optimized device architectures, enabling precise acquisition of spatial information. Sensor‐level preprocessing further enhances image quality by amplifying salient features and suppressing background noise, exploiting material properties such as wavelength‐dependent absorption and photoconductive memory effects. Adaptive mechanisms, realized via gate modulation or intrinsically responsive materials, allow the system to adjust its sensitivity across varying illumination conditions, emulating biological photopic and scotopic adaptation. In addition, motion detection is enabled through specialized circuit designs or the inherent relaxation dynamics of synapses, permitting accurate tracking of dynamic objects while minimizing redundant data acquisition. Collectively, these approaches demonstrate how material selection, device configuration, and biomimetic strategies can be orchestrated to achieve intelligent, efficient, and multifunctional visual perception.

### 3.3 Advanced Functions of the Visual System

With the advancement of visual neuromorphic systems, their functions have extended beyond basic image recognition and recording, evolving to incorporate improvements in areas such as light and dark adaptation, motion detection, multimodal perception, and image preprocessing.

#### 3.3.1 Memory Function

**Figure 16:**
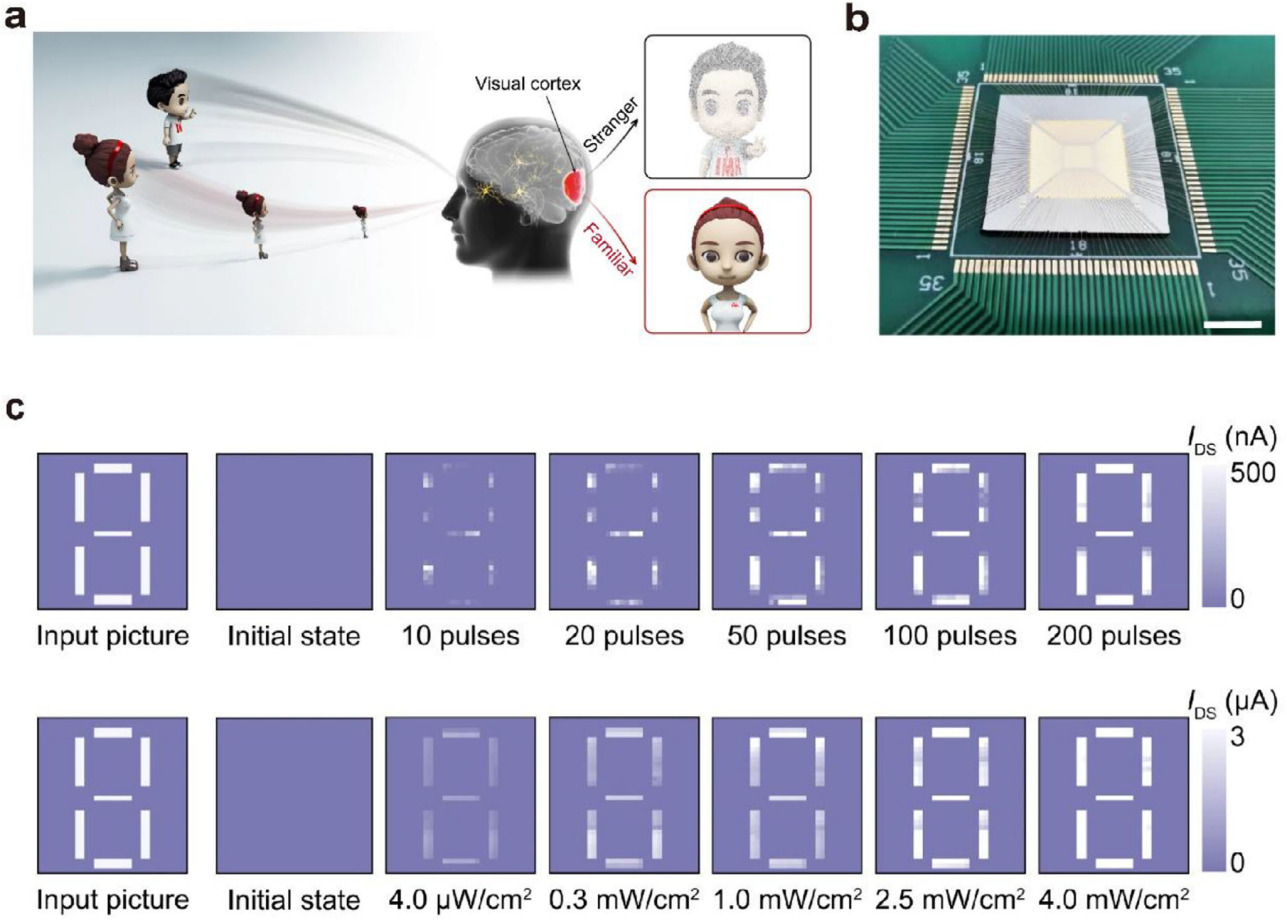
Memory function of the vision system. a) Schematics of the impression of human visual systems when strange and familiar faces are observed.^[^ ^5^ ^]^ b) A sensor array chip with wire bonding on a PCB (scale bar, 5 mm).^[^ ^5^ ^]^ c) Measured training weight results of a number 8 pattern in the initial state and after training with different pulses and power density.^[^ ^5^ ^]^

Humans tend to have clearer memories of familiar faces, while memories of unfamiliar faces seen only occasionally are relatively vague (**Figure** **16**a).^[^ ^5^ ^]^ Neuromorphic visual arrays can achieve a similar function by adjusting the weights assigned to different input signals through training. Sun and Zhu et al. constructed an ultrasensitive optoelectronic detection array by combining quantum dots with carbon nanotubes. Due to the charge trapping effect at the interface between the quantum dots and carbon nanotubes, the device exhibits not only ultrahigh sensitivity but also the basic characteristics of synapses. To explore the potential of the array in the field of artificial vision, they constructed an array device based on quantum dot/carbon nanotube transistors and integrated it onto a printed circuit board to study imaging and image memory functions (Figure 16b). Figure 16c shows the results of weight training for the digit “8” pattern under different numbers of training pulses and light intensity levels. It can be observed that the accuracy between the input image and the trained weight map improves with an increase in the number of training pulses and illumination intensity, successfully emulating the memory effect of human vision.

#### 3.3.2 Recognition and Classification Function

Studies have shown that 80%‐90% of external information acquired by humans is acquired through the visual modality. Recognition and classification are the core capabilities that enable the visual system to transition from perception to understanding and decision‐making, playing a crucial role in the survival and development of humans and other organisms. Therefore, how to realize image recognition and classification using artificial synaptic devices is a central challenge in the field of neuromorphic vision.

**Figure 17:**
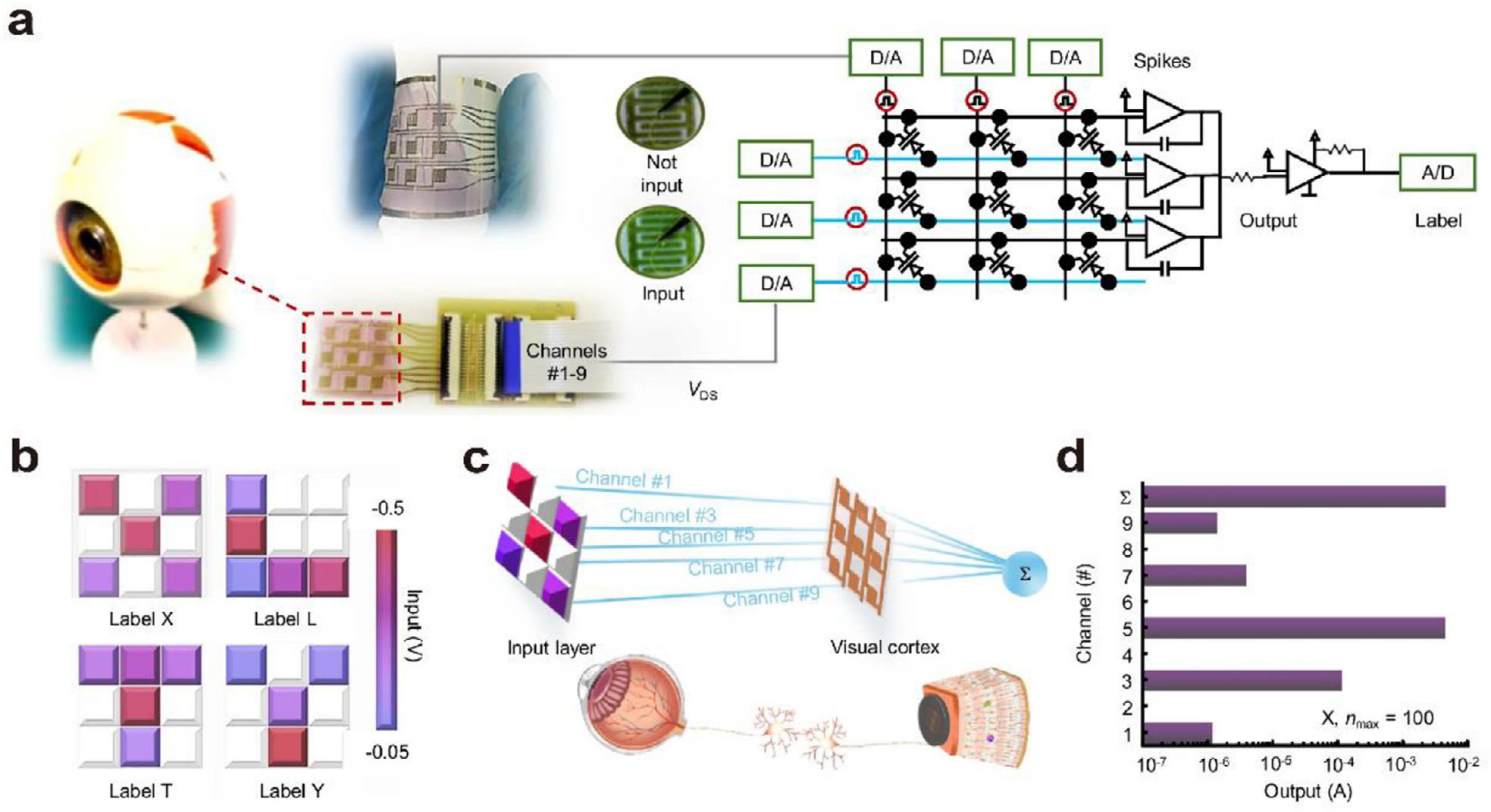
The recognition and classification functions of the vision system. a) Diagram of a 3 × 3 ENT array for visualized visual cortex.^[^ ^136^ ^]^ b,c) Classification of 3×3‐pixel images of X, L, T, and Y, where the pixels are read by *V*_DS_ of input neurons and processed by pulses from biomimetic visual cortex. d) Schematic of the response of each channel output and total output in an example of image X, under *n*_max_ = 100.^[^ ^136^ ^]^

As shown in **Figure** **17**a, Yao et al. constructed a neuromorphic visual array using electrochromic neuromorphic transistors (ENTs), composed of numerous pixels, each functioning as an independent photosensitive unit.^[^ ^136^ ^]^ These synaptic transistors utilize the organic semiconductor poly (3‐hexylthiophene) (P3HT) as the channel material and an ion gel as the dielectric layer. Each ENT operates within a crossbar array architecture, performing matrix multiplication and outer product update operations, thereby emulating the preliminary processing of visual information in biological visual systems.

The neuronal computation process consists of two main phases. 1) the training phase: receiving and transforming input signals, pulse encoding, and weight assignment; and 2) the classification and recognition phase: current response and weighted summation, activation and output, followed by classification and decision‐making. The specific process is illustrated in Figure 17b–d, where a 3 × 3 pixel image (X, L, T, Y) is input into the neural network for recognition.

##### Training Process

- Reception and conversion of input signals. The image X is first pixelated into a 3×3 grid, with each pixel corresponding to an input channel, resulting in a total of nine channels. Each pixel's grayscale value is then mapped to a voltage value (*V*_DS_) and applied as an input signal to the respective devices in the ENT array.
- Pulse encoding and weight assignment. The input signals are converted into a certain number of pulses; stronger signals correspond to a higher number of pulses. The pulse count modifies the conductivity state of the synaptic devices. Each ENT device assigns weights to the input signals based on its conductivity state, the higher the conductivity, the greater the weight assigned to that pathway.

##### Recognition Process

- Current response and weighted summation. Each ENT device generates an excitatory postsynaptic current (EPSC) based on the input pulse signals and its current conductivity state (weight). The EPSCs from all input neurons are then summed at the neuron to produce a total current representing the neuron's overall response to the input image.
- Activation and output. The total current is processed through an activation function to determine whether the neuron is activated and to what degree. The activated signal is then output from the neuron for further processing.
- Classification and decision‐making. Each neuron's output corresponds to a specific classification label. Based on the outputs of all neurons, the network makes a final classification decision, typically selecting the label corresponding to the neuron with the strongest output signal. For example, if the total output current maps to label X, it indicates that the network has recognized the input image as X.

#### 3.3.3 Multimodal Perception Function

**Figure 18:**
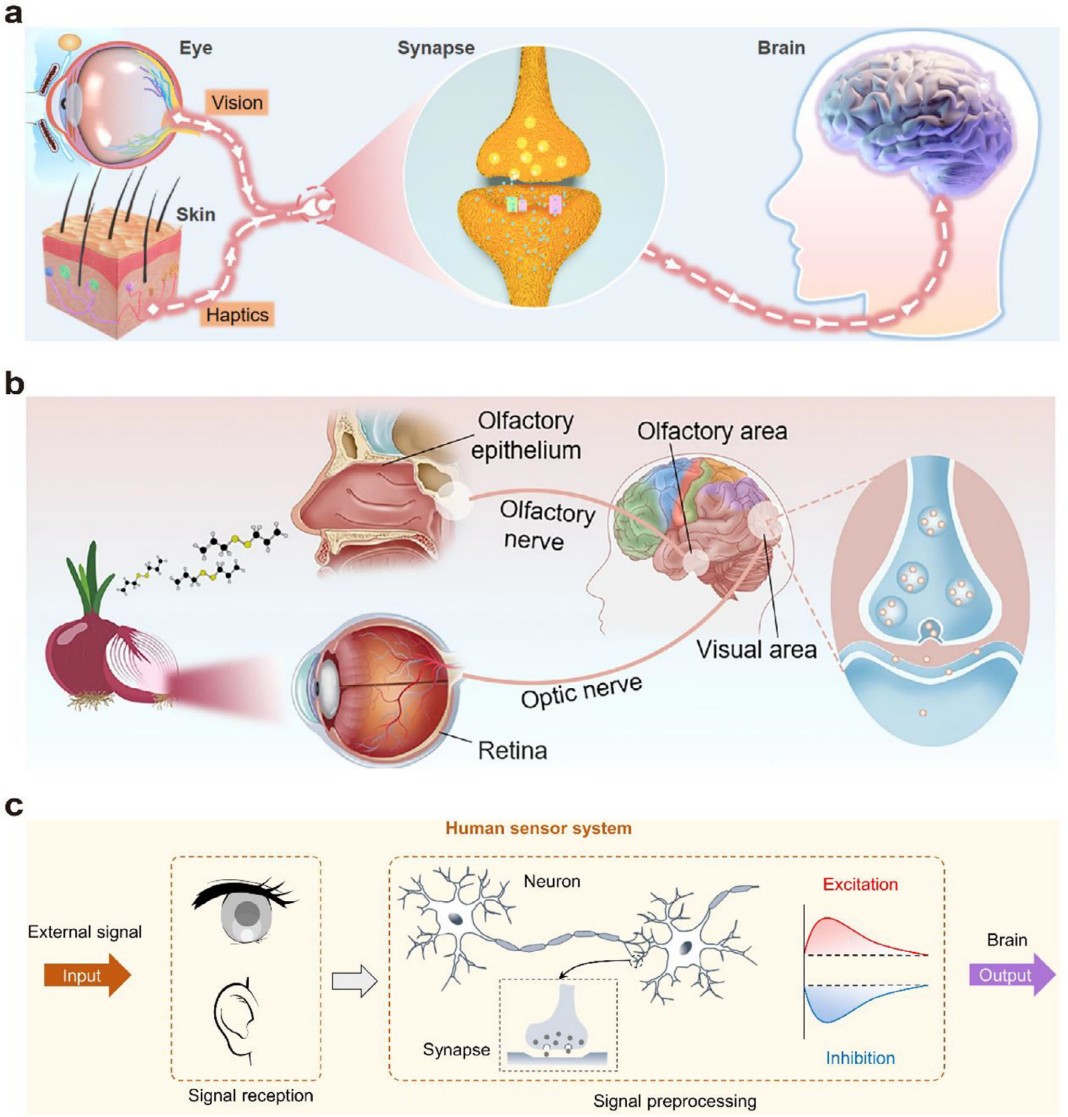
Multimodal perception functions in humans. a) Schematic diagram of visual‐tactile combined perception.^[^ ^137^ ^]^ b) Schematic diagram of visual‐olfactory combined perception.^[^ ^138^ ^]^ c) Schematic diagram of visual‐auditory combined perception.^[^ ^139^ ^]^

The multimodal perception function involves receiving and processing information through multiple sensory channels, including combinations such as vision‐audition, vision‐olfaction, and vision‐tactile perception, as illustrated in **Figure** **18**a–c. This integration enhances the accuracy of environmental understanding and decision‐making. For example, combining visual and auditory information enables more precise localization of sound sources. In situations where certain sensory inputs are limited or unreliable, multimodal systems can leverage information from other senses as a supplement, thereby improving overall system robustness.

**Figure 19:**
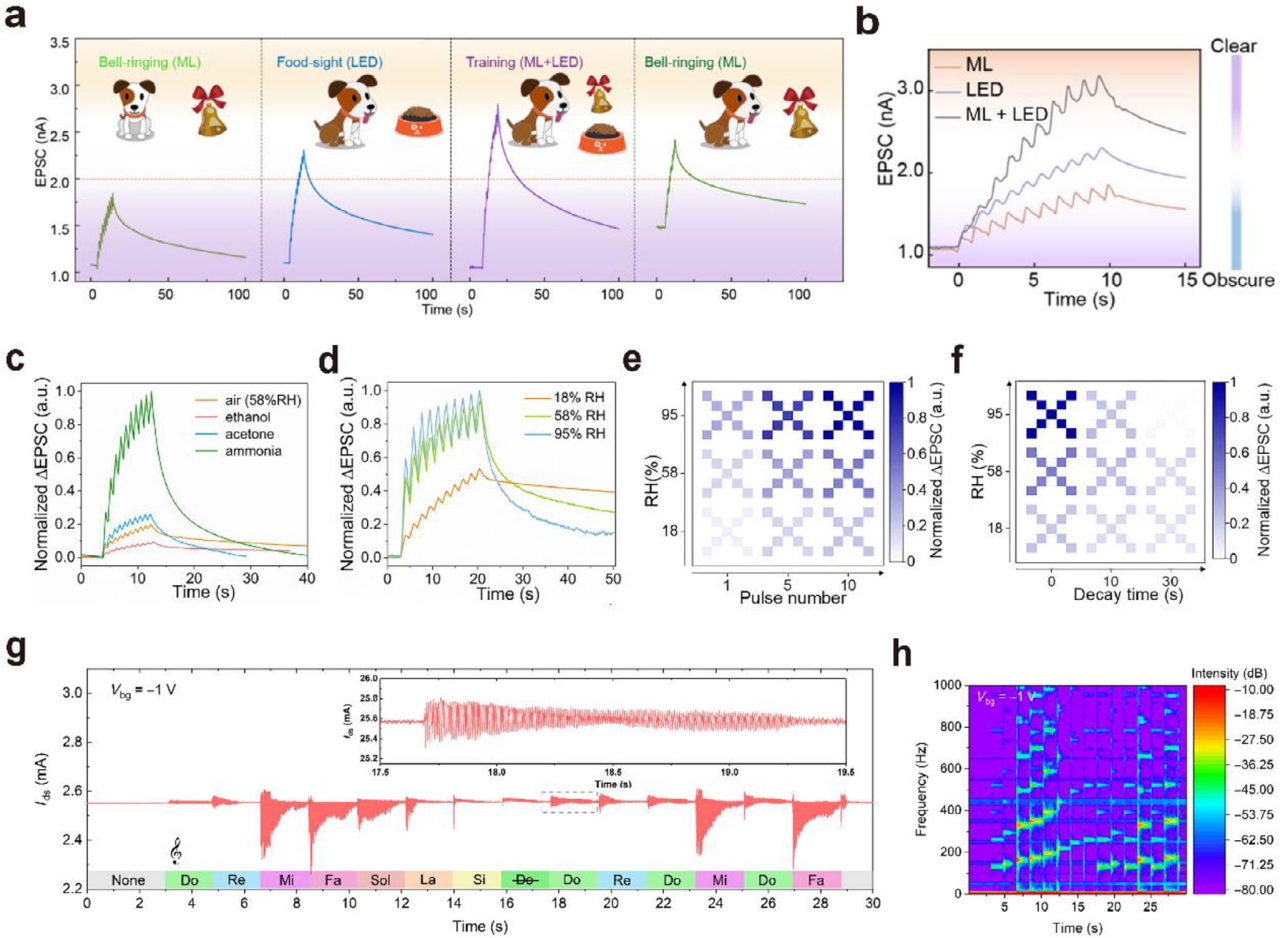
Multimodal sensory synapse devices and arrays. Panels (a) and (b) simulate synaptic tactile and optical dual sensing.^[^ ^137^ ^]^ a) An optoelectronic synapse network based on IGZO and methylammonium lead iodide (MAPbI_3_) heterostructures simulates the classic conditioned reflex experiment “Pavlov's dog”.^[^ ^137^ ^]^ b) Stimulation of a representative sensing element in the array under pressure and light stimulation conditions, respectively.^[^ ^137^ ^]^ Panels (c)–(f) mimic the dual perception of synaptic light and gaseous and humidity conditions. c) EPSC curves under different gas environments.^[^ ^138^ ^]^ d) EPSC curves under different relative humidity (RH) conditions.^[^ ^138^ ^]^ MXene/violet van der Waals heterojunction optoelectronic synapses recognize (e) and forget (f) visual information in different relative humidity (RH) environments.^[^ ^138^ ^]^ Panels (g) and (h) mimic the dual perception of synaptic visual and auditory stimuli. Specifically, panel (g) shows the response of VAPD to different frequency sound waves at a gate voltage *V*_bg_ = −1 V^[^ ^139^ ^]^; panel (h) presents the spectrum diagram, illustrating the response intensity and frequency distribution of VAPD to sound waves.^[^ ^139^ ^]^

Simultaneous application of optical and pressure stimuli enables multimodal vision‐tactile perception. **Figure** **19**a illustrates an optoelectronic synaptic network based on an IGZO/methylammonium lead iodide (MAPbI_3_) heterostructure simulating the classical conditioning experiment known as “Pavlov's dog”.^[^ ^137^ ^]^ In the experiment, a series of tactile pulses (representing the bell/conditioned stimulus) was first applied to the device, causing a slight increase in the excitatory postsynaptic current (EPSC) to 1.86 nA, which remained below the response threshold of 2 nA. Subsequently, a series of optical pulses representing the visual stimulus of food/unconditioned stimulus was applied. These optical pulses simulated the visual cue of food, leading to a significant increase in EPSC to 2.33 nA, surpassing the response threshold and mimicking the dog's salivation upon seeing food. Finally, simultaneous application of tactile and optical pulses further increased the EPSC to 2.80 nA, well above the salivation threshold. The experiment simulates the training process in which the combined stimulation of a bell and the visual cue of food enables the dog to associate the bell with the presence of food. After training, the application of tactile pulses alone (bell/conditioned stimulus) was sufficient to induce an EPSC of 2.44 nA, exceeding the response threshold. This indicates that the device had successfully established an association between the bell and the visual cue of food, mimicking the formation of a conditioned reflex. Figure 19b shows the stimulation response of a representative perceptual pixel in the array, clearly illustrating the effects of optical and pressure stimuli on the EPSC peak, as well as their combined influence. It is evident that the combined application of optical and pressure stimuli significantly enhances the response strength of the device.

The MXene/violet phosphorus van der Waals heterojunction‐based optoelectronic synapse (Figure 19c,d) enables visual‐olfactory cross‐modal perception, where MXene is a novel two‐dimensional material.^[^ ^96^ ^]^ Figure 19c,d shows EPSC curves under different gas atmospheres and relative humidity (RH) conditions, respectively, demonstrating the variation in the device's response to optical stimulation across different olfactory environments. The device exhibits the highest response peak in an ammonia atmosphere, while the response peak is the lowest in an ethanol atmosphere. Different olfactory environments (simulated by various gases) can significantly influence the optoelectronic synapse's response to visual stimuli, effectively mimicking human visual‐olfactory cross‐modal perception. The peak value of EPSC (ΔEPSC, defined as peak minus baseline) increased with increasing relative humidity, suggesting that higher humidity levels (simulating stronger odors) can enhance the initial synaptic response to light stimulation. Figure 19e,f illustrates the recognition and forgetting processes of visual information by the MXene/violet phosphorus heterojunction optoelectronic synapse under different relative humidity (RH) conditions. Under higher RH levels, the letter “X” becomes clearer after multiple stimulations, indicating a stronger recognition capability of visual patterns. However, at the same high RH, the top “X” fades rapidly within 30 s, and the EPSC decays faster, suggesting that in environments with higher odor levels, visual information is forgotten more quickly.

Fu et al. developed a vertically stacked graphene/germanium hybrid field‐effect phototransistor capable of detecting sound waves by capturing light source vibrations induced by acoustic signals, thereby enabling audio‐visual integration.^[^ ^139^ ^]^ The performance of this device in acoustic wave detection is illustrated in Figure 19g,h. Figure 19g shows the response of the biomimetic visual‐auditory photodetector (VAPD) to acoustic waves at a gate voltage of *V*_BG_ = ‐1 V. The figure clearly demonstrates that the VAPD can capture changes in photocurrent induced by sound waves. These changes reflect physical characteristics of the sound, such as frequency and intensity. Figure 19h presents a spectrogram illustrating the response intensity and frequency distribution of the VAPD to acoustic signals. The vertical axis represents the detected sound wave frequencies, with redder regions indicating stronger intensity.

Multimodal perception research has advanced by combining tactile, visual, and auditory modules with in‐sensor computing architectures. For example, Ren et al.^[^ ^140^ ^]^ developed a near‐sensor edge computing system based on a double‐layer AlN/Si photonic platform, capable of real‐time multimodal gesture and gait analysis with high precision and ultra‐low latency. Liu et al.^[^ ^141^ ^]^ reported a programmable tactile memory using a dynamic reversible filament network, achieving in‐sensor sensor‐memory integration and high‐precision motion recognition. Huang et al.^[^ ^142^ ^]^ designed a position and pressure intelligent tactile sensor that integrates feature extraction, computation, and logic to realize accurate and efficient intent‐driven interaction. Wei et al.^[^ ^143^ ^]^ proposed a reconfigurable adaptive touch sensor inspired by octopus tentacles, combined with in‐sensor integral computation, to achieve stable deformable multi‐touch recognition. Xu et al.^[^ ^144^ ^]^ introduced an event‐driven intent recognition touch sensor with in‐sensor computing capabilities, which reduced redundant data and achieved ultra‐high recognition accuracy in diverse environments. Collectively, these studies demonstrate that multimodal neuromorphic devices can integrate multiple inputs and support cross‐modal perception, thereby enhancing adaptability and enabling intelligent human‐computer interaction.

Neuromorphic visual systems have progressively expanded their functional repertoire beyond basic image acquisition, encompassing memory, recognition, and classification, and multimodal perception capabilities. Optoelectronic synaptic arrays can emulate memory effects by dynamically adjusting synaptic weights in response to repeated stimuli, enabling preferential retention of familiar patterns and enhanced image recall. Recognition and classification functionalities are realized through crossbar array architectures in which synaptic devices encode input signals as pulse trains, assign weights according to device conductivity, and generate weighted postsynaptic currents that are subsequently processed for activation and decision‐making. Multimodal perception integrates signals from multiple sensory channels, such as vision‐tactile, vision‐olfactory, and vision‐auditory inputs, allowing the system to enhance response accuracy, form associative learning, and adapt to variable environmental conditions. These advanced functions leverage material properties, heterostructure engineering, and device architecture to implement human‐like cognitive processes, providing a foundation for intelligent neuromorphic systems capable of learning, decision‐making, and adaptive environmental interaction.

## 4 Applications of Artificial Neuromorphic Visual Systems

Artificial visual systems possess significant application potential and have been widely employed across various fields, including facial recognition,^[^ ^145^ ^]^ autonomous driving,^[^ ^146^ ^]^ motion recognition in drones,^[^ ^147^ ^]^ ultraviolet light detection,^[^ ^148^ ^]^ wearable devices,^[^ ^149^ ^]^ and machine vision.^[^ ^150^ ^]^

### 4.1 Facial Recognition

**Figure 20:**
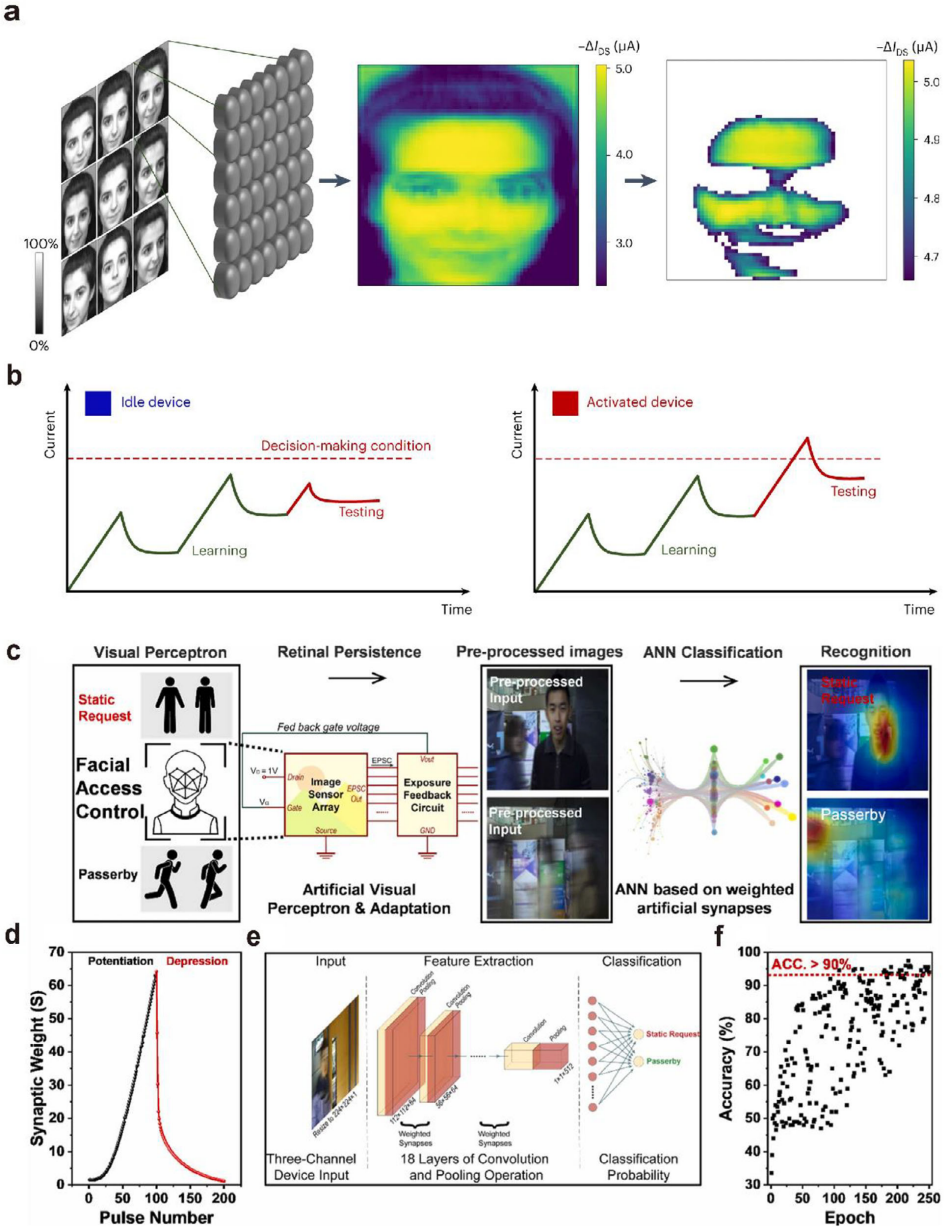
Face recognition function of synapses and visual arrays. a) Typical artificial retina model training process.^[^ ^151^ ^]^ b) Activation and inhibition mechanisms in synaptic array recognition.^[^ ^151^ ^]^ c) Cascaded face recognition access control system based on optoelectronic synapses.^[^ ^32^ ^]^ d) Enhancement and inhibition behavior of artificial neural networks in a cascaded facial recognition access control system.^[^ ^32^ ^]^ e) ANN architecture of the cascaded access system.^[^ ^32^ ^]^ f) Example of face recognition verification for different faces.^[^ ^32^ ^]^

In the field of facial recognition, neuromorphic visual systems can deliver high‐precision identification results and are widely used in applications such as identity verification and security monitoring. **Figure** **20**a illustrates the training process of an artificial retina model.^[^ ^151^ ^]^ First, nine grayscale facial images of a woman with different expressions and angles are used as optical signals and input into a simulated 64 × 64 synaptic arrays. The facial regions of these images exhibit stronger light reflection, resulting in higher memory currents and generating memory current maps that preserve facial features. Next, a subset of these memory current maps is selected—these subsets not only exhibit high memory currents but also effectively represent the facial feature contours, serving as the basis for constructing the facial recognition model. Finally, an adaptive decision threshold is set for each synaptic unit using this model. When the synaptic array perceives a facial signal similar to the training set, most synaptic units with high retained memory currents are further activated (as shown on the right side of Figure 20b), generating photocurrents that exceed the decision threshold. Conversely, signals that differ significantly from the training set result in silent responses from the synaptic units (depicted on the left side of Figure 20b).

The practical application of facial recognition in access control systems is illustrated in Figure 20.^[^ ^32^ ^]^ The system can distinguish between passersby and individuals requesting entry and performs facial recognition on the latter to determine whether to unlock the door (Figure 20c). During operation, the system relies on pre‐trained internal optoelectronic synaptic device weights. When in use, the system first captures images via sensors and then uses a low‐power ANN to perform preliminary classification on the processed images (Figure 20e), distinguishing between requesters and passersby. For images identified as requesters, the system proceeds to use a higher‐power ANN to perform facial recognition and verify the individual's identity. Based on the recognition result, the system determines whether to grant access: if the recognition is successful, the system unlocks the door; otherwise, it remains locked. Figure 20f shows the variation in classification accuracy during the ANN training process‐accuracy increases progressively with the number of training epochs. In particular, a cascaded access control system based on optoelectronic synaptic devices was demonstrated, in which visual persistence was employed to distinguish requesters from passersby. By combining a low‐power ANN for preliminary screening with a higher‐power ANN for identity verification, the system achieved more than 90% recognition accuracy for valid requesters while ignoring passersby, thus highlighting the practical feasibility of neuromorphic visual systems for real‐world facial recognition applications.

### 4.2 Autonomous Driving

During autonomous driving, neuromorphic visual systems can identify and track pedestrians, vehicles, and obstacles on the road in real time, enhancing the safety and reliability of autonomous driving systems.

**Figure 21:**
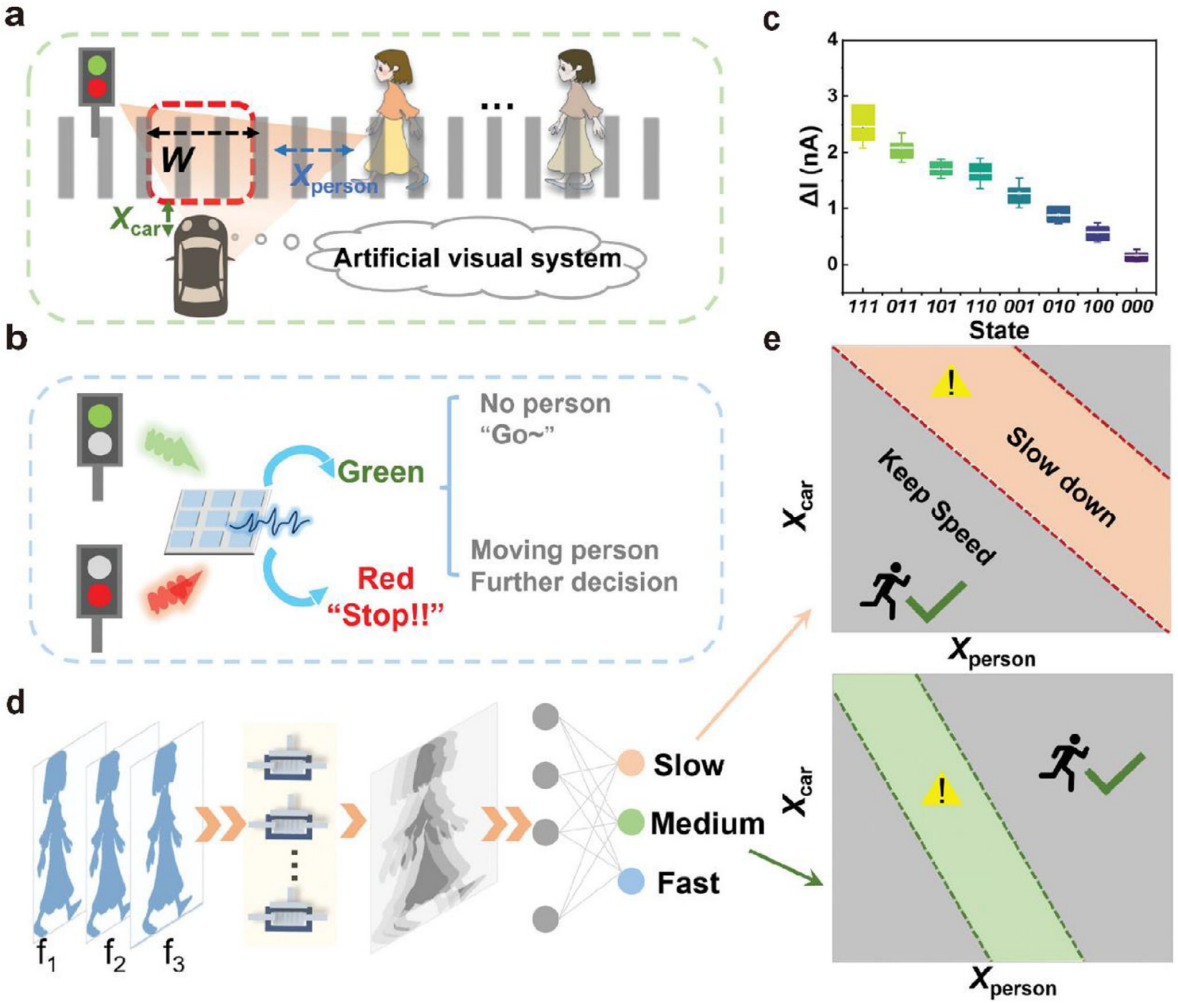
Synaptic array recognition of pedestrians. a) Schematic illustration of the traffic condition at an intersection. The area within the red‐outlined region signifies a “warning” zone where accidents may occur, with a width denoted by W.^[^ ^152^ ^]^ b) The decision‐making process of the visual system, in which traffic signals and pedestrian are both considered.^[^ ^152^ ^]^ c) Experimental outputs of 8 different 2‐bit optical pulse, ranging from (000) to (111). Each data point represents the average of 10 repeated tests (n = 10). The distinguishable states are critical for determining the speed of a moving pedestrian.^[^ ^152^ ^]^ d) Demonstration of dynamic speed inference and prediction.^[^ ^152^ ^]^ e) The decision maps of the visual system for autonomous. The highlighted region indicates that the car needs to slow down, and the coral and green areas correspond to the classification results of the pedestrian's slow and medium speed.^[^ ^152^ ^]^

Pedestrians are commonly encountered during autonomous driving, and accurately identifying them to avoid collisions represents a critical challenge. Liu et al. developed an intelligent visual system based on synaptic devices with an IGZO channel layer and a hafnium‐based HZO gate layer, and simulated the system's decision‐making performance at intersections.^[^ ^152^ ^]^ **Figure** **21**a ^[^ ^152^ ^]^ illustrates the decision‐making process of autonomous driving at an intersection, responding to traffic signals and pedestrian movement. As pedestrians move across the road, they create shadows or contours that differ in light intensity from the surrounding environment. These changes in light intensity are captured by the IGZO channel layer and converted into variations in electrical signals, allowing the system to initially detect the presence and approximate outline of pedestrians. Combined with the ferroelectric properties of the HZO gate layer, the device can simulate synaptic plasticity by adjusting the gate voltage. This enables more complex processing and analysis of optical signals, thereby enhancing the system's ability to recognize pedestrian contours.

Because IGZO, as the channel layer, exhibits different response sensitivities to different wavelengths of light, it generates varying numbers of photogenerated carriers (electron‐hole pairs) when exposed to light of different colors (i.e., wavelengths). This leads to changes in the channel current. The device can detect these current variations and thereby distinguish between different colors of light. As a result, it can identify the state of traffic signals and use this information as a basis for decision‐making (Figure 21b).

Figure 21c simulates pedestrian movement using binary‐encoded optical pulses, where faster pedestrian speeds correspond to larger current responses. By analyzing the variation of the transistor's current response over time, the system can infer the pedestrian's walking speed (Figure 21d). When a vehicle encounters a pedestrian within the “warning” zone, it will proactively decelerate or stop in advance to avoid a collision (Figure 21e).

**Figure 22:**
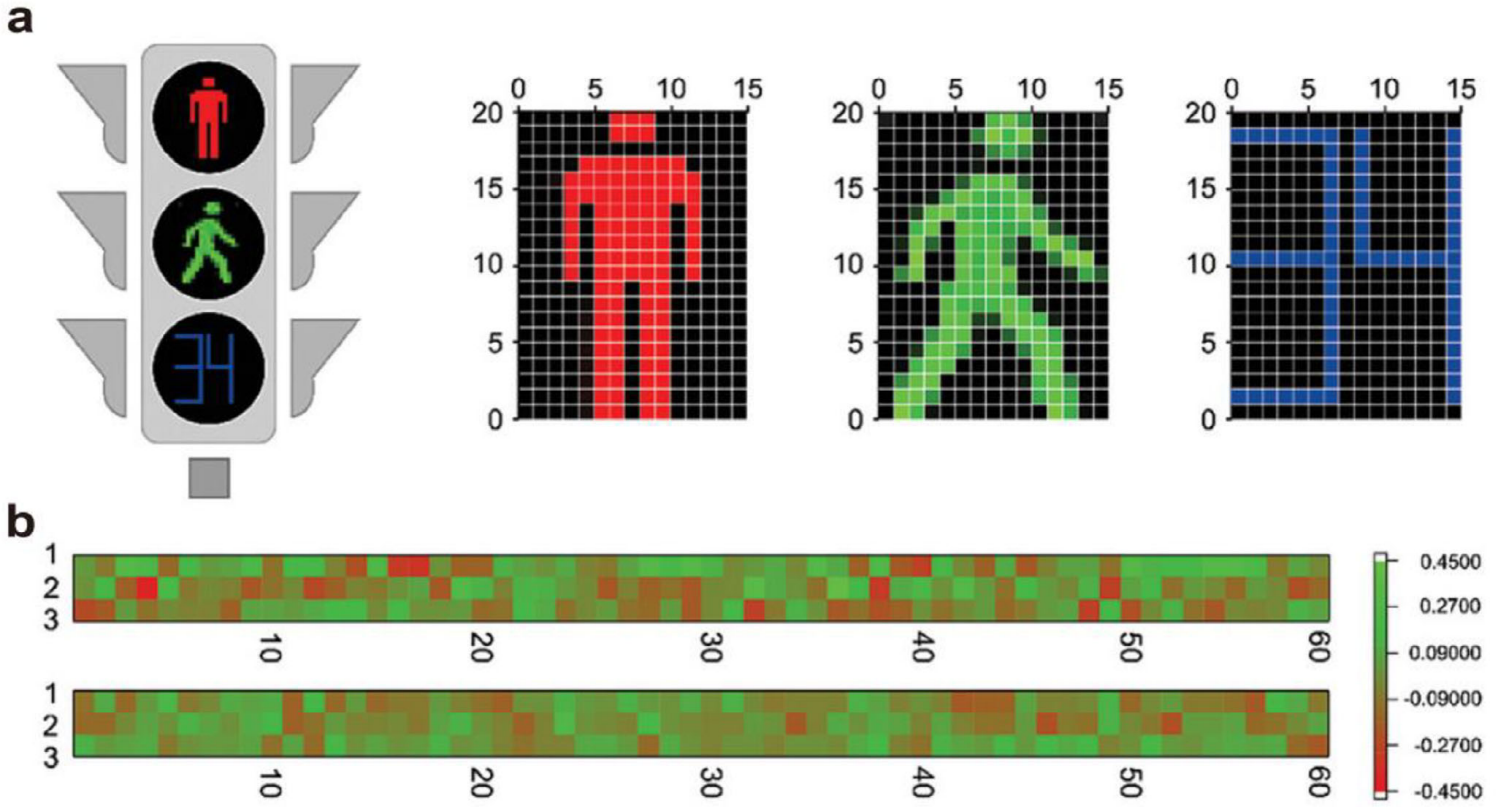
Synaptic array recognition of traffic lights. a) Traffic light images used for RC training during autonomous driving.^[^ ^153^ ^]^ b) Weight distribution of the output layer of the RC system after multiple training cycles.^[^ ^153^ ^]^

Traffic light recognition serves as a fundamental component of autonomous driving. In the sensor‐integrated reservoir computing (RC) system, traffic light images illuminated under red, green, and blue light conditions are used as training data (**Figure** **22**a).^[^ ^153^ ^]^ Figures 22b (top and bottom) show the weight distribution of the readout layer under electrical and optical stimulation, respectively, after 100 training cycles. The labels “1,” “2,” and “3” correspond to red, green, and blue lights. The numbers 10, 20, 30, etc., represent nodes in the input layer, with each node corresponding to a pixel position in the image‐essentially a synaptic unit. Darker colors (such as red or yellow) indicate higher weight values, while lighter colors (such as blue or green) indicate lower weight values. For example, the first column's three‐pixel blocks labeled 1, 2, and 3 show weak, weak, and strong responses, respectively, indicating that this position is particularly sensitive to blue signals.

**Figure 23:**
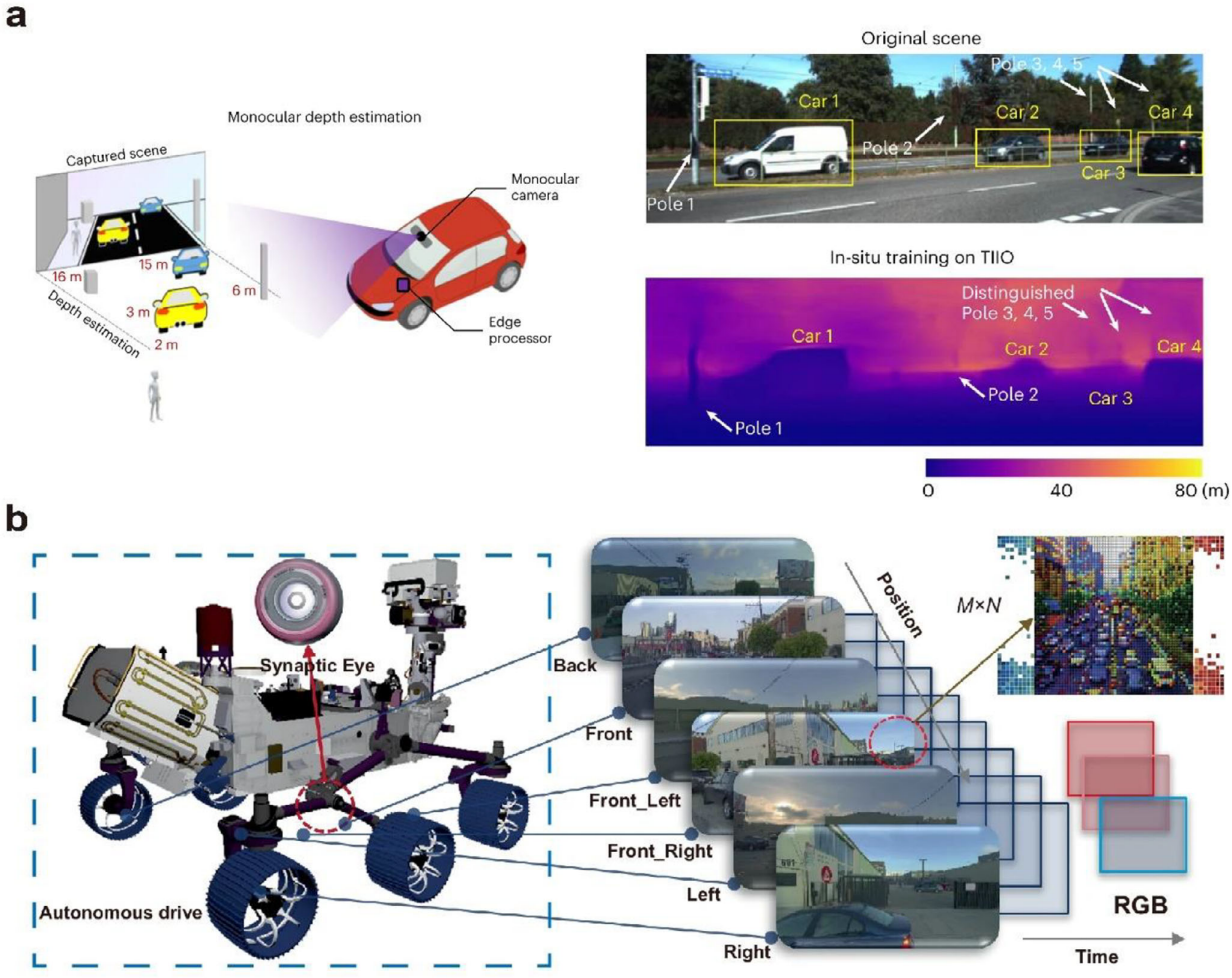
Synaptic array for vehicle and environment recognition. a) Schematic of monocular depth estimation applicable to stereo vision in autonomous driving, and an example of monocular depth estimation.^[^ ^90^ ^]^ b) Application of optoelectronic synapse arrays with 360‐degree environment sensing capability in autonomous driving simulation.^[^ ^154^ ^]^

In autonomous driving applications, stereo vision or multiple cameras are typically used to capture 3D information of the surrounding environment. Figure 23a illustrates a monocular depth estimation scenario in autonomous driving.^[^ ^90^ ^]^ Monocular depth estimation is a more cost‐effective approach with lower hardware requirements; it relies on images captured by a single camera and employs deep learning algorithms to infer the depth information of the scene. The right side of **Figure** **23**a shows an actual example, where the synaptic array successfully identified all features and captured their relative depths with a convergence speed comparable to that of a graphics processing unit (GPU). The recognition accuracy and root mean square error (RMSE) at the 3σ confidence level reached 96.85% and 6.31%, respectively.

An application example of a optoelectronic synaptic array in autonomous driving simulation is shown in Figure 23b.^[^ ^154^ ^]^ The vehicle's autonomous driving system is based on a SWCNTs/ZnTPP optoelectronic synaptic array. The vehicle is equipped with six visual sensors placed at different positions‐rear, front, front‐left, front‐right, left side, and right side‐to collect environmental information, providing the vehicle with 360‐degree situational awareness. The right side of Figure 23b presents the datasets collected from these visual sensors. The captured images are decomposed into red, green, and blue (RGB) color channels and converted into pulse signals processed by the synaptic array, enabling neuromorphic computing and autonomous driving decision‐making. Due to ZnTPP's high optical absorption coefficient, it can rapidly respond to optical signals and effectively convert them into electrical signals. Consequently, during autonomous driving, the vehicle can promptly perceive changes in the surrounding environment‐such as road conditions, traffic signals, and obstacles‐enabling faster and more accurate decision‐making and responses. This capability significantly enhances driving safety and efficiency. When utilizing spiking neural networks (SNN) to simulate the autonomous driving process, the system maintains a recognition accuracy above 90%.

Artificial neuromorphic visual systems have demonstrated extensive applicability across multiple domains, leveraging their ability to emulate human‐like perception, memory, and decision‐making processes. In facial recognition, synaptic arrays encode facial features as memory currents, enabling accurate identification and adaptive threshold‐based decision‐making. Integration with artificial neural networks further enhances system performance, allowing reliable verification in access control applications. In autonomous driving, neuromorphic visual systems facilitate real‐time detection and tracking of pedestrians, vehicles, and traffic signals. Devices incorporating materials such as IGZO and hafnium‐based HZO layers convert variations in light intensity and color into electrical signals, enabling precise estimation of pedestrian speed, traffic light state recognition, and depth inference. Optoelectronic synaptic arrays with multi‐sensor configurations provide 360‐degree environmental awareness, converting RGB image information into spiking signals for neuromorphic computation and rapid decision‐making. These capabilities collectively enhance the safety, efficiency, and responsiveness of autonomous systems, highlighting the potential of artificial neuromorphic vision in complex, real‐world scenarios.

## 5 Conclusion and Outlook

With the advancement of the third technological revolution, key technologies such as semiconductors and artificial intelligence have become focal points in the field of science and technology, gaining increasing importance and attention. Neuromorphic visual systems, as a key technology at the intersection of semiconductors and artificial intelligence, rely on optoelectronic synaptic devices integrating sensing, memory, and computing functions. These systems enable the direct input, in situ storage, and processing of real‐world visual information within computing platforms. Research on neuromorphic visual systems not only facilitates the imitation of biological synaptic mechanisms within the semiconductor domain but also promotes efficient in situ perception, storage, and computation. On the other hand, it helps align with the computational methods of neural networks in the field of artificial intelligence, promoting intelligent development. This study mainly discusses two perspectives: optoelectronic synaptic devices and neuromorphic visual systems. The section on optoelectronic synaptic devices covers materials, structures, and performance evaluation metrics. The section on neuromorphic visual systems introduces fundamental functions, advanced features, and practical application areas, elaborating on the corresponding working principles and the progress made to date. The summary and outlook on neuromorphic visual systems will focus on the following four aspects:

1. Development of synaptic materials. The materials for the channels and dielectric layers of optoelectronic synapses are diverse and complex, but the overall goal is to select channel materials with strong photoelectric effects to efficiently generate carriers, and to choose appropriate dielectric materials that match carrier trapping and other mechanisms to achieve synaptic plasticity. However, for devices based on photogenerated carrier trapping and release mechanisms, high‐temperature operating environments often degrade their retention characteristics. Therefore, researching materials with higher stability will be one of the key focuses in future studies. Potential directions include optimizing device encapsulation to shield the active layers from external stresses, enhancing interface engineering to suppress defect‐related instabilities, and designing composite dielectric or channel structures capable of maintaining retention performance under harsh operating conditions.
2. Synaptic functions and integration. Optoelectronic synapses have diverse and complex structures, but they can generally be categorized into basic architectures such as hybrid heterojunctions, planar heterojunctions, and floating gates. Building upon these, advanced designs like vertical cross structures and strain‐induced configurations have been developed. These structures better leverage the fundamental mechanisms of carrier generation, transport, and recombination. Currently, single devices are gradually transitioning from two‐terminal to three‐terminal configurations. However, compared to the relatively mature two‐terminal memristor arrays, the design of three‐terminal optoelectronic synapse arrays and their future application in ultra‐large‐scale integrated circuits still require more in‐depth research. Additionally, existing optoelectronic synapse devices face signal crosstalk issues when arrayed, which affects the accuracy and efficiency of information processing. Addressing crosstalk is one of the key challenges for the future development of these devices. Future research may explore circuit‐level architectures and layout schemes that minimize parasitic coupling, isolation, or shielding layers to suppress unwanted interactions, adaptive signal correction algorithms to mitigate residual interference, and 3D integration approaches to simultaneously improve device density and reduce lateral crosstalk.
3. Performance of neuromorphic visual systems. The research on neuromorphic visual systems ultimately aims at practical applications, which rely heavily on the functionalities these systems provide. In recent years, although some scholars have conducted comprehensive reviews on the performance of optoelectronic synaptic devices, systematic classification and summarization of the functionalities of neuromorphic visual systems remain limited. This work summarizes key research focuses on synaptic device functionalities by addressing foundational capabilities such as recognition, image preprocessing, visual adaptation, and motion detection, as well as advanced functions including memory, classification, and motion tracking. Nevertheless, current image perception and recognition accuracy still face significant challenges. Enhancing the efficiency and precision of visual image recognition is a critical direction for the future development of neuromorphic visual systems. Promising avenues may involve combining hardware‐based synaptic functions with machine learning algorithms, implementing hierarchical architectures that mimic multi‐layer biological vision, and adopting noise‐robust training strategies to enhance recognition accuracy and reliability under complex environmental conditions.
4. Applications of neuromorphic visual systems. This paper provides a concise overview of how machine vision can be realized through device arrays and integrated systems, while summarizing specific application scenarios. It aims to offer researchers a systematic framework spanning devices, arrays, functionalities, and applications. Currently, there is limited research on the working mechanisms and practical application scenarios of neuromorphic visual systems and synaptic arrays. However, with the advent of the intelligent era and the rapid development of robotics, exploring the applications of neuromorphic visual systems in robotics, unmanned aerial vehicles (UAVs), and virtual reality holds significant promise and represents a key focus for future research.

## Conflict of Interest

The authors declare no conflict of interest.
